# Single-cell multiome of the human retina and deep learning nominate causal variants in complex eye diseases

**DOI:** 10.1016/j.xgen.2022.100164

**Published:** 2022-07-27

**Authors:** Sean K. Wang, Surag Nair, Rui Li, Katerina Kraft, Anusri Pampari, Aman Patel, Joyce B. Kang, Christy Luong, Anshul Kundaje, Howard Y. Chang

**Affiliations:** 1 Center for Personal Dynamic Regulomes, Stanford University, Stanford, CA, USA; 2 Department of Ophthalmology, Stanford University School of Medicine, Stanford, CA, USA; 3 Department of Computer Science, Stanford University, Stanford, CA, USA; 4 Department of Biomedical Informatics, Harvard Medical School, Boston, MA, USA; 5 Center for Data Sciences, Brigham and Women’s Hospital, Boston, MA, USA; 6 Department of Chemical and Systems Biology, Stanford University School of Medicine, Stanford, CA, USA; 7 Department of Genetics, Stanford University School of Medicine, Stanford, CA, USA; 8 Howard Hughes Medical Institute, Stanford University, Stanford, CA, USA; 9 Lead contact

## Abstract

Genome-wide association studies (GWASs) of eye disorders have identified hundreds of genetic variants associated with ocular disease. However, the vast majority of these variants are noncoding, making it challenging to interpret their function. Here we present a joint single-cell atlas of gene expression and chromatin accessibility of the adult human retina with more than 50,000 cells, which we used to analyze single-nucleotide polymorphisms (SNPs) implicated by GWASs of age-related macular degeneration, glaucoma, diabetic retinopathy, myopia, and type 2 macular telangiectasia. We integrate this atlas with a HiChIP enhancer connectome, expression quantitative trait loci (eQTL) data, and base-resolution deep learning models to predict noncoding SNPs with causal roles in eye disease, assess SNP impact on transcription factor binding, and define their known and novel target genes. Our efforts nominate pathogenic SNP-target gene interactions for multiple vision disorders and provide a potentially powerful resource for interpreting noncoding variation in the eye.

## In brief

Wang et al. present a joint single-cell atlas of gene expression and chromatin accessibility of the adult human retina, which they use to analyze risk variants from genome-wide association studies of five eye diseases. They integrate this atlas with chromatin conformation data, expression quantitative trait loci, and deep learning models to nominate gene and cellular targets in the retina for hundreds of noncoding variants implicated in vision disorders.

## Graphical abstract

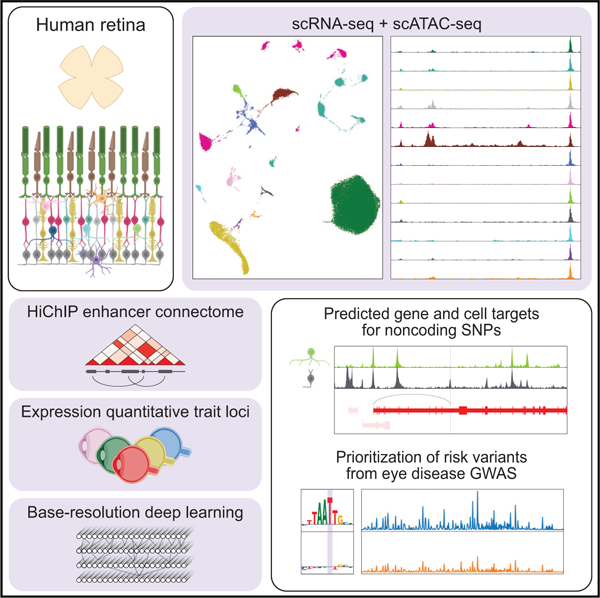

## INTRODUCTION

Genome-wide association studies (GWASs) of eye disorders, such as glaucoma, myopia, and age-related macular degeneration (AMD), have uncovered hundreds of genetic polymorphisms associated with ocular disease.^1–5^ However, the vast majority of variants identified by GWASs reside in noncoding regions of the genome, making it challenging to interpret their function.^6^ To better understand how noncoding variants mechanistically contribute to ocular pathology, it would be valuable to map in which cell types their corresponding loci are active. This information would provide novel insights into the cellular biology of genetically complex eye diseases and help nominate specific cell types as targets for therapies.

A recent advance in studying the noncoding genome has been the development of single-cell multiomics technologies, such as paired single-cell RNA sequencing (scRNA-seq) and single-cell assay for transposase-accessible chromatin sequencing (scATAC-seq). While scRNA-seq can classify the different cell types of a tissue based on their transcriptional profiles, its combination with scATAC-seq allows for the additional mapping of cell-type-specific chromatin accessibility. Together, these techniques can reveal the activity of noncoding DNA elements identified by GWASs and have been used to interrogate risk variants for conditions such as Alzheimer disease, Parkinson disease, autism spectrum disorder, and autoimmunity.^7–9^

Investigations of the noncoding genome have likewise benefitted from analytical innovations, such as application of convolutional neural network (CNN)-based deep learning to predict the effects of noncoding polymorphisms.^10–12^ Progress in this area has recently led to models with resolution down to a single nucleotide, enabling accurate determination of the critical bases within *cis*-regulatory sequences.^8,12,13^ These models offer a validated approach to prioritize noncoding variants with functional relevance and are particularly suitable for tissues in which experimental manipulation is difficult.

Here we generated a joint scRNA- and scATAC-seq atlas of the adult human retina composed of more than 50,000 cells and used it to analyze single-nucleotide polymorphisms (SNPs) implicated by GWAS of five eye diseases: AMD, glaucoma, diabetic retinopathy (DR), myopia, and type 2 macular telangiectasia (MacTel). Layering this atlas with a HiChIP enhancer connectome,^14^ expression quantitative trait locus (eQTL) data,^15^ and base-resolution deep learning models,^12^ we predicted noncoding SNPs with causal roles in eye disease. Our efforts nominate pathogenic SNP-target gene interactions for multiple vision disorders and provide a potentially powerful resource for interpreting noncoding variation in the eye.

## RESULTS

### Single-cell multiomics reveal the gene expression and chromatin accessibility landscapes of cell types in the human retina

**Figure 1.**
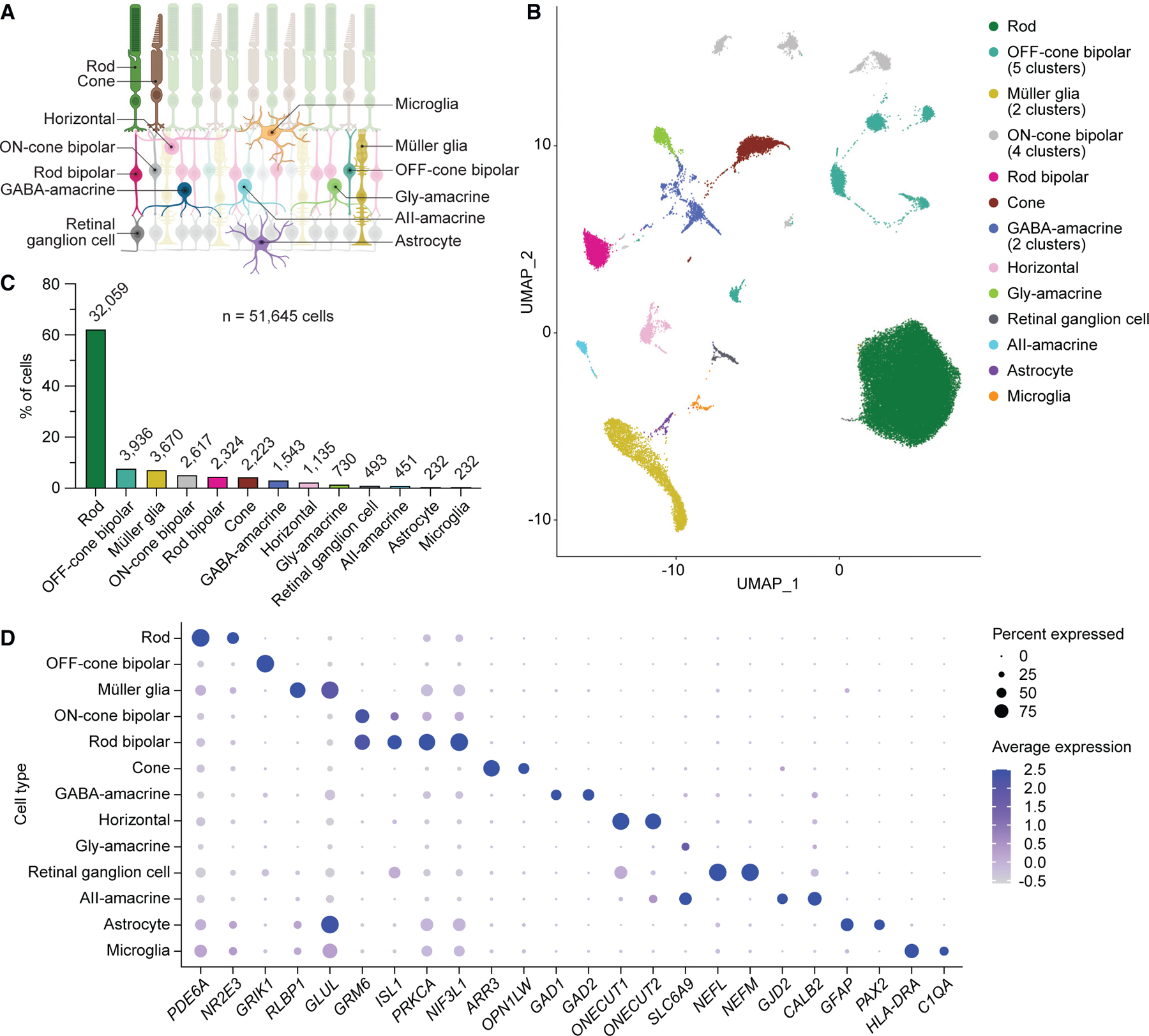
Transcriptional profiles from joint scRNA- and ATAC-seq identify major cell types of the human retina (A) Schematic of the human retina, depicting the cell types analyzed in this study. (B) Uniform manifold approximation and projection (UMAP) plot of the 51,645 human retinal cells detected by scRNA-seq after quality control filtering and removal of putative doublets. Eight postmortem retinas from four donors were profiled. A total of 22 clusters were resolved and assigned to 13 cell types. (C) Frequency of different cell types in the human retina as determined by scRNA-seq. Numbers above each bar denote absolute counts out of 51,645. (D) Dot plot visualizing the normalized RNA expression of selected marker genes by cell type. The color and size of each dot correspond to the average expression level and fraction of expressing cells, respectively.

To generate a single-cell multiome of the human retina, we performed joint scRNA- and scATAC-seq profiling on eight postmortem retinas from four individuals who had no history of eye disease (Table S1). After quality control filtering (Figure S1) and removal of putative doublets (Figures S2A and S2B), we obtained a total of 51,645 human retinal cells in 22 clusters that we assigned to 13 different cell types (Figures 1A, 1B, and S2C). These included abundant cell types, like rod photoreceptors and Müller glia, as well as rarer cell types, such as astrocytes and microglia, which each constituted only 0.4% of profiled cells (Figure 1C; Table S2). Consistent with published scRNA-seq studies of the human retina,^16–19^ we observed cell-type-specific expression of many genes, including *PDE6A* in rod photoreceptors, *GRIK1* in OFF-cone bipolar cells, *RLBP1* in Müller glia, *GRM6* in ON-cone and rod bipolar cells, *PRKCA* in rod bipolar cells, *ARR3* in cone photoreceptors, *GAD1* in GABAergic (GABA-) amacrine cells, *ONECUT1* in horizontal cells, *SLC6A9* in AII- and other glycinergic (gly-) amacrine cells, *NEFL* in retinal ganglion cells, *GJD2* in AII-amacrine cells, *GFAP* in astrocytes, and *C1QA* in microglia (Figure 1D and S3A). We also identified a list of candidate marker genes based on differential expression for each of the 13 cell types (Data S1).

**Figure 2.**
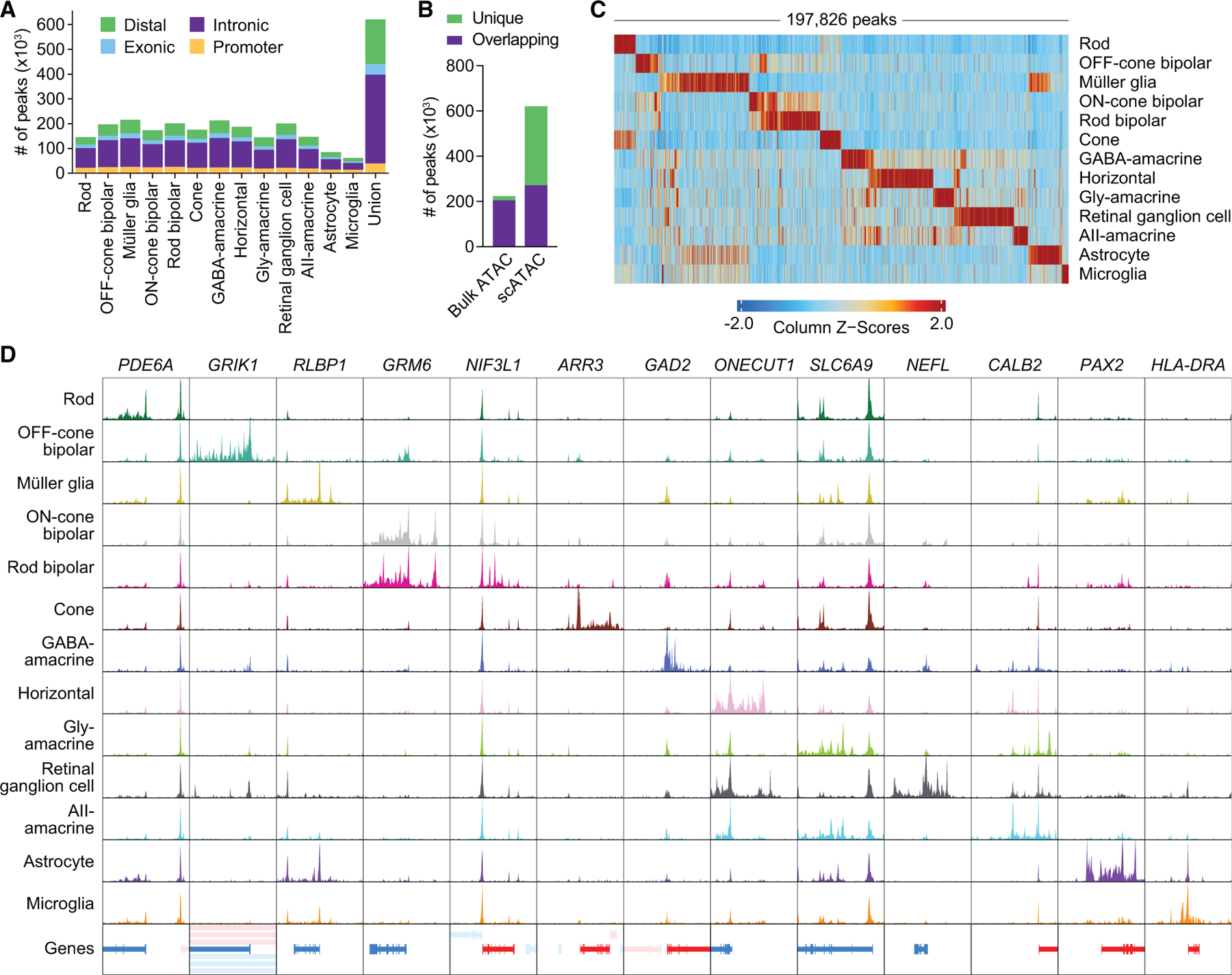
Chromatin accessibility profiles from joint scRNA- and ATAC-seq of the human retina reveal cell-type-specific epigenetic landscapes (A) Number of chromatin accessibility peaks for each cell type as determined by scATAC-seq. Peaks were required to be present in a least two pseudo-bulk ATAC replicates (n = 2 for astrocyte and microglia, n = 5 for all other cell types). (B) Overlap of scATAC peaks with peaks from published human retina bulk ATAC-seq data. Overlapping was defined as peaks with any overlapping bases. (C) Heatmap of scATAC marker peaks enriched in each cell type. Each column represents a marker peak. (D) Sequencing tracks of chromatin accessibility near selected marker genes by cell type. Each track represents the aggregate scATAC signal of all cells from the given cell type normalized by the total number of reads in TSS regions. Genes in the sense direction (TSS on the left) are shown in red, and genes in the antisense direction (TSS on the right) are shown in blue. Coordinates for each region are as follows: *PDE6A* (chr5:149924792–149964793), *GRIK1* (chr21:29905031–29955033), *RLBP1* (chr15:89201750–89241751), *GRM6* (chr5:178975297–179015298), *NIF3L1* (chr2:200874325–200914327), *ARR3* (chrX:70248304–70288305), *GAD2* (chr10:26186306–26246307), *ONECUT1* (chr15:52781076–52821078), *SLC6A9* (chr1:44005465–44035467), *NEFL* (chr8:24937109–24977110), *CALB2* (chr16:71323711–71368713), *PAX2* (chr10:100715602–100755603), and *HLA-DRA* (chr6:32419841–32459842).

Using shared barcodes from joint multiomics profiling, we next assigned scATAC-seq profiles to the 13 cell types characterized by scRNA-seq. The resulting chromatin accessibility profiles clustered by cell type (Figure S4A) and were highly similar to those from a public human retina scATAC-seq dataset (Figure S3B), supporting the validity of our multiome. Peak calling performed on scATAC-seq profiles from each cell type combined into pseudo-bulk ATAC replicates uncovered a total of 620,386 chromatin accessibility peaks (Figure 2A; Data S2). These scATAC peaks were concordant among donors (Figures S4B and S4C) and included more than 90% of peaks from a published bulk ATAC-seq study of the human retina (Figures 2B),^20^ indicating that single-cell multiomics can recapitulate bulk ATAC-seq data. Conversely, more than half of the scATAC peaks were unique to the single-cell dataset (Figure 2B), and nearly 40% of scATAC peaks were accessible in only one cell type (Figure S5A). In line with this, we found 197,826 scATAC marker peaks enriched in a cell-type-specific manner (Figure 2C; Data S3), including many located near cell-type-specific genes (Figure 2D).

**Figure 3.**
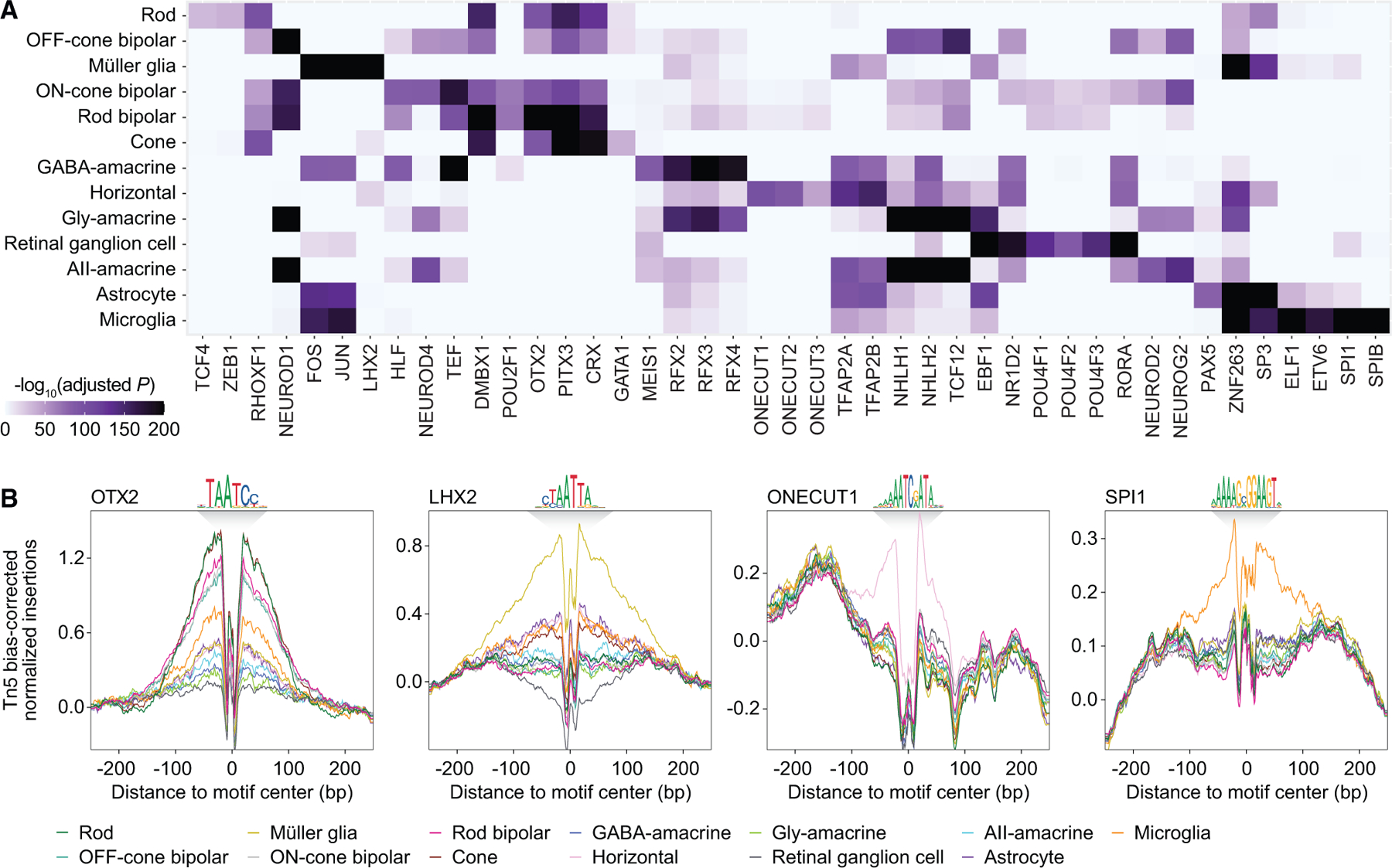
Motif analysis of accessible DNA regions in the human retina predicts cell-type-specific TFs (A) Heatmap of selected TF binding motifs enriched in each cell type. Darker colors indicate more significant enrichment. (B) Footprinting analysis of selected TFs across cell types. Footprints were corrected for Tn5 insertion bias by subtracting the Tn5 insertion signal from the footprinting signal.

With these scATAC peaks, we conducted a motif enrichment analysis to predict which transcription factors (TFs) might be active in each cell type (Figure 3A; Data S4). In accordance with published literature, we observed enrichment of binding motifs for TFs with known cell-type-specific functions, such as OTX2 in photoreceptors and bipolar cells,^21^ ONECUT family members in horizontal cells,^22^ POU4F family members in retinal ganglion cells,^23^ and SPI1 (PU.1) in microglia.^24^ For some TFs, cell-type-specific activity was also supported by footprinting analysis of scATAC peaks (Figure 3B), which revealed motif centers to be protected from Tn5 transposition, consistent with TF occupancy. These data offer a cell-type-specific catalog of candidate TFs in the adult retina and may aid our understanding of gene-regulatory networks controlling vision.

### Single-cell multiomics uncover the cellular contexts of variants implicated by ocular disease GWASs

**Figure 4.**
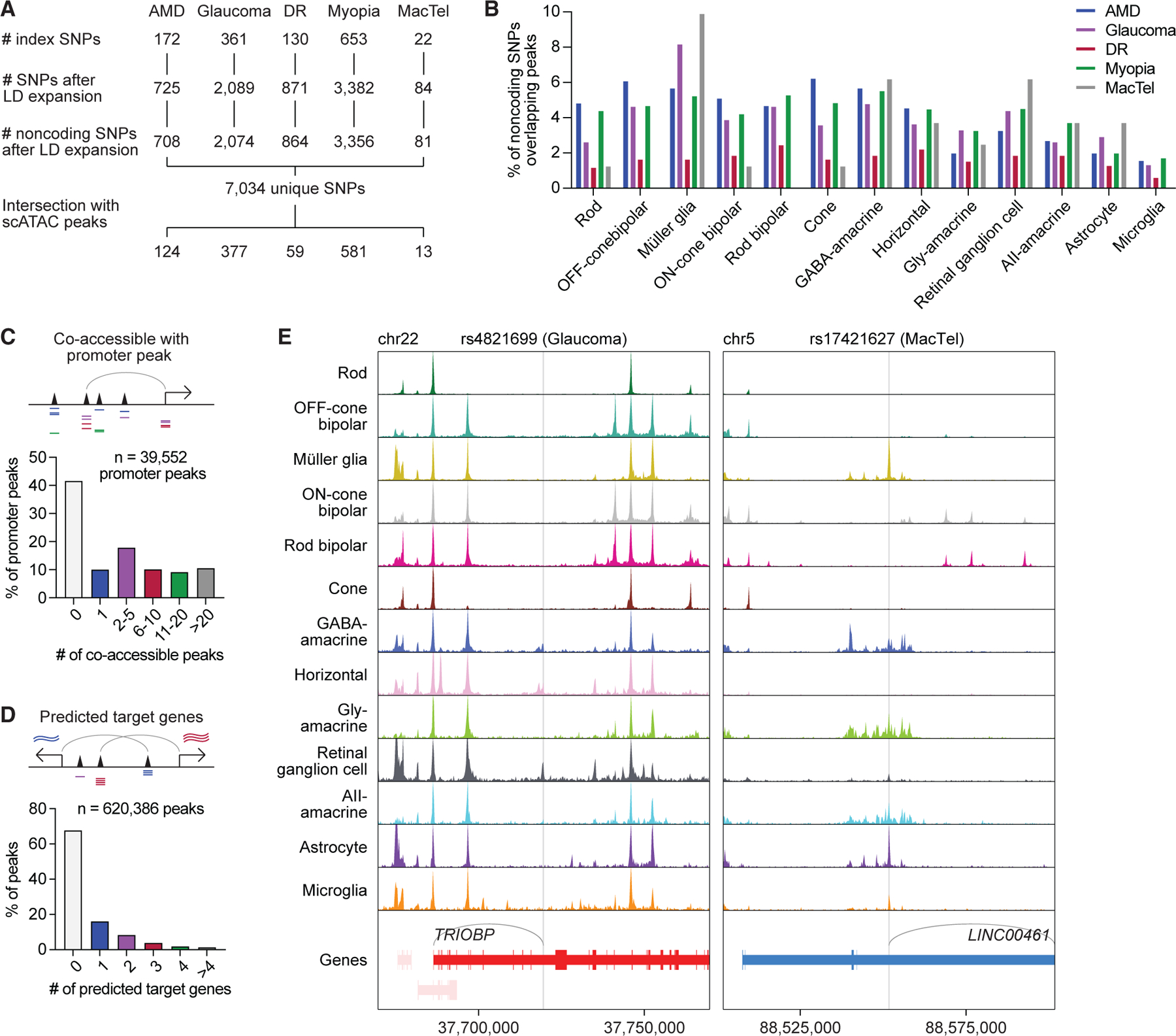
Single-cell multiomics pinpoints the cellular targets of noncoding variants in eye diseases (A) Overview of SNP selection for interrogating ocular disease GWASs. Index SNPs obtained from GWASs of each disease were subjected to LD expansion, and the resulting noncoding SNPs intersected with scATAC peaks. (B) Percentage of LD expanded noncoding SNPs from each disease that overlapped with chromatin accessibility peaks for each cell type. (C) Number of scATAC peaks co-accessible with each promoter peak. Co-accessible was defined as scATAC peaks whose accessibility showed a correlation score greater than 0.3. (D) Number of predicted target genes for each scATAC peak. Predicted target genes were defined as genes whose RNA expression showed a correlation score >0.3 relative to the accessibility of the tested scATAC peak. (E) Sequencing tracks of chromatin accessibility near rs4821699 (chr22:37719685) and rs17421627 (chr5:88551768). Genes in the sense and antisense directions are shown in red and blue, respectively. The location of each SNP is depicted by a vertical gray line. Gray arcs indicate predicted target genes for the scATAC peak containing the SNP of interest.

With our single-cell multiome, we sought to better understand risk loci identified by GWASs of complex eye disorders. To this end, we compiled a list of 1,331 unique index SNPs from the NHGRI-EBI GWAS Catalog representing GWAS hits for five eye diseases: AMD, glaucoma, DR, myopia, and MacTel (Figure 4A; Table S3).^25^ The vast majority (96.5%) of these SNPs localized to noncoding regions of the genome and, thus, could not be interpreted with scRNA-seq data alone. We performed linkage disequilibrium (LD) expansion on all index SNPs to include nearby variants with high probability of coinheritance (LD R^2^ > 0.9 based on phase 3 genotypes from the 1000 Genomes Project) (Figure S5B).^26^ From this, we obtained a total of 7,100 SNPs in loci associated with complex eye disorders, 7,034 (99.1%) of which were noncoding.

We first examined the small number of LD expanded SNPs that encoded an amino acid change or premature stop codon (Data S5). Using our scRNA-seq data, we found that coding variants implicated in AMD and glaucoma were most strongly expressed in microglia and Müller glia, respectively (Figure S6A). As a comparison, we also assessed the cell type expression of mutations known to cause monogenic retinal diseases based on gene annotations from the Retinal Information Network (Figure S6B).^27^ Consistent with expectations, genes for primary photoreceptor disorders, such as cone-rod dystrophy and retinitis pigmentosa, were most highly expressed in rods and cones, whereas disease genes for optic atrophy, a condition hallmarked by degeneration of retinal ganglion cells, showed the greatest expression in this cell type.

We next turned to the 7,034 unique SNPs after LD expansion that localized to the noncoding genome (Data S6). To determine in which retinal cell types these SNPs might be active, we overlapped SNP locations with scATAC peaks from our dataset. We found that 1,152 noncoding SNPs (16.4%) overlapped with a scATAC peak (Figure 4B, S5C, and S5D) and that most SNP-containing peaks were present in only one or two cell types (Figure S5E). We then conducted two orthogonal analyses to refine our list of SNPs for those more likely to possess gene regulatory functions. First we identified SNPs in scATAC peaks that were co-accessible with peaks in promoter regions, reasoning that this would select for SNPs in active enhancers. We detected 39,552 such promoter peaks in the human retina, 58.3% of which were co-accessible with at least one scATAC peak (Figure 4C). Leveraging our paired scRNA- and scATAC-seq data, we also searched for SNPs in peaks whose accessibility correlated with the expression of a nearby gene and termed genes that met this criterion ‘‘predicted target genes.’’ Using this method, we predicted target genes for 199,055 (32.1%) of the 620,386 scATAC peaks in our dataset (Figures 4D and S5F). For nearly half (44.3%) of these peaks, our predictions differed from the nearest gene on the linear genome (Figure S5G), suggesting that noncoding SNPs do not necessarily regulate their nearest gene.

We identified 241 SNPs in scATAC peaks that were co-accessible with promoter peaks and 374 SNPs that had predicted target genes, with 202 SNPs meeting both criteria. As an example, we examined rs4821699 residing in an intron of *TRIOBP* on chromosome 22. This locus has been implicated in glaucoma by multiple GWASs and encodes a protein thought to regulate cytoskeletal organization.^2,28,29^ We observed that rs4821699 was most accessible in retinal ganglion cells (Figure 4E), the major cell type that undergoes degeneration during glaucoma. Based on correlations with gene expression, the peak containing this SNP was predicted to target *TRIOBP*. We hypothesize that rs4821699 might therefore play a role in glaucoma by altering *TRIOBP* expression in retinal ganglion cells.

A handful of SNPs associated with eye diseases have been studied experimentally using retinal organoids derived from induced pluripotent stem cells. One such SNP is rs17421627, an index SNP from GWASs of MacTel, representing a T-to-G substitution on chromosome 5.^5,30^ We determined rs17421627 to be one of only five SNPs for MacTel with a predicted target gene and found the SNP to be most accessible in Müller glia and astrocytes (Figure 4E). Using linked gene expression data, we also predicted rs17421627 to act on LINC00461, a long noncoding RNA. Consistent with these predictions, deletion of the locus containing rs17421627 in human retinal organoids has been shown to significantly downregulate LINC00461, with the strongest effect in Müller glia.^31^ These examples illustrate how single-cell multiomics can reveal the cellular targets of noncoding variants in the retina and show how they might contribute to eye disorders.

### Integration of the single-cell multiome with HiChIP and eQTL data validates SNP-target gene predictions

**Figure 5.**
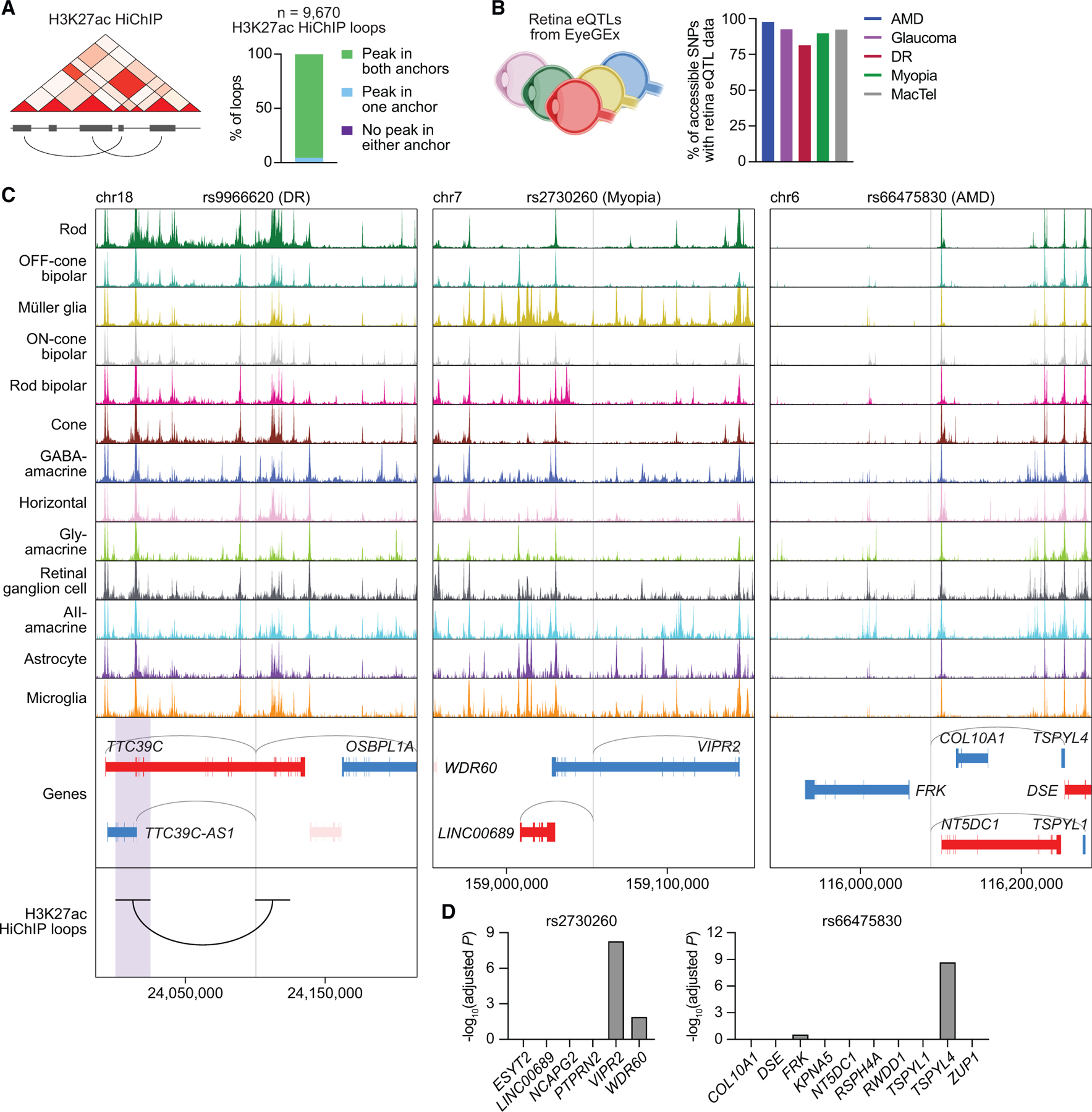
Integration of the single-cell multiome with HiChIP and eQTL data prioritizes functional noncoding polymorphisms in the human retina (A) Overlap of H3K27ac HiChIP loop anchors (n = 2 biological replicates) with scATAC peaks. (B) Percentage of SNPs in scATAC peaks for each disease with available retina eQTL data. (C) Sequencing tracks of chromatin accessibility near rs9966620 (chr18:24100771), rs2730260 (chr7:159054238), and rs66475830 (chr6:116087639). Genes in the sense and antisense directions are shown in red and blue, respectively. The location of each SNP is depicted by a vertical gray line. Gray arcs indicate predicted target genes for the scATAC peak containing the SNP of interest. The black arc overlapping with rs9966620 indicates a H3K27ac HiChIP loop with the region encompassed by the opposite anchor, highlighted in purple. (D) Significance of SNP-gene associations for rs2730260 or rs66475830 and their nearby genes, as determined by retina eQTL analysis. Adjusted p values for each gene were calculated by multiplying the nominal p value listed in the EyeGEx database by the number of SNP-gene pairs tested for that SNP.

To further prioritize our list of SNPs, we combined our data with two complementary methods for identifying functional SNP-gene interactions genome wide. We first performed HiChIP for acetylated histone H3 lysine 27 (H3K27ac), a mark of active enhancers and promoters,^32^ to characterize the three-dimensional (3D) enhancer ‘‘connectome’’ of the human retina (Figures S7A and S7B).^14,33^ We uncovered 16,692 loop anchors connected by 9,670 HiChIP loops, including several linking regions of chromatin accessibility to the transcription start sites (TSSs) of cell-type-specific genes (Figure S7C; Data S7). Of these loops, more than 95% overlapped with a scATAC peak in both anchors, and more than 99% overlapped with a peak in at least one anchor (Figure 5A). This result shows that accessible chromatin sites identified in scATAC-seq data possess biochemical characteristics of active enhancers and supports their connection to target genes. We also analyzed our list of SNPs using published human retina eQTL data from the Eye Genotype Expression (EyeGEx) database.^15^ For more than 90% of SNPs in scATAC peaks, retina eQTL data were available (Figure 5B), enabling genes whose mRNA expression in the human retina changed with specific SNPs to be identified at the bulk tissue level.

We found 187 disease-associated SNPs in scATAC peaks that were linked to a gene by a H3K27ac HiChIP loop. These included rs9966620, the top SNP from a GWAS of DR representing a G-to-A transition in an intron of *TTC39C* on chromosome 18.^34^ Using our multiome, we determined that the scATAC peak containing rs9966620 was most accessible in rods (Figure 5C). However, this peak also correlated with the expression of multiple target genes, hampering efforts to interpret how the SNP might function. Incorporating our HiChIP data, we were able to locate a 3D loop connecting rs9966620 with a region 75 kb upstream. This region intersected the TSS of only one gene, *TTC39C-AS1*, suggesting that rs9966620 may modulate DR risk by interacting with *TTC39C-AS1* in rods.

We also detected 596 disease-associated SNPs in scATAC peaks that were significantly associated with a gene by eQTL analysis. One example is rs2730260, an SNP in an intron of *VIPR2* that has been implicated in myopia.^35^ This locus encodes one of two known receptors for vasoactive intestinal peptide (VIP), a signaling molecule involved in visual processing.^36^ We found that rs2730260 resided in a chromatin accessibility peak specific to Müller glia that again had multiple predicted target genes (Figure 5C). This ambiguity was clarified by retina eQTL data, which showed that variation at rs2730260 significantly correlated with the expression of only *VIPR2* (Figure 5D), supporting this gene as the SNP’s primary target. Integration of eQTL data similarly improved our interpretation of rs66475830 on chromosome 6 in the *FRK-NT5DC1-COL10A1* risk locus for AMD.^37,38^ This region contains nearly 20 genes within a span of a megabase, making it particularly difficult to functionally annotate GWAS hits. From our single-cell data, we determined rs66475830 to be accessible in amacrine and horizontal cells and predicted *TSPYL1* and *TSPYL4* as target genes (Figure 5C). Retina eQTL analysis revealed that variation at this position was significantly associated with *TSPYL4* expression but not that of other nearby genes (Figure 5D), nominating *TSPYL4* as the effector gene of rs66475830.

Last, we identified many SNP-target gene relationships supported by HiChIP and eQTL data, such as rs77272443 and rs4102217 located in risk loci for myopia and glaucoma, respectively.^39,40^ For both of these SNPs, HiChIP and eQTL analyses again refined target gene predictions (Figures S8A and S8B), demonstrating how the combination of single-cell multiomics with other assays can enhance interpretation of noncoding variants in eye disease.

### Integration of the single-cell multiome with base-resolution deep learning nominates functional mechanisms for disease-associated SNPs

**Figure 6.**
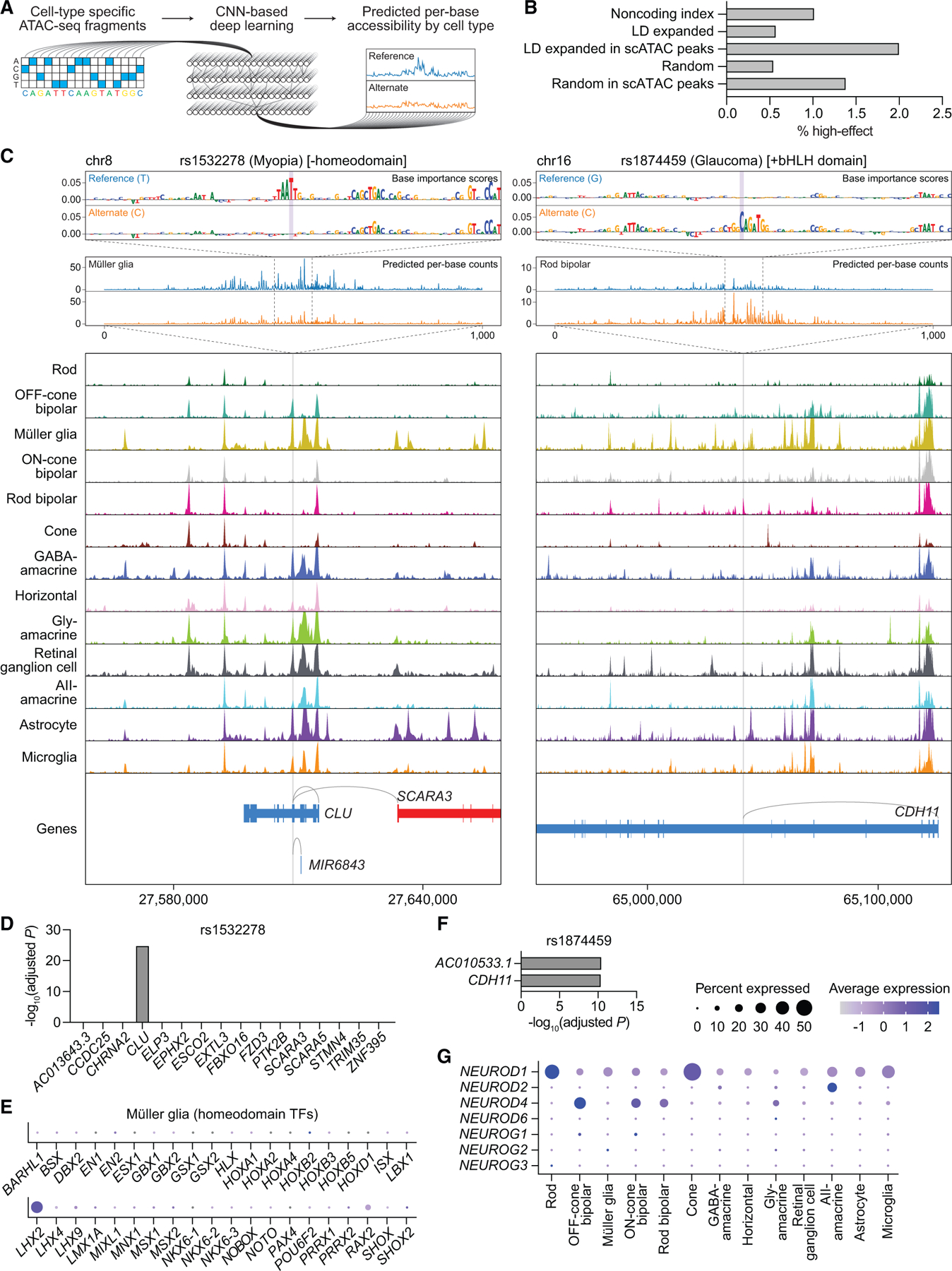
Integration of the single-cell multiome with base-resolution deep learning nominates functional mechanisms for disease-associated SNPs (A) Schematic of the CNN-based deep learning pipeline. (B) Percentage of noncoding index SNPs (n = 1,284), LD expanded SNPs (n = 7,034), LD expanded SNPs in scATAC peaks (n = 1,152), randomly selected GC-matched SNPs (n = 9,984), and randomly selected SNPs in scATAC peaks (n = 1,160) that were categorized as high-effect. (C) Top: predicted per-base accessibility for rs1532278 (chr8:27608798) and rs1874459 (chr16:65041801) in Müller glia and rod bipolar cells, respectively, as determined by deep learning models. A 100-bp window depicts the importance of each base to predicted accessibility at the SNP, and a 1,000-bp window depicts predicted per-base counts for the reference (blue) and alternate (orange) alleles. SNP bases are highlighted in purple. For rs1874459, similar changes in accessibility were predicted for OFF-cone bipolar, ON-cone bipolar, gly-amacrine, and AII-amacrine cells. Bottom: sequencing tracks of chromatin accessibility near rs1532278 and rs1874459. Genes in the sense and antisense directions are shown in red and blue, respectively. The location of each SNP is depicted by a vertical gray line. Gray arcs indicate predicted target genes for the scATAC peak containing the SNP of interest. (D and F) Significance of SNP-gene associations for rs1532278 (D) or rs1874459 (F) and their nearby genes, as determined by retina eQTL analysis. Adjusted p values for each gene were calculated by multiplying the nominal p value listed in the EyeGEx database by the number of SNP-gene pairs tested for that SNP. (E) Dot plot visualizing the normalized RNA expression of 40 different homeodomain TFs in Müller glia. The selected TFs correspond to the 40 homeodomain factors whose binding motifs were most significantly enriched in Müller glia, as determined by motif analysis (Data S4). (G) Dot plot visualizing the normalized RNA expression of neuroD and neurogenin family members by cell type.

CNN-based deep learning models have proven capable of discerning disease-associated SNPs from other noncoding variants.^8,10,11^ As a final method to prioritize SNPs in our dataset, we therefore trained CNNs derived from the BPNet architecture on scATAC-seq profiles for each of the 13 retinal cell types (Figure 6A, S9A, and S9B).^12^ At each SNP region, we compared the projected per-base change in chromatin accessibility between reference and alternate alleles using models specific to the different cell types. These calculations allowed us to identify ‘‘high-effect’’ SNPs, which we defined as SNPs predicted to cause a statistically significant (false discovery rate < 0.01) absolute log2 fold change of allele-specific read counts of greater than 0.5 in local chromatin accessibility in any cell type.

We found 23 SNPs (2.0%) residing in scATAC peaks that qualified as high-effect, a greater percentage than among index SNPs, LD expanded SNPs, random SNPs matched for GC content, and random SNPs residing in scATAC peaks (Figure 6B; Data S8). One of the top-scoring SNPs was rs1532278, an index SNP associated with myopia and residing in an intron of *CLU* on chromosome 8.^3^ Our atlas predicted rs1532278 to regulate *CLU*, a notion reinforced by eQTL data, and determined the SNP to be accessible in nine of 13 retinal cell types (Figures 6C and 6D). Despite this, base-resolution models projected a T-to-C transition at rs1532278 to alter chromatin accessibility only in Müller glia, specifically by disrupting the motif of a homeodomain TF. Our findings suggest that even though rs1532278 is accessible across multiple cell types, its functional effect in the retina might be restricted to Müller glia because of a cell-type-specific homeodomain TF. We speculate that this TF could be LHX2, given its robust expression in Müller glia by our scRNA-seq data (Figure 6E) as well as data from animal models.^41^

Another high-effect SNP was rs1874459 located in an intron of *CDH11* on chromosome 16, a locus implicated by multiple GWASs for glaucoma.^2,28^ Using our multiome, we found rs1874459 to be most accessible in rod bipolar cells and predicted *CDH11* as one of its target genes, an idea supported by eQTL data (Figures 6C and 6F). Incorporating base-resolution models, we then determined that the G-to-C transversion represented by rs1874459 introduced a new basic-helix-loop-helix (bHLH) domain, which was expected to increase accessibility in rod bipolar, OFF-cone bipolar, ON-cone bipolar, gly-amacrine, and AII-amacrine cells. Of the bHLH TFs, members of the neuroD and neurogenin families in particular were predicted by motif analysis to be significantly enriched in these five cell types (Figure 3A; Data S4). We thus compared all neuroD and neurogenin family members using our scRNA-seq data, which revealed only NEUROD4 to be specific to bipolar and amacrine cells (Figure 6G), consistent with its role in specifying these cell types during development.^42,43^ Our results suggest that rs1874459 may act on *CDH11* in bipolar and amacrine cells by creating a new bHLH domain recognized by NEUROD4.

## DISCUSSION

In this study, we applied single-cell multiomics, HiChIP, eQTL analysis, and base-resolution deep learning to the human retina to decipher the role of noncoding risk variants in five eye diseases. Integrating these methods allowed us to predict gene and cellular targets in the retina for hundreds of SNPs and nominate dozens as potentially pathogenic and meriting functional validation. From an initial list of more than 7,000 noncoding SNPs, we identified 1,152 located in chromatin accessibility peaks. We subsequently focused on SNPs (1) that were co-accessible with a promoter, (2) whose accessibility correlated with the expression of a nearby gene, (3) that were linked to a gene in 3D space by a H3K27ac HiChIP loop, (4) that demonstrated significant association with a gene based on retina eQTL data, and (5) that were predicted to alter local chromatin accessibility as determined by base-resolution models. We propose that SNPs meeting most or all of these criteria (Figure S10; Data S6) be prioritized in future validation efforts.

Our findings build upon recent studies that used primarily fetal tissue and stem-cell-derived organoids to map cell-type-specific chromatin accessibility in the human retina.^31,44^ Datasets from these studies are a rich resource for decoding retinal development and understanding congenital eye diseases. However, they might not fully recapitulate the biology of the mature retina, making them potentially less suitable for studying vision disorders that present later in life. Here we generated a single-cell multiome that complements prior datasets by pinpointing cellular targets for disease-associated SNPs in the adult human retina. We combined this multiome with multiple orthogonal analyses to define putative SNP-target gene interactions. By performing base-resolution deep learning, we were able to uncover insights not readily apparent from single-cell, HiChIP, and eQTL data, such as the predicted effect of SNPs on TF binding and the directionality of these effects. To facilitate its use, our atlas is publicly available at https://eyemultiome.su.domains/.

### Limitations of the study

Limitations of this study include the relatively low number of cells profiled from rarer cell types like microglia and retinal ganglion cells. Expansion of the current atlas with more cells and donors would be helpful to resolve functionally distinct cell subtypes and could reveal subtype-specific regions of chromatin accessibility that were overlooked here. During SNP prioritization, use of HiChIP and eQTL data from bulk retina may have also favored SNPs from more abundant cell types. Limited overlap of GWAS hits and significant eQTL because of systemic differences in study design may have led to lower concordance among our prioritization criteria.^45^

Finally, it should be noted that the majority of SNPs we examined did not overlap with any chromatin accessibility peaks, suggesting that they were not active in the retina. We hypothesize that many of these unassigned SNPs may instead function in other parts of the eye and thus could not be captured by our analysis. For instance, although the neural retina is damaged in AMD and DR, the retinal pigment epithelium and vasculature, respectively, are thought to be the primary sites of pathology.^46,47^ In glaucoma, the trabecular meshwork and ciliary body can modulate disease severity as evidenced by treatments that act on these tissues.^48^ Likewise, the choroid and sclera may be involved in myopia, given that they elongate alongside the retina with increasing nearsightedness.^49^ Multiomics characterization of these additional ocular regions would enable a more complete understanding of how noncoding SNPs contribute to vision disorders.

## STAR★METHODS

### RESOURCE AVAILABILITY

#### Lead contact

Further information and requests for resources and reagents should be directed to and will be fulfilled by the lead contact, Dr. Howard Y. Chang (howchang@stanford.edu).

#### Materials availability

Due to the limiting nature of primary samples, human tissues used in this study are not available upon request. This study did not involve any other unique materials.

#### Data and code availability

Raw and processed scRNA-seq, scATAC-seq, and HiChIP data from this study have been uploaded to Gene Expression Omnibus under the accession number GSE196235. A web page summarizing these data is also available at https://eyemultiome.su.domains/. Code used for scRNA- and scATAC-seq analysis is available at https://doi.org/10.5281/zenodo.6795162. Code used for BPNet model training is available at http://doi.org/10.5281/zenodo.6796067. BPNet models are available at https://doi.org/10.5281/zenodo.6330053.

### EXPERIMENTAL MODEL AND SUBJECT DETAILS

Postmortem adult human retinas were procured from consented donors by Lions VisionGift (Portland, OR, USA) or Lions Gift of Sight (St Paul, MN, USA) under protocols approved by the Eye Bank Association of America. None of the donors had a history of ocular disease. De-identified retinas were flash-frozen in liquid nitrogen with a maximum death-to-preservation interval of 12 hours and shipped to Stanford University for processing. Donor information is listed in Table S1.

### METHOD DETAILS

#### Nuclei isolation

Nuclei were isolated from frozen retinas using the Omni-ATAC protocol (https://doi.org/10.17504/protocols.io.6t8herw).^50^ Briefly, tissues were Dounce homogenized in cold homogenization buffer containing 0.3% IGEPAL CA-630 in the presence of protease and RNase inhibitors to release nuclei from frozen cells. Nuclei were subsequently purified via iodixanol gradient centrifugation and washed with ATAC resuspension buffer containing RNase inhibitor and 0.1% Tween-20 before permeabilization following the 10x Genomics demonstrated protocol for complex tissues (CG000375, Rev. B). After resuspension in diluted nuclei buffer, nuclei were counted using a manual hemocytometer to achieve a targeted nuclei recovery of 10,000 nuclei per sample.

#### scRNA- and scATAC-seq library generation

Joint scRNA- and scATAC-seq libraries were prepared using the 10x Genomics Single Cell Multiome ATAC + Gene Expression kit according to manufacturer’s instructions. Libraries were sequenced with paired-end 150-bp reads on an Illumina NovaSeq 6000 to a target depth of 250 million read pairs per sample.

#### HiChIP library generation

H3K27ac HiChIP libraries were prepared as previously reported with minor modifications.^14^ Briefly, following isolation of nuclei from frozen retinas as described above, ~8 million nuclei from each sample were washed with nuclei isolation buffer from the diploid chromatin conformation capture (Dip-C) protocol and fixed with 2% paraformaldehyde at room temperature for 10 minutes.^51^ Fixed nuclei were then washed twice with cold 1% bovine serum albumin in phosphate-buffered saline before resuspension in 0.5% sodium dodecyl sulfate and resumption of the published HiChIP protocol. Digestion was performed using the MboI restriction enzyme, and sonication was conducted using a Covaris E220 with 5 duty cycles, peak incident power of 140, and 200 cycles per burst for 4 minutes. The ab4729 ChIP validated antibody from Abcam was used to target H3K27ac. HiChIP libraries were sequenced with paired-end 75-bp reads on either an Illumina HiSeq 400 or Illumina NextSeq 550.

### QUANTIFICATION AND STATISTICAL ANALYSIS

#### scRNA- and scATAC-seq data preprocessing and quality control

Demultiplexed scRNA- and scATAC-seq fastq files were inputted into the Cell Ranger ARC pipeline (version 2.0.0) from 10x Genomics to generate barcoded count matrices of gene expression and ATAC data. For each sample, count matrices were loaded in ArchR and selected for barcodes that appeared in both the scRNA-seq and scATAC-seq datasets.^52^ Samples in ArchR were quality control filtered for nuclei with 200–50,000 RNA transcripts, <1% mitochondrial reads, <5% ribosomal reads, TSS enrichment >6, and >2,500 ATAC fragments. Quality control filtered nuclei subsequently underwent automated removal of doublets using the filter-Doublets function in ArchR, which identifies and removes the nearest neighbors of simulated doublets.^52^

#### scRNA-seq data analysis

scRNA-seq data from nuclei remaining after quality control filtering and automated removal of doublets were analyzed using Seurat (version 3.1.5).^53^ After merging all preprocessed samples into a single Seurat object, gene expression counts were normalized using the NormalizeData function, scaled using the ScaleData function, and batch corrected using Harmony.^54^ Graph-based clustering was then performed on the Harmony-corrected data using the top 20 principal components at a resolution of 0.5. Cluster identities were manually annotated based on the expression of genes from published scRNA-seq studies of the human retina.^16–18^ Marker genes for each cluster were additionally identified using the FindAllMarkers function with a minimum fraction of 0.5 and a log2 fold change of 1 (Data S1). Clusters expressing canonical marker genes from different cell types were designated as putative doublets and excluded, after which re-clustering was performed using the same parameters. Clusters with no detected marker genes were also excluded, after which the dataset was also re-clustered. Clusters in the final dataset representing subpopulations of the same cell type were grouped together for downstream analyses.

#### scATAC-seq data analysis

scATAC-seq data were analyzed using ArchR (version 1.0.1) based on barcoded cell type identities from scRNA-seq.^52^ For each cell type, pseudo-bulk ATAC replicates were created using the addGroupCoverages function with default parameters, which generated between two to five replicates depending on how many cells of that type were present in each sample. Chromatin accessibility peaks on chromosomes 1–22 and X and outside of blacklist regions were then called using the addReproduciblePeakSet function and MACS2,^55,56^ with scATAC peaks for each cell type defined as those present in at least two pseudo-bulk ATAC replicates (Data S2). Marker peaks were identified using the getMarkerFeatures function with a log2 fold change ≥1 and false discovery rate ≥ 0.01 as determined by Wilcoxon pairwise comparisons (Data S3). Promoter peaks were defined as scATAC peaks within 2,000 bp upstream or 100 bp downstream of a TSS, and peaks co-accessible to promoter peaks were identified using the getCoAccessibility function with a correlation cutoff of 0.3 and resolution of 1. Predicted target genes for each scATAC peak were generated using the getPeak2GeneLinks function integrating barcode-matched RNA expression data from scRNA-seq with a correlation cutoff of 0.3 and resolution of 1. Nearest genes were determined using the BEDTools closest function based on gene annotations from TxDb.Hsapiens.UCSC.hg38.knownGene.^57^

#### Bulk ATAC-seq data analysis

Bulk ATAC-seq analysis was performed on published ATAC-seq data from five healthy human retinas.^20^ After adapter trimming, fastq files were mapped to the hg38 genome using Bowtie2 and filtered to remove PCR duplicates and retain reads from only chromosomes 1–22 and X.^58^ Peak calling was then conducted individually on each sample using MACS2,^55^ followed by exclusion of peaks in blacklist regions.^56^ Peak calls present in at least two of the five retinas were included in the bulk ATAC-seq peak set.

#### Sequencing tracks

Sequencing tracks of chromatin accessibility were generated in ArchR using the plotBrowserTrack function and were normalized by the total number of reads in TSS regions.^52^ In some cases, bigWig files generated in ArchR using the getGroupBW function were visualized on the WashU Epigenome Browser.^59^ All data were aligned and annotated to the hg38 reference genome unless otherwise stated.

#### Motif enrichment analysis

TF motif enrichment analysis was performed on scATAC peaks using the peakAnnoEnrichment function in ArchR with default parameters based on position frequency matrices from Cis-BP (Data S4).^52,60^ Footprinting analysis of TFs was conducted using the get-Footprints function in ArchR.^52^ To correct for Tn5 insertion bias, the Tn5 insertion signal was subtracted from footprinting signals prior to plotting.

#### SNP selection and LD expansion

Index SNPs implicated in AMD, glaucoma, DR, myopia, or MacTel and located on chromosomes 1–22 and X were collected from the NHGRI-EBI GWAS Catalog, a curated collection of human GWAS.^25^ LD expansion was then performed using LDlinkR to add any SNPs in LD with each index SNP,^61^ defined as a LD *R*^2^ value >0.9 in the phase 3 genotypes of the 1000 Genomes Project.^26^ LD expanded SNPs were filtered to exclude variants in coding regions based on annotations in dbSNP to obtain the final set of noncoding SNPs (Data S6).^62^ A list of all GWAS used in this study is provided in Table S3.

#### HiChIP data analysis

HiChIP sequencing files were initially processed using the HiC-Pro pipeline (version 2.11.0) to remove duplicate reads, assign reads to MboI restriction fragments, filter for valid interactions, and generate binned interaction matrices.^63^ Filtered read pairs from HiC-Pro were subsequently converted into .hic files and inputted into HiCCUPS from the Juicer pipeline to call loops (Data S7).^64^ HiChIP interaction maps depicting all valid interactions identified by HiC-Pro were visualized using Juicebox.^65^

#### eQTL analysis

Retina eQTL data were obtained from the Eye Genotype Expression (EyeGEx) database.^15^ Each of the 1,152 SNPs overlapping with a scATAC peak was searched in the database and the nominal p value of any gene associations with that SNP noted. Adjusted p values were calculated by multiplying the nominal p value by the number of SNP-gene pairs tested for that SNP. Interactions with an adjusted p value < 0.05 were considered significant.

#### Deep learning model training

scATAC-seq reads from the Cell Ranger ARC pipeline were aggregated by cell type to generate cell type-specific fragments files. The fragments files were converted to BigWig tracks of base-resolution Tn5 insertion sites with an +4/−4 shift to account for Tn5 shift. For each cell type, in addition to the peak regions, we selected an equal number of non-peak regions that were matched for GC content in their peaks. We then trained cell type-specific BPNet models to predict the log counts and base-resolution Tn5 insertion profiles as previously reported.^8,12^ Briefly, the BPNet model takes as input a 2,114 bp one-hot encoded input sequence and predicts the ATAC-seq profile and log counts in a 1,000 bp window centered at the input sequence. Following BPNet formulation, we used a multinomial negative log likelihood (MNLL) for the profile output of the model and a mean square error (MSE) loss for the log counts output of the model. The relative loss weight used for the counts loss was 0.1 times the mean total counts per region. During each epoch, training examples were jittered by up to 500 bp on either side and a random half of the sequences were reverse complemented. Each batch contained a 10:1 ratio of peaks to non-peak regions. Five models were trained for each cell type corresponding to five disjoint training folds. Model training was performed using Keras/Tensorflow 2.

#### SNP scoring with BPNet

To score LD expanded SNPs associated with eye disease, we centered the input window at the SNP and obtained the log2 fold change in predicted counts between the reference and alternate alleles for each cell type-specific model. We averaged the log2 fold change over the five model folds for each SNP and cell type. To obtain p values, we performed one-sided Poisson tests of the predicted alternate allele count with the rate parameter set to the predicted reference allele count (counts averaged over five folds). For each SNP, we combined p values across cell types with Fisher’s method and performed Benjamini-Hochberg correction. SNPs with an absolute fold-averaged log2 fold change >0.5 and false discovery rate <0.01 were assigned putative ‘‘high effect’’ annotation. To obtain a background set, random noncoding SNPs were chosen by shuffling a list of all SNPs from the 1000 Genomes Project,^26^ filtering out coding regions, and selecting the first 10,000 entries. Only random SNPs localized to chromosomes 1–22 and X were then retained, leaving 9,984 background SNPs. Background SNPs had similar GC content as disease-associated LD expanded SNPs (51% versus 52%) and were scored as described above. Base importance tracks were visualized using Logomaker.^66^

## Supplementary Material

1

2

3

4

5

6

7

8

9

10

## Figures and Tables

**Table:** KEY RESOURCES TABLE

REAGENT or RESOURCE	SOURCE	IDENTIFIER
Antibodies		

Anti-histone H3 (acetyl K27) antibody	Abcam	Cat# ab4729; RRID: AB_2118291

Biological samples		

Frozen adult postmortem human retinas	Lions VisionGift, Lions Gift of Sight	N/A

Chemicals, peptides, and recombinant proteins		

IGEPAL CA-630	Sigma-Aldrich	Cat# I8896
RNase inhibitor	New England BioLabs	Cat# M0314
Protease inhibitor	Roche	Cat# 11697498001
Tween-20	Roche	Cat# 11332465001
Bovine serum albumin	Miltenyi Biotec	Cat# 130-091-376
Digitonin	Thermo Fischer Scientific	Cat# BN2006
Glycerol	Ricca Chemical	Cat# 3290-16
Paraformaldehyde	Electron Microscopy Sciences	Cat# 50-980-487
Sodium dodecyl sulfate	Invitrogen	Cat# 15553027
Triton X-100	Sigma-Aldrich	Cat# 93443
MboI	New England BioLabs	Cat# R0147
Biotin-14-dATP	Jena Bioscience	Cat# NU-835-BIO14
Klenow fragment	New England BioLabs	Cat# M0210
T4 DNA ligase	New England BioLabs	Cat# M0202
Dimethylformamide	Sigma-Aldrich	Cat# 227056
Sodium deoxycholate	Bioworld	Cat# 40430018

Critical commercial assays		

Single Cell Multiome ATAC + Gene Expression kit	10x Genomics	Cat# 1000285

Deposited data		

scRNA-seq, scATAC-seq, and HiChIP data	This paper	Gene Expression Omnibus: GSE196235
BPNet models	This paper	Zenodo: https://doi.org/10.5281/zenodo.6330053

Software and algorithms		

Cell Ranger-ARC	10x Genomics	v2.0.0
ArchR	Granja et al., 2021	v1.01
Seurat	Stuart et al., 2019	v3.1.5
Harmony	Korsunsky et al., 2019	v1.0
MACS2	Feng et al., 2012	v2.1.1
BEDTools	Quinlan et al., 2010	v2.30.0
LDlinkR	Myers et al., 2020	v1.2.0
HiC-Pro	Servant et al., 2015	v2.11.0
Juicer	Rao et al., 2014	v1.6
Juicebox	Durand et al., 2016	v1.11.08
Logomaker	Tareen et al., 2020	v0.8

Other		

Dounce homogenizers	Sigma-Aldrich	Cat# D8938
SPRIselect beads	Beckman Coulter	Cat# B23317
Protein A Dynabeads	Invitrogen	Cat# 10001D
Streptavidin Dynabeads	Invitrogen	Cat# 65001

## Highlights

- Single-cell RNA and chromatin profiling of human retina characterizes 13 cell types
- H3K27ac HiChIP of human retina identifies enhancer-promoter connections
- Deep learning predicts effects of single base changes on chromatin accessibility
- Integrative approach prioritizes noncoding risk variants in complex eye diseases

## References

[1] Fritsche LG, Igl W, Bailey JNC, Grassmann F, Sengupta S, Bragg-Gresham JL, Burdon KP, Hebbring SJ, Wen C, Gorski M, (2016). A large genome-wide association study of age-related macular degeneration highlights contributions of rare and common variants. Nat. Genet 48, 134–143. 26691988 10.1038/ng.3448 PMC4745342

[2] MacGregor S, Ong J-S, An J, Han X, Zhou T, Siggs OM, Law MH, Souzeau E, Sharma S, Lynn DJ, (2018). Genome-wide association study of intraocular pressure uncovers new pathways to glaucoma. Nat. Genet 50, 1067–1071. 30054594 10.1038/s41588-018-0176-y

[3] Tedja MS, Wojciechowski R, Hysi PG, Eriksson N, Furlotte NA, Verhoeven VJM, Iglesias AI, Meester-Smoor MA, Tompson SW, Fan Q, (2018). Genome-wide association meta-analysis highlights light-induced signaling as a driver for refractive error. Nat. Genet 50, 834–848. 29808027 10.1038/s41588-018-0127-7 PMC5980758

[4] Grassi MA, Tikhomirov A, Ramalingam S, Below JE, Cox NJ, and Nicolae DL (2011). Genome-wide meta-analysis for severe diabetic retinopathy. Hum. Mol. Genet 20, 2472–2481. 21441570 10.1093/hmg/ddr121 PMC3098732

[5] Scerri TS, Quaglieri A, Cai C, Zernant J, Matsunami N, Baird L, Scheppke L, Bonelli R, Yannuzzi LA, Friedlander M, (2017). Genome-wide analyses identify common variants associated with macular telangiectasia type 2. Nat. Genet 49, 559–567. 28250457 10.1038/ng.3799

[6] Cano-Gamez E, and Trynka G (2020). From GWAS to function: using functional Genomics to identify the mechanisms underlying complex diseases. Front. Genet 11, 424. 32477401 10.3389/fgene.2020.00424 PMC7237642

[7] Corces MR, Shcherbina A, Kundu S, Gloudemans MJ, Frésard L, Granja JM, Louie BH, Eulalio T, Shams S, Bagdatli ST, (2020). Single-cell epigenomic analyses implicate candidate causal variants at inherited risk loci for Alzheimer’s and Parkinson’s diseases. Nat. Genet 52, 1158–1168. 33106633 10.1038/s41588-020-00721-x PMC7606627

[8] Trevino AE, Müller F, Andersen J, Sundaram L, Kathiria A, Shcherbina A, Farh K, Chang HY, Pașca AM, Kundaje A, (2021). Chromatin and gene-regulatory dynamics of the developing human cerebral cortex at single-cell resolution. Cell 184, 5053–5069.e23. 34390642 10.1016/j.cell.2021.07.039

[9] King HW, Wells KL, Shipony Z, Kathiria AS, Wagar LE, Lareau C, Orban N, Capasso R, Davis MM, Steinmetz LM, (2021). Integrated single-cell transcriptomics and epigenomics reveals strong germinal center-associated etiology of autoimmune risk loci. Sci. Immunol 6, eabh3768. 34623901 10.1126/sciimmunol.abh3768 PMC8859880

[10] Zhou J, and Troyanskaya OG (2015). Predicting effects of noncoding variants with deep learning-based sequence model. Nat. Methods 12, 931–934. 26301843 10.1038/nmeth.3547 PMC4768299

[11] Kelley DR, Snoek J, and Rinn JL (2016). Basset: learning the regulatory code of the accessible genome with deep convolutional neural networks. Genome Res 26, 990–999. 27197224 10.1101/gr.200535.115 PMC4937568

[12] Avsec Ž, Weilert M, Shrikumar A, Krueger S, Alexandari A, Dalal K, Fropf R, McAnany C, Gagneur J, Kundaje A, and Zeitlinger J (2021). Base-resolution models of transcription-factor binding reveal soft motif syntax. Nat. Genet 53, 354–366. 33603233 10.1038/s41588-021-00782-6 PMC8812996

[13] Kim DS, Risca VI, Reynolds DL, Chappell J, Rubin AJ, Jung N, Donohue LKH, Lopez-Pajares V, Kathiria A, Shi M, (2021). The dynamic, combinatorial cis-regulatory lexicon of epidermal differentiation. Nat. Genet 53, 1564–1576. 34650237 10.1038/s41588-021-00947-3 PMC8763320

[14] Mumbach MR, Rubin AJ, Flynn RA, Dai C, Khavari PA, Greenleaf WJ, and Chang HY (2016). HiChIP: efficient and sensitive analysis of protein-directed genome architecture. Nat. Methods 13, 919–922. 27643841 10.1038/nmeth.3999 PMC5501173

[15] Ratnapriya R, Sosina OA, Starostik MR, Kwicklis M, Kapphahn RJ, Fritsche LG, Walton A, Arvanitis M, Gieser L, Pietraszkiewicz A, (2019). Retinal transcriptome and eQTL analyses identify genes associated with age-related macular degeneration. Nat. Genet 51, 606–610. 30742112 10.1038/s41588-019-0351-9 PMC6441365

[16] Menon M, Mohammadi S, Davila-Velderrain J, Goods BA, Cadwell TD, Xing Y, Stemmer-Rachamimov A, Shalek AK, Love JC, Kellis M, and Hafler BP (2019). Single-cell transcriptomic atlas of the human retina identifies cell types associated with age-related macular degeneration. Nat. Commun 10, 4902. 31653841 10.1038/s41467-019-12780-8 PMC6814749

[17] Lukowski SW, Lo CY, Sharov AA, Nguyen Q, Fang L, Hung SS, Zhu L, Zhang T, Grünert U, Nguyen T, (2019). A single-cell transcriptome atlas of the adult human retina. EMBO J 38, e100811. 31436334 10.15252/embj.2018100811 PMC6745503

[18] Yan W, Peng Y-R, van Zyl T, Regev A, Shekhar K, Juric D, and Sanes JR (2020). Cell atlas of the human fovea and peripheral retina. Sci. Rep 10, 9802. 32555229 10.1038/s41598-020-66092-9 PMC7299956

[19] Lyu Y, Zauhar R, Dana N, Strang CE, Hu J, Wang K, Liu S, Pan N, Gamlin P, Kimble JA, (2021). Implication of specific retinal cell-type involvement and gene expression changes in AMD progression using integrative analysis of single-cell and bulk RNA-seq profiling. Sci. Rep 11, 15612. 34341398 10.1038/s41598-021-95122-3 PMC8329233

[20] Wang J, Zibetti C, Shang P, Sripathi SR, Zhang P, Cano M, Hoang T, Xia S, Ji H, Merbs SL, (2018). ATAC-Seq analysis reveals a widespread decrease of chromatin accessibility in age-related macular degeneration. Nat. Commun 9, 1364. 29636475 10.1038/s41467-018-03856-y PMC5893535

[21] Wang S, Sengel C, Emerson MM, and Cepko CL (2014). A gene regulatory network controls the binary fate decision of rod and bipolar cells in the vertebrate retina. Dev. Cell 30, 513–527. 25155555 10.1016/j.devcel.2014.07.018 PMC4304698

[22] Wu F, Li R, Umino Y, Kaczynski TJ, Sapkota D, Li S, Xiang M, Fliesler SJ, Sherry DM, Gannon M, (2013). Onecut1 is essential for horizontal cell genesis and retinal integrity. J. Neurosci 33, 13053–13065. 23926259 10.1523/JNEUROSCI.0116-13.2013 PMC3735885

[23] Gan L, Xiang M, Zhou L, Wagner DS, Klein WH, and Nathans J (1996). POU domain factor Brn-3b is required for the development of a large set of retinal ganglion cells. Proc. Natl. Acad. Sci. USA 93, 3920–3925. 8632990 10.1073/pnas.93.9.3920 PMC39460

[24] Smith AM, Gibbons HM, Oldfield RL, Bergin PM, Mee EW, Faull RLM, and Dragunow M (2013). The transcription factor PU.1 is critical for viability and function of human brain microglia. Glia 61, 929–942. 23483680 10.1002/glia.22486

[25] Buniello A, MacArthur JAL, Cerezo M, Harris LW, Hayhurst J, Malangone C, McMahon A, Morales J, Mountjoy E, Sollis E, (2019). The NHGRI-EBI GWAS Catalog of published genome-wide association studies, targeted arrays and summary statistics 2019. Nucleic Acids Res 47, D1005–D1012. 30445434 10.1093/nar/gky1120 PMC6323933

[26] 1000 Genomes Project Consortium; Abecasis GR, Auton A, Brooks LD, DePristo MA, Durbin RM, Handsaker RE, Kang HM, Marth GT, and McVean GA (2012). An integrated map of genetic variation from 1, 092 human genomes. Nature 491, 56–65. 23128226 10.1038/nature11632 PMC3498066

[27] Daiger SP, Sullivan LS, and Bowne SJ (2013). Genes and mutations causing retinitis pigmentosa. Clin. Genet 84, 132–141. 23701314 10.1111/cge.12203 PMC3856531

[28] Gharahkhani P, Jorgenson E, Hysi P, Khawaja AP, Pendergrass S, Han X, Ong JS, Hewitt AW, Segrè AV, Rouhana JM, (2021). Genome-wide meta-analysis identifies 127 open-angle glaucoma loci with consistent effect across ancestries. Nat. Commun 12, 1258. 33627673 10.1038/s41467-020-20851-4 PMC7904932

[29] Shahin H, Walsh T, Sobe T, Abu Sa’ed J, Abu Rayan A, Lynch ED, Lee MK, Avraham KB, King M-C, and Kanaan M (2006). Mutations in a novel isoform of TRIOBP that encodes a filamentous-actin binding protein are responsible for DFNB28 recessive nonsyndromic hearing loss. Am. J. Hum. Genet 78, 144–152. 16385458 10.1086/499495 PMC1380212

[30] Bonelli R, Jackson VE, Prasad A, Munro JE, Farashi S, Heeren TFC, Pontikos N, Scheppke L, Friedlander M, MacTel Consortium, (2021). Identification of genetic factors influencing metabolic dysregulation and retinal support for MacTel, a retinal disorder. Commun. Biol 4, 473. 33837246 10.1038/s42003-021-01972-y PMC8035146

[31] Thomas ED, Timms AE, Giles S, Harkins-Perry S, Lyu P, Hoang T, Qian J, Jackson VE, Bahlo M, Blackshaw S, (2022). Cell-specific cis-regulatory elements and mechanisms of non-coding genetic disease in human retina and retinal organoids. Dev. Cell 57, 820–836.e6. 35303433 10.1016/j.devcel.2022.02.018 PMC9126240

[32] Creyghton MP, Cheng AW, Welstead GG, Kooistra T, Carey BW, Steine EJ, Hanna J, Lodato MA, Frampton GM, Sharp PA, (2010). Histone H3K27ac separates active from poised enhancers and predicts developmental state. Proc. Natl. Acad. Sci. USA 107, 21931–21936. 21106759 10.1073/pnas.1016071107 PMC3003124

[33] Mumbach MR, Satpathy AT, Boyle EA, Dai C, Gowen BG, Cho SW, Nguyen ML, Rubin AJ, Granja JM, Kazane KR, (2017). Enhancer connectome in primary human cells identifies target genes of disease-associated DNA elements. Nat. Genet 49, 1602–1612. 28945252 10.1038/ng.3963 PMC5805393

[34] Meng W, Chan BW, Ezeonwumelu C, Hébert, H.L., Campbell, A., Soler, V., and Palmer, C.N. (2019). A genome-wide association study implicates that the TTC39C gene is associated with diabetic maculopathy with decreased visual acuity. Ophthalmic Genet 40, 252–258. 31264924 10.1080/13816810.2019.1633549

[35] Shi Y, Gong B, Chen L, Zuo X, Liu X, Tam POS, Zhou X, Zhao P, Lu F, Qu J, (2013). A genome-wide meta-analysis identifies two novel loci associated with high myopia in the Han Chinese population. Hum. Mol. Genet 22, 2325–2333. 23406873 10.1093/hmg/ddt066

[36] Park SJH, Borghuis BG, Rahmani P, Zeng Q, Kim I-J, and Demb JB (2015). Function and circuitry of VIP+ interneurons in the mouse retina. J. Neurosci 35, 10685–10700. 26224854 10.1523/JNEUROSCI.0222-15.2015 PMC4518048

[37] Yu Y, Bhangale TR, Fagerness J, Ripke S, Thorleifsson G, Tan PL, Souied EH, Richardson AJ, Merriam JE, Buitendijk GHS, (2011). Common variants near FRK/COL10A1 and VEGFA are associated with advanced age-related macular degeneration. Hum. Mol. Genet 20, 3699–3709. 21665990 10.1093/hmg/ddr270 PMC3159552

[38] Fritsche LG, Chen W, Schu M, Yaspan BL, Yu Y, Thorleifsson G, Zack DJ, Arakawa S, Cipriani V, Ripke S, (2013). Seven new loci associated with age-related macular degeneration. Nat. Genet 45, 433–439.e1–2. 23455636 10.1038/ng.2578 PMC3739472

[39] Tideman JWL, Pärssinen O, Haarman AEG, Khawaja AP, Wedenoja J, Williams KM, Biino G, Ding X, Kähönen M, Lehtimäki T, (2021). Evaluation of shared genetic susceptibility to high and low myopia and hyperopia. JAMA Ophthalmol 139, 601–609. 33830181 10.1001/jamaophthalmol.2021.0497 PMC8033508

[40] Craig JE, Han X, Qassim A, Hassall M, Cooke Bailey JN, Kinzy TG, Khawaja AP, An J, Marshall H, Gharahkhani P, (2020). Multitrait analysis of glaucoma identifies new risk loci and enables polygenic prediction of disease susceptibility and progression. Nat. Genet 52, 160–166. 31959993 10.1038/s41588-019-0556-y PMC8056672

[41] de Melo J, Miki K, Rattner A, Smallwood P, Zibetti C, Hirokawa K, Monuki ES, Campochiaro PA, and Blackshaw S (2012). Injury-independent induction of reactive gliosis in retina by loss of function of the LIM homeodomain transcription factor Lhx2. Proc. Natl. Acad. Sci. USA 109, 4657–4662. 22393024 10.1073/pnas.1107488109 PMC3311371

[42] Hatakeyama J, Tomita K, Inoue T, and Kageyama R (2001). Roles of homeobox and bHLH genes in specification of a retinal cell type. Development 128, 1313–1322. 11262232 10.1242/dev.128.8.1313

[43] Inoue T, Hojo M, Bessho Y, Tano Y, Lee JE, and Kageyama R (2002). Math3 and NeuroD regulate amacrine cell fate specification in the retina. Development 129, 831–842. 11861467 10.1242/dev.129.4.831

[44] Finkbeiner C, Ortuño-Lizarńn I, Sridhar A, Hooper M, Petter S, and Reh TA (2022). Single-cell ATAC-seq of fetal human retina and stem-cell-derived retinal organoids shows changing chromatin landscapes during cell fate acquisition. Cell Rep 38, 110294. 35081356 10.1016/j.celrep.2021.110294

[45] Mostafavi H, Spence JP, Naqvi S, and Pritchard JK (2022). Limited overlap of eQTLs and GWAS hits due to systematic differences in discovery. Preprint at bioRxiv 10.1101/2022.05.07.491045.

[46] Fleckenstein M, Keenan TDL, Guymer RH, Chakravarthy U, Schmitz-Valckenberg S, Klaver CC, Wong WT, and Chew EY (2021). Age-related macular degeneration. Nat. Rev. Dis. Prim 7, 31. 33958600 10.1038/s41572-021-00265-2 PMC12878645

[47] Duh EJ, Sun JK, and Stitt AW (2017). Diabetic retinopathy: current understanding, mechanisms, and treatment strategies. JCI Insight 2, 93751. 28724805 10.1172/jci.insight.93751 PMC5518557

[48] Weinreb RN, Aung T, and Medeiros FA (2014). The pathophysiology and treatment of glaucoma: a review. JAMA 311, 1901–1911. 24825645 10.1001/jama.2014.3192 PMC4523637

[49] Morgan IG, Ohno-Matsui K, and Saw S-M (2012). Myopia. Lancet 379, 1739–1748. 22559900 10.1016/S0140-6736(12)60272-4

[50] Corces MR, Trevino AE, Hamilton EG, Greenside PG, Sinnott-Armstrong NA, Vesuna S, Satpathy AT, Rubin AJ, Montine KS, Wu B, (2017). An improved ATAC-seq protocol reduces background and enables interrogation of frozen tissues. Nat. Methods 14, 959–962. 28846090 10.1038/nmeth.4396 PMC5623106

[51] Tan L (2021). Determining the 3D genome structure of a single mammalian cell with Dip-C. STAR Protoc 2, 100622. 34195675 10.1016/j.xpro.2021.100622 PMC8225968

[52] Granja JM, Corces MR, Pierce SE, Bagdatli ST, Choudhry H, Chang HY, and Greenleaf WJ (2021). ArchR is a scalable software package for integrative single-cell chromatin accessibility analysis. Nat. Genet 53, 403–411. 33633365 10.1038/s41588-021-00790-6 PMC8012210

[53] Stuart T, Butler A, Hoffman P, Hafemeister C, Papalexi E, Mauck WM, Hao Y, Stoeckius M, Smibert P, and Satija R (2019). Comprehensive integration of single-cell data. Cell 177, 1888–1902.e21. 31178118 10.1016/j.cell.2019.05.031 PMC6687398

[54] Korsunsky I, Millard N, Fan J, Slowikowski K, Zhang F, Wei K, Baglaenko Y, Brenner M, Loh P-R, and Raychaudhuri S (2019). Fast, sensitive and accurate integration of single-cell data with Harmony. Nat. Methods 16, 1289–1296. 31740819 10.1038/s41592-019-0619-0 PMC6884693

[55] Feng J, Liu T, Qin B, Zhang Y, and Liu XS (2012). Identifying ChIP-seq enrichment using MACS. Nat. Protoc 7, 1728–1740. 22936215 10.1038/nprot.2012.101 PMC3868217

[56] Amemiya HM, Kundaje A, and Boyle AP (2019). The ENCODE blacklist: identification of problematic regions of the genome. Sci. Rep 9, 9354. 31249361 10.1038/s41598-019-45839-z PMC6597582

[57] Quinlan AR, and Hall IM (2010). BEDTools: a flexible suite of utilities for comparing genomic features. Bioinformatics 26, 841–842. 20110278 10.1093/bioinformatics/btq033 PMC2832824

[58] Langmead B, and Salzberg SL (2012). Fast gapped-read alignment with Bowtie 2. Nat. Methods 9, 357–359. 22388286 10.1038/nmeth.1923 PMC3322381

[59] Li D, Hsu S, Purushotham D, Sears RL, and Wang T (2019). WashU Epigenome browser update 2019. Nucleic Acids Res 47, W158–W165. 31165883 10.1093/nar/gkz348 PMC6602459

[60] Weirauch MT, Yang A, Albu M, Cote AG, Montenegro-Montero A, Drewe P, Najafabadi HS, Lambert SA, Mann I, Cook K, (2014). Determination and inference of eukaryotic transcription factor sequence specificity. Cell 158, 1431–1443. 25215497 10.1016/j.cell.2014.08.009 PMC4163041

[61] Myers TA, Chanock SJ, and Machiela MJ (2020). LDlinkR: an R package for rapidly calculating linkage disequilibrium statistics in diverse populations. Front. Genet 11, 157. 32180801 10.3389/fgene.2020.00157 PMC7059597

[62] Sherry ST, Ward MH, Kholodov M, Baker J, Phan L, Smigielski EM, and Sirotkin K (2001). dbSNP: the NCBI database of genetic variation. Nucleic Acids Res 29, 308–311. 11125122 10.1093/nar/29.1.308 PMC29783

[63] Servant N, Varoquaux N, Lajoie BR, Viara E, Chen C-J, Vert J-P, Heard E, Dekker J, and Barillot E (2015). HiC-Pro: an optimized and flexible pipeline for Hi-C data processing. Genome Biol 16, 259. 26619908 10.1186/s13059-015-0831-x PMC4665391

[64] Rao SSP, Huntley MH, Durand NC, Stamenova EK, Bochkov ID, Robinson JT, Sanborn AL, Machol I, Omer AD, Lander ES, and Aiden EL (2014). A 3D map of the human genome at kilobase resolution reveals principles of chromatin looping. Cell 159, 1665–1680. 25497547 10.1016/j.cell.2014.11.021 PMC5635824

[65] Durand NC, Robinson JT, Shamim MS, Machol I, Mesirov JP, Lander ES, and Aiden EL (2016). Juicebox provides a visualization system for hi-C contact maps with unlimited zoom. Cell Syst 3, 99–101. 27467250 10.1016/j.cels.2015.07.012 PMC5596920

[66] Tareen A, and Kinney JB (2020). Logomaker: beautiful sequence logos in Python. Bioinformatics 36, 2272–2274. 31821414 10.1093/bioinformatics/btz921 PMC7141850

