# Supplementary material for: Single-cell multiome of the human retina and deep learning nominate causal variants in complex eye diseases
Source: Cell Genom. 2022 Jul 27;2(8):100164. doi: 10.1016/j.xgen.2022.100164 (PMC9584034; doi:10.1016/j.xgen.2022.100164)
Supplement: Document S1. Figures S1–S10 and Tables S1–S3 [file mmc1.pdf]

Cell Genomics, Volume *2*

Supplemental information

Single-cell multiome of the human retina and deep learning nominate causal variants in complex eye diseases

Sean K. Wang, Surag Nair, Rui Li, Katerina Kraft, Anusri Pampari, Aman Patel, Joyce B. Kang, Christy Luong, Anshul Kundaje, and Howard Y. Chang

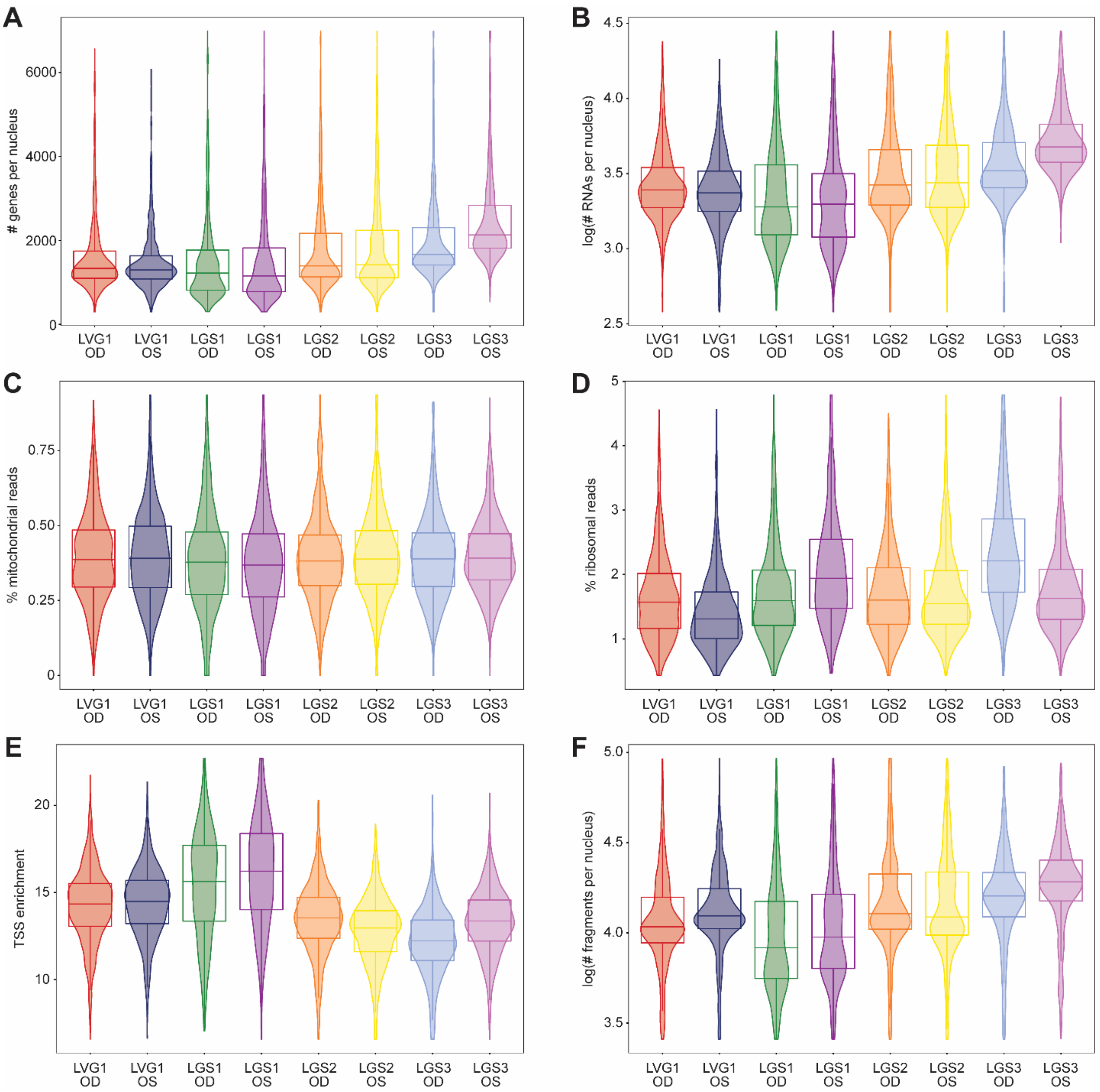

Figure S1. Single-cell RNA- and ATAC-seq quality control metrics, Related to STAR Methods.

(A-F) Violin plots depicting the number of detected genes per nucleus (A), number of detected RNA transcripts per nucleus (B), percentage of mitochondrial reads (C), percentage of ribosomal reads (D), TSS enrichment (E), and number of detected fragments per nucleus (F) by retina in the final dataset. Boxes depict the 25th percentile, median, and 75th percentile of each metric.

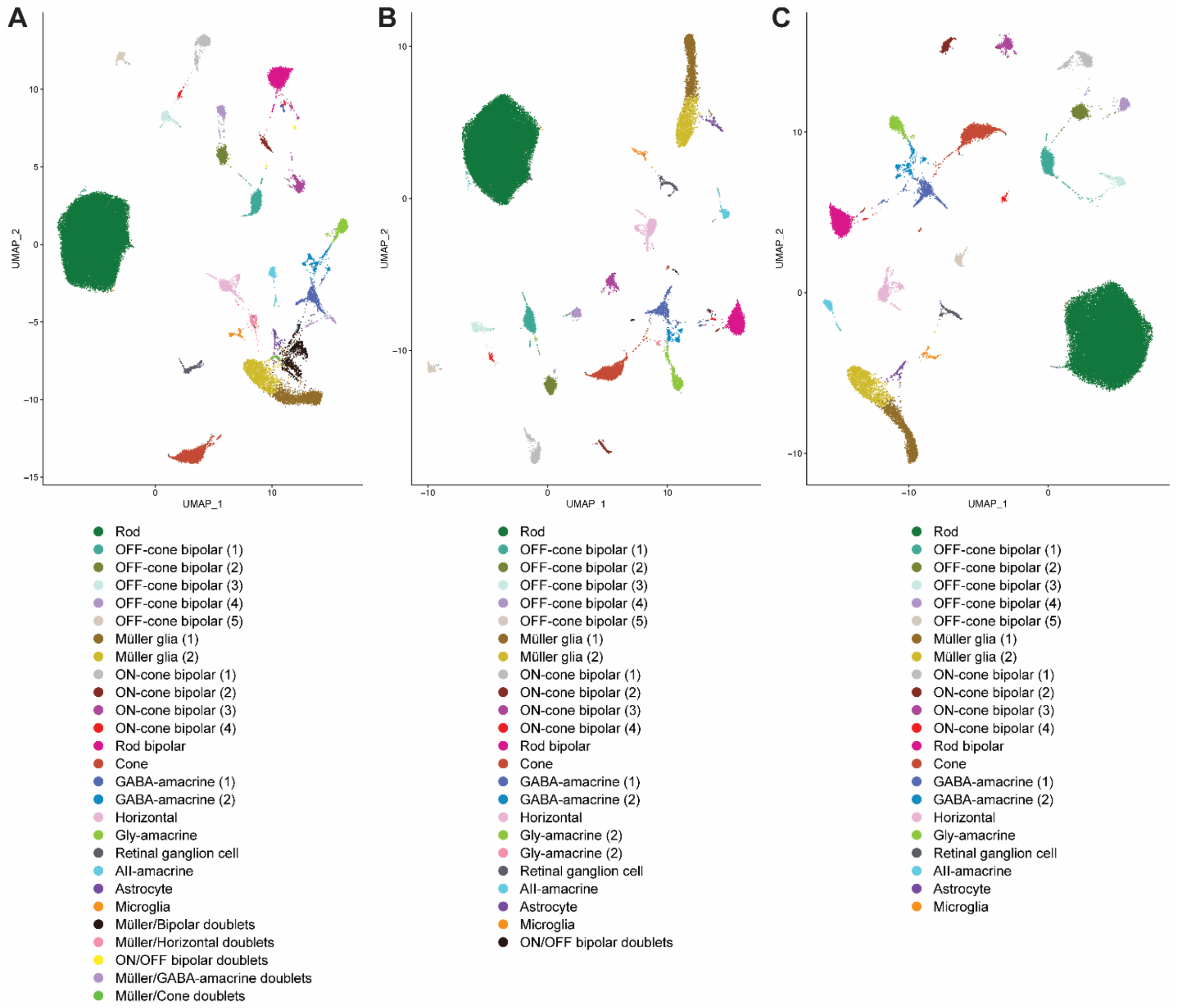

Figure S2. Single-cell RNA-seq cluster assignments, Related to Figure 1.

(A) First iteration uniform manifold approximation and projection (UMAP) plot of the scRNA-seq dataset after quality control filtering, automated removal of doublets, and exclusion of clusters with no detected marker genes. Five clusters comprised of putative doublets were subsequently removed and the dataset re-clustered. **(B)** Second iteration UMAP of the scRNA-seq dataset. One cluster comprised of putative doublets was subsequently removed and the dataset re-clustered. **(C)** Final iteration UMAP of the scRNA-seq dataset. Clusters representing subpopulations of the same cell type were grouped together for downstream analyses.

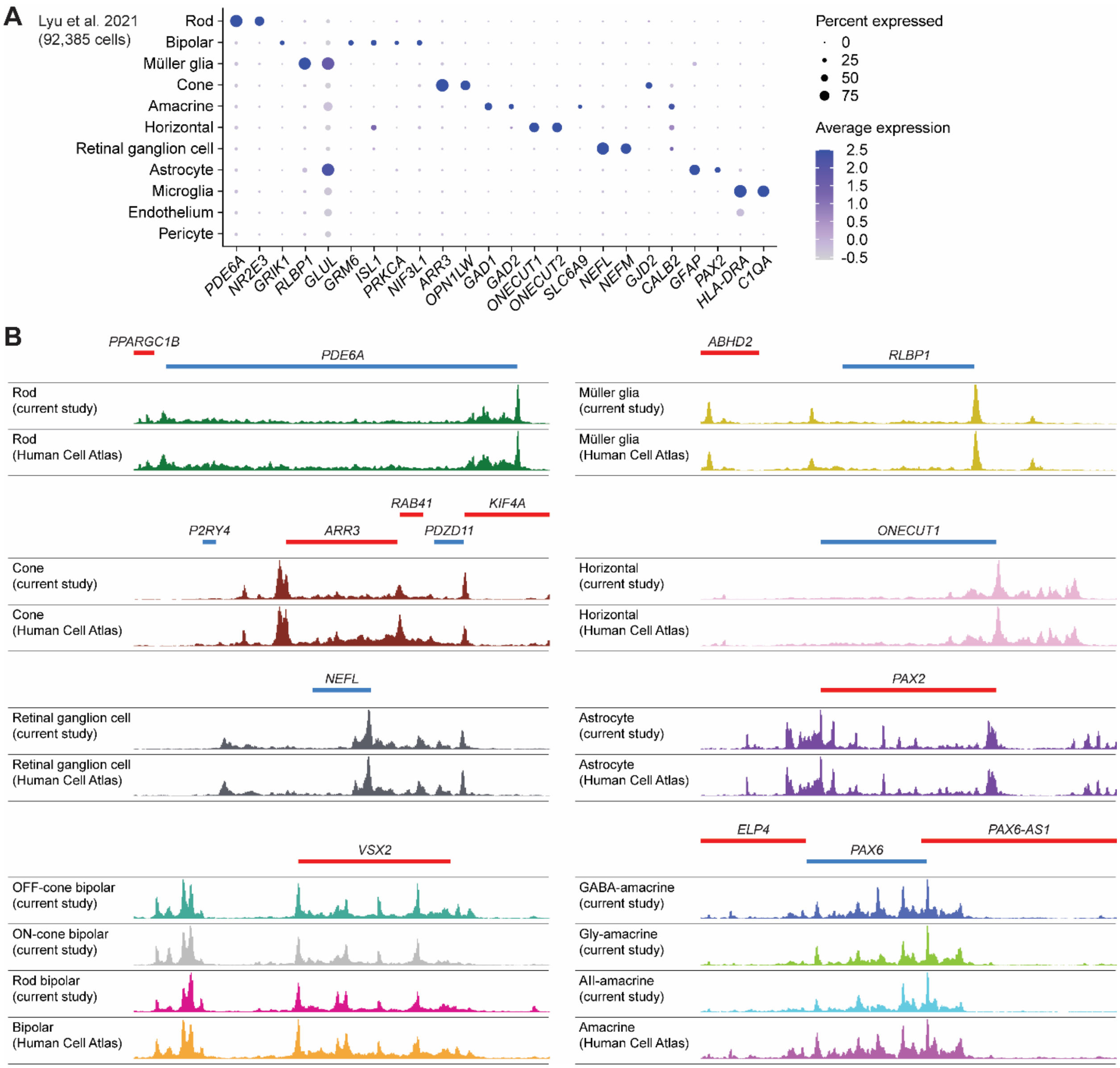

Figure S3. Comparison of single-cell multiome with public datasets, Related to Figures 1 and 2.

(**A**) Dot plot visualizing the normalized RNA expression of the same marker genes from Figure 1D in an adult human retina scRNA-seq dataset from Lyu et al.[1] (**B**) Sequencing tracks of cell type-specific chromatin accessibility near selected marker genes from the current study or an adult human retina scATAC-seq dataset from the Human Cell Atlas (https://retina-atac.cells.ucsc.edu). Tracks from the Human Cell Atlas were aligned to the hg19 genome and matched to hg38 coordinates for visualization.

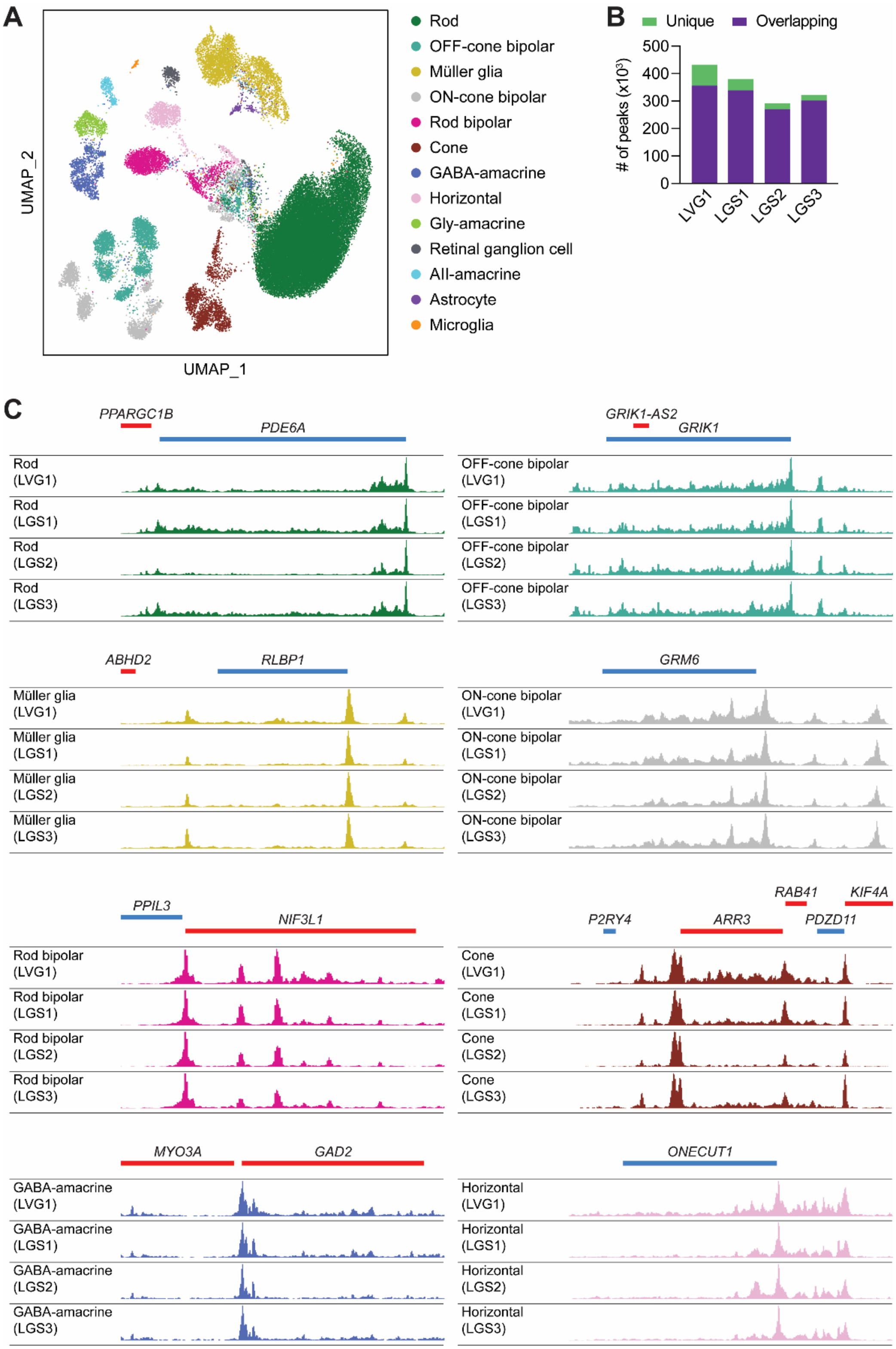

Figure S4. Validation of scATAC-seq data across donors, Related to Figure 2.

(**A**) UMAP plot of the 51,645 human retinal cells based on scATAC-seq profiles. Cell identities were determined by scRNA-seq. (**B**) Overlap of scATAC peaks identified by independent

analysis of each donor. Overlapping was defined as having any overlapping bases with a peak from another donor. (**C**) Sequencing tracks of cell type-specific chromatin accessibility near selected marker genes by donor. Tracks were generated for cell types with >200 cells per donor using non-batch-corrected scATAC data.

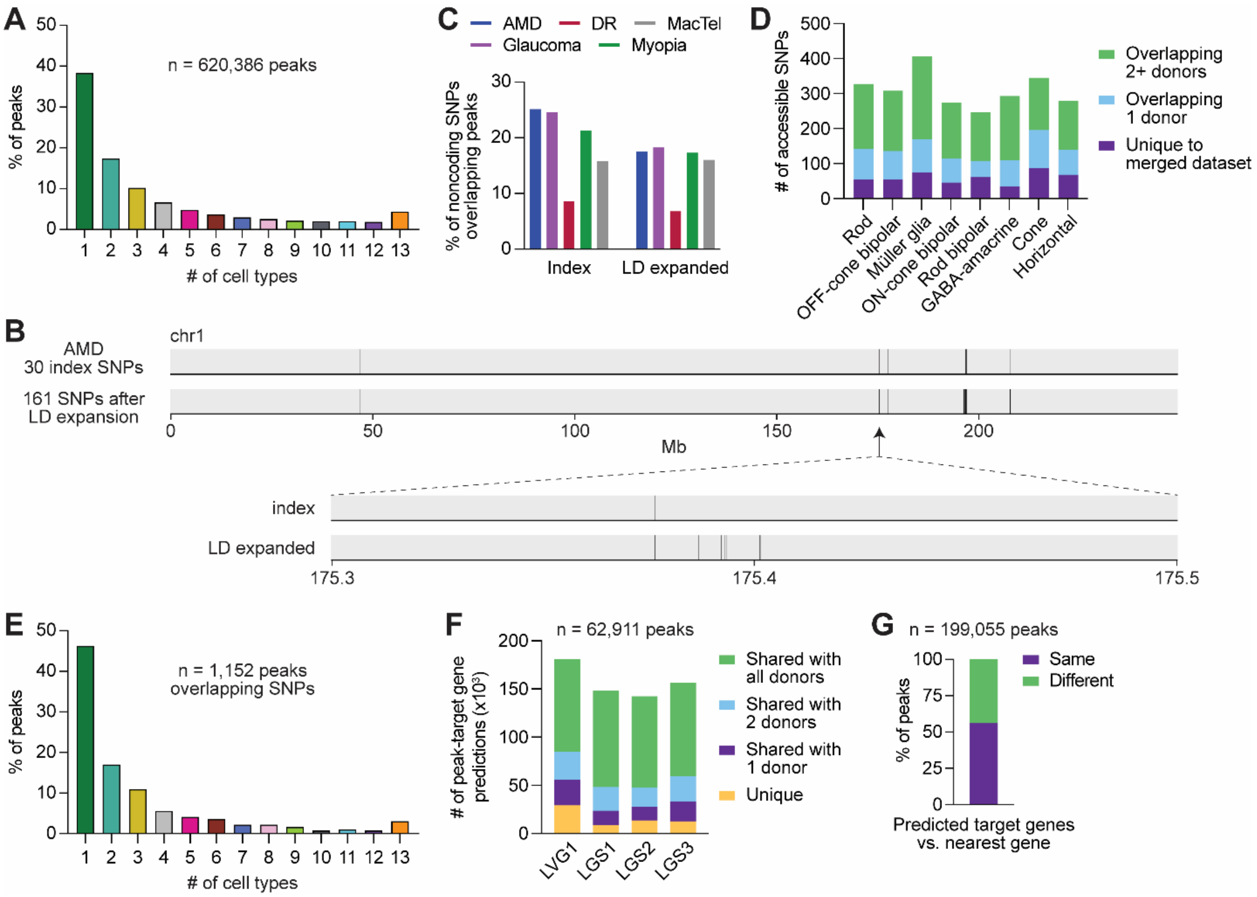

Figure S5. Characterization of chromatin accessibility peaks and linkage disequilibrium expanded SNPs, Related to Figures 2 and 4.

(A) Number of cell types exhibiting each of the 620,386 scATAC peaks. **(B)** Visual depiction of LD expansion for the 30 index SNPs on chromosome 1 associated with AMD. Each vertical black line represents a SNP. **(C)** Percentage of noncoding index and LD expanded SNPs from each disease that overlapped with at least one scATAC peak. **(D)** Number of accessible SNPs from the merged dataset that overlapped with donor-specific scATAC peaks in the indicated cell types. Analysis was restricted to cell types with >200 cells in each of the four donors. (**E**) Number of cell types exhibiting each of the 1,152 scATAC peaks overlapping with a LD expanded SNP. (**F**) Comparison of peak-target gene predictions by donor for the 62,911 scATAC peaks that were accessible in all donors and had at least one predicted target gene. (**G**) Comparison of predicted target genes with the nearest gene for each chromatin accessibility peak with at least one predicted target gene. Same was defined as any of the predicted target genes being the nearest gene.

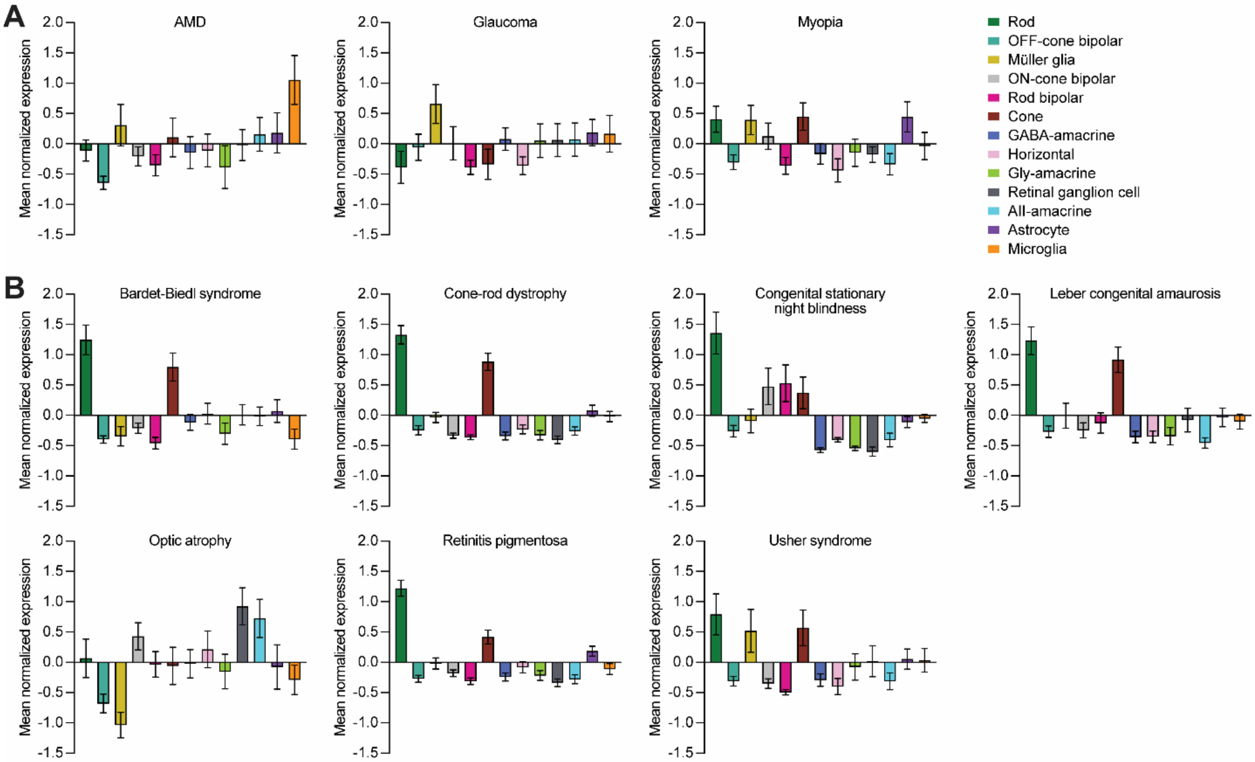

Figure S6. Cell type expression of coding variants in complex and monogenic eye diseases, Related to Figure 4.

(**A**, **B**) Mean normalized RNA expression by cell type of genes containing coding variants implicated in complex eye diseases (A) or monogenic retinal diseases (B). Coding variants were defined as variants resulting in an amino acid change or premature stop codon. Gene expression analysis was restricted to diseases with ten or more implicated genes. Monogenic retinal disease genes were obtained from the Retinal Information Network (https://sph.uth.edu/retnet).[2] Genes containing coding variants for each disease are listed in Data S5. Error bars denote standard error of the mean.

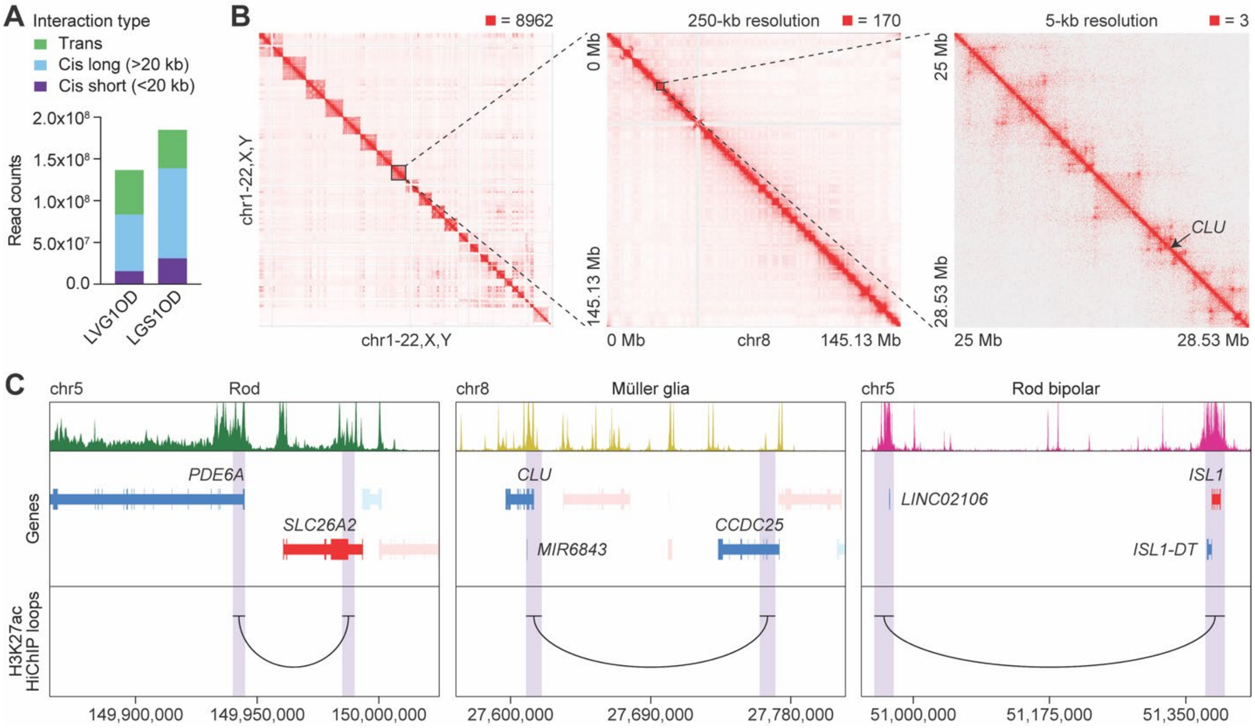

Figure S7. H3K27ac HiChIP predicts enhancer-promoter interactions in the human retina, Related to Figure 5.

(A) HiChIP interaction types by sample. Cis refers to interactions on the same chromosome, while trans refers to interactions spanning separate chromosomes. **(B)** HiChIP interaction maps at whole genome, 250-kb, and 5-kb resolution. Sample shown is LGS1OD. Signal was normalized to the square root of coverage. Numbers above the interaction maps indicate maximum signal in each matrix. **(C)** Sequencing tracks of cell type chromatin accessibility and H3K27ac HiChIP loops overlapping with the TSS of selected marker genes. Genes in the sense and antisense directions are shown in red and blue, respectively. Regions encompassed by the loop anchors are highlighted in purple.

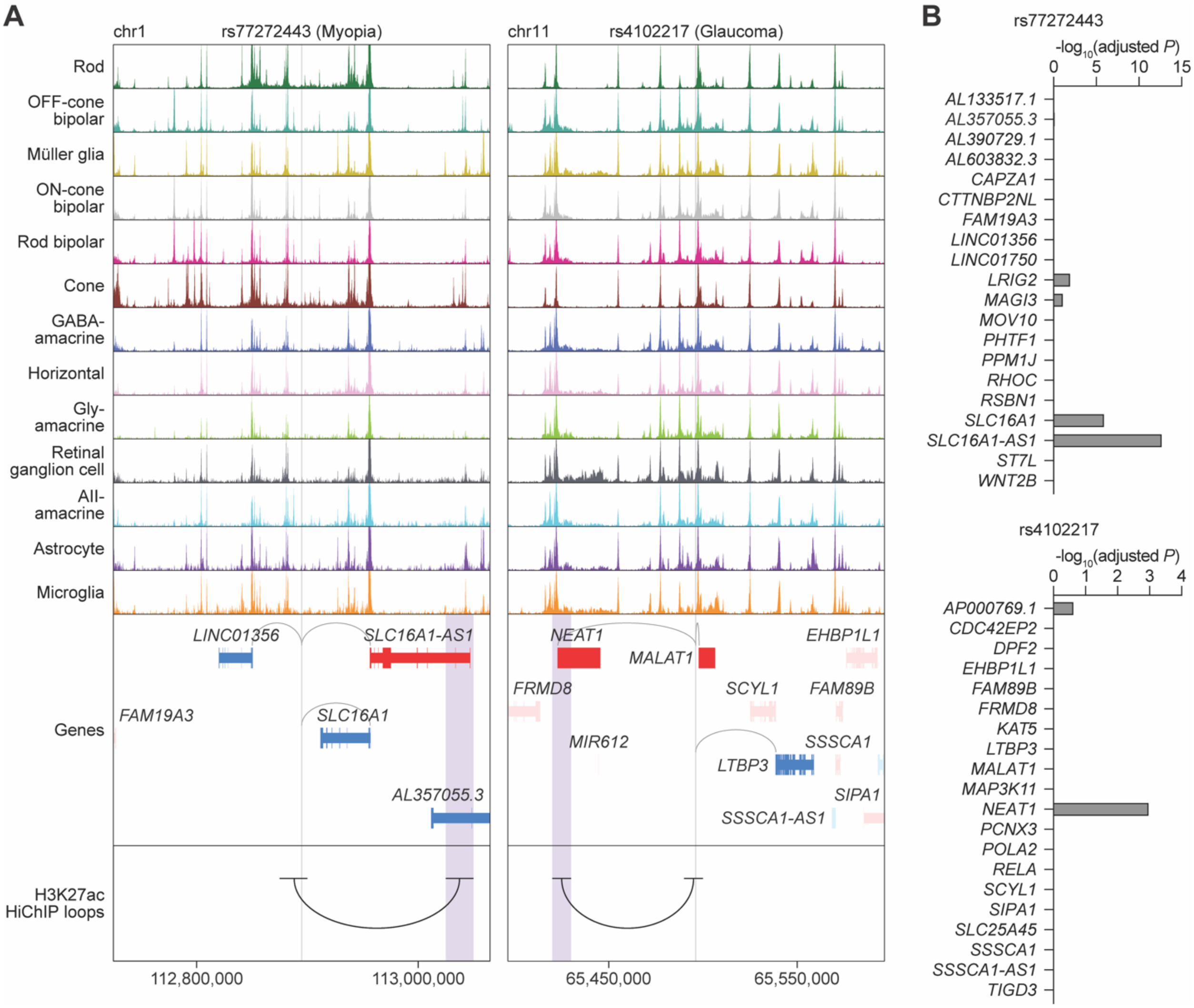

Figure S8. Integration of single-cell multiome with HiChIP and eQTL data refines SNP-target gene predictions, Related to Figure 5.

(A) Sequencing tracks of chromatin accessibility near rs77272443 (chr1:112894884) and rs4102217 (chr11:65496424). Genes in the sense and antisense directions are shown in red and blue, respectively. The location of each SNP is depicted by a vertical gray line. Gray arcs indicate predicted target genes for the scATAC peak containing the SNP of interest. Black arcs overlapping with SNPs indicate H3K27ac HiChIP loops with the regions encompassed by the opposite anchors highlighted in purple. **(B)** Significance of SNP-gene associations for rs77272443 or rs4102217 and their 20 nearest genes as determined by retina eQTL analysis. Adjusted P values for each gene were calculated by multiplying the nominal P value listed in the EyeGEx database by the number of SNP-gene pairs tested for that SNP.

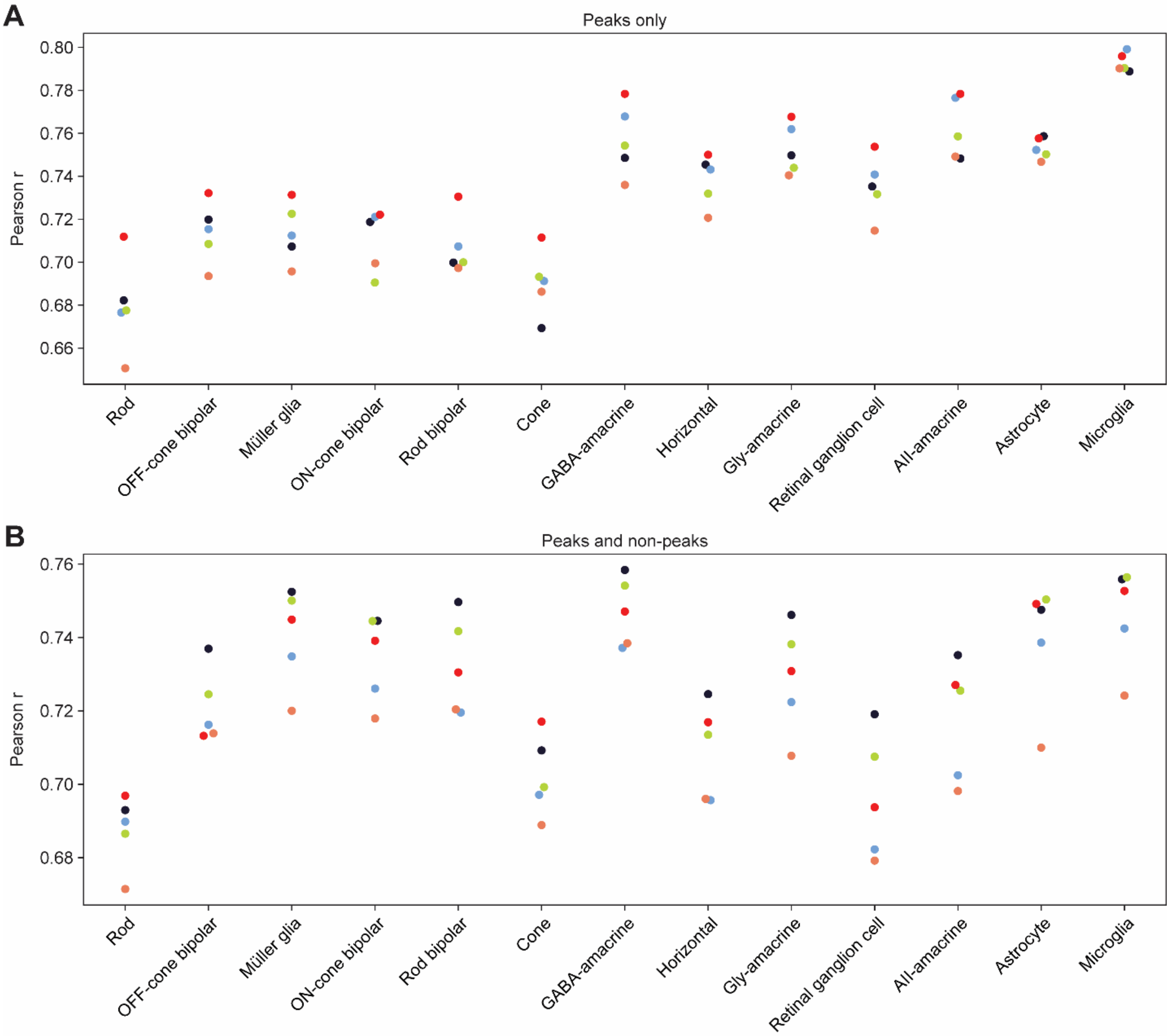

Figure S9. BPNet model performance, Related to Figure 6.

(A, B) Correlation scores between predicted and actual observed log counts in only peak (A) or both peak and non-peak (B) regions on chromosome sequences that were withheld during cell type-specific model training. Each color represents one of five model folds.

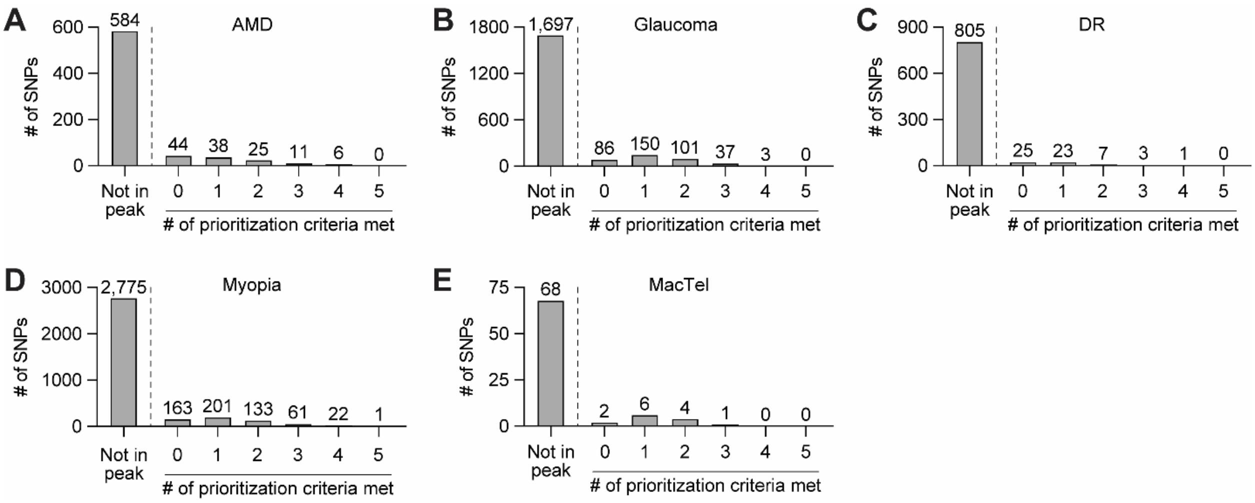

Figure S10. Summary of SNP prioritization, Related to Figures 4-6.

(A-E) Total number of prioritization criteria met by LD expanded SNPs in loci associated with AMD (A), glaucoma (B), DR (C), myopia (D), and MacTel (E). Prioritization criteria were 1) co-accessibility with a promoter peak, 2) accessibility correlated with expression of a nearby gene, 3) linkage to a gene by H3K27ac HiChIP, 4) significant association with a gene by retina eQTL data, and 5) high effect designation by deep learning.

Table S1. Donor information, Related to STAR Methods

ID	Age	Sex	Death-to-preservation interval	Cause of death
LVG1	55	M	9 hours	Cardiac arrest
LGS1	85	F	11 hours	Sepsis
LGS2	87	M	9 hours	Fall
LGS3	74	F	12 hours	Dementia

Table S2. Cell counts per retina, Related to Figure 1

Cell type	LVG1 OD	LVG1 OS	LGS1 OD	LGS1 OS	LGS2 OD	LGS2 OS	LGS3 OD	LGS3 OD
Rod	3151	3061	6132	6777	3409	3190	3299	3040
OFF-cone bipolar	398	373	447	558	557	622	549	432
Müller glia	384	372	496	448	608	559	436	367
ON-cone bipolar	223	245	299	327	449	393	373	308
Rod bipolar	234	190	358	263	350	302	313	314
Cone	197	164	335	386	325	365	240	211
GABA-amacrine	194	135	257	257	169	240	171	120
Horizontal	116	107	114	129	194	124	174	177
Gly-amacrine	83	66	114	138	77	106	80	66
Retinal ganglion cell	98	54	26	20	116	53	100	26
AII-amacrine	42	53	94	72	41	83	43	23
Astrocyte	40	35	4	8	31	19	59	36
Microglia	15	13	33	22	39	51	29	30
Total	5175	4868	8709	9405	6365	6107	5866	5150

Table S3. First author and year of GWAS by disease, Related to Figure 4

AMD	Glaucoma	DR	Myopia	MacTel
Klein 2005[3]	Thorleifsson 2007[4]	Huang 2011[5]	Nakanishi 2009[6]	Scerri 2017[7]
Chen 2010[8]	Meguro 2010[9]	Grassi 2011[10]	Li 2011[11]	Bonelli 2021[12]
Neale 2010[13]	Burdon 2011[14]	Sheu 2013[15]	Li 2011[16]	
Kopplin 2010[17]	Nakano 2012[18]	Awata 2014[19]	Shi 2011[20]	
Yu 2011[21]	Osman 2012[22]	Burdon 2015[23]	Fan 2012[24]	
Arakawa 2011[25]	Wiggs 2012[26]	Graham 2018[27]	Meng 2012[28]	
Cipriani 2012[29]	Takamoto 2012[30]	Meng 2018[31]	Shi 2013[32]	
Sobrin 2012[33]	Vithana 2012[34]	Pollack 2019[35]	Khor 2013[36]	
Holliday 2013[37]	Nakano 2014[38]	Meng 2019[39]	Simpson 2014[40]	
Fritsche 2013[41]	Hoffmann 2014[42]	Liu 2019[43]	Pickrell 2016[44]	
Naj 2013[45]	Chen 2014[46]	Hsieh 2020[47]	Tedja 2018[48]	
Cheng 2015[49]	Gharahkhani 2014[50]	Imamura 2021[51]	Boutin 2020[52]	
Fritsche 2016[53]	Aung 2015[54]		Meguro 2020[55]	
Ruamviboonsuk 2017[56]	Li 2015[57]		Tideman 2021[58]	
Persad 2017[59]	Vishal 2016[60]			
Yan 2018[61]	Khor 2016[62]			
Han 2020[63]	Verma 2016[64]			
Winkler 2020[65]	Bailey 2016[66]			
Guenther 2020[67]	Zagajewska 2018[68]			
	Gharahkhani 2018[69]			
	Choquet 2018[70]			
	MacGregor 2018[71]			
	Bonnemaijer 2018[72]			
	Zhou 2018[73]			
	Shiga 2018[74]			
	Hauser 2019[75]			
	Craig 2020[76]			
	Kim 2020[77]			
	Ishigaki 2020[78]			
	Gharahkhani 2021[79]			
	Sakaue 2021[80]			

SUPPLEMENTAL REFERENCES

1. Lyu, Y., Zauhar, R., Dana, N., Strang, C.E., Hu, J., Wang, K., Liu, S., Pan, N., Gamlin, P., Kimble, J.A., *et al.* (2021). Implication of specific retinal cell-type involvement and gene expression changes in AMD progression using integrative analysis of single-cell and bulk RNA-seq profiling. Sci. Rep. *11*, 15612.

2. Daiger, S.P., Sullivan, L.S., Bowne, S.J. (2013). Genes and mutations causing retinitis pigmentosa. Clin. Genet. *84*, 132–141.

3. Klein, R.J., Zeiss, C., Chew, E.Y., Tsai, J.-Y., Sackler, R.S., Haynes, C., Henning, A.K., SanGiovanni, J.P., Mane, S.M., Mayne, S.T., *et al.* (2005). Complement factor H polymorphism in age-related macular degeneration. Science *308*, 385–9.

4. Thorleifsson, G., Magnusson, K.P., Sulem, P., Walters, G.B., Gudbjartsson, D.F., Stefansson, H., Jonsson, T., Jonasdottir, A., Jonasdottir, A., Stefansdottir, G., *et al.* (2007). Common sequence variants in the LOXL1 gene confer susceptibility to exfoliation glaucoma. Science *317*, 1397–400.

5. Huang, Y.-C., Lin, J.-M., Lin, H.-J., Chen, C.-C., Chen, S.-Y., Tsai, C.-H., Tsai, F.-J. (2011). Genome-wide association study of diabetic retinopathy in a Taiwanese population. Ophthalmology *118*, 642–8.

6. Nakanishi, H., Yamada, R., Gotoh, N., Hayashi, H., Yamashiro, K., Shimada, N., Ohno-Matsui, K., Mochizuki, M., Saito, M., Iida, T., *et al.* (2009). A genome-wide association analysis identified a novel susceptible locus for pathological myopia at 11q24.1. PLoS Genet. *5*, e1000660.

7. Scerri, T.S., Quaglieri, A., Cai, C., Zernant, J., Matsunami, N., Baird, L., Scheppke, L., Bonelli, R., Yannuzzi, L.A., Friedlander, M., *et al.* (2017). Genome-wide analyses identify common variants associated with macular telangiectasia type 2. Nat. Genet. *49*, 559–567.

8. Chen, W., Stambolian, D., Edwards, A.O., Branham, K.E., Othman, M., Jakobsdottir, J., Tosakulwong, N., Pericak-Vance, M.A., Campochiaro, P.A., Klein, M.L., *et al.* (2010). Genetic variants near TIMP3 and high-density lipoprotein-associated loci influence susceptibility to age-related macular degeneration. Proc. Natl. Acad. Sci. U. S. A. *107*, 7401–6.

9. Meguro, A., Inoko, H., Ota, M., Mizuki, N., Bahram, S. (2010). Genome-wide association study of normal tension glaucoma: common variants in SRBD1 and ELOVL5 contribute to disease susceptibility. Ophthalmology *117*, 1331–8.e5.

10. Grassi, M.A., Tikhomirov, A., Ramalingam, S., Below, J.E., Cox, N.J., Nicolae, D.L. (2011). Genome-wide meta-analysis for severe diabetic retinopathy. Hum. Mol. Genet. *20*, 2472–81.

11. Li, Y.-J., Goh, L., Khor, C.-C., Fan, Q., Yu, M., Han, S., Sim, X., Ong, R.T.-H., Wong, T.-Y., Vithana, E.N., *et al.* (2011). Genome-wide association studies reveal genetic variants in CTNND2 for high myopia in Singapore Chinese. Ophthalmology *118*, 368–75.

12. Bonelli, R., Jackson, V.E., Prasad, A., Munro, J.E., Farashi, S., Heeren, T.F.C., Pontikos, N., Scheppke, L., Friedlander, M., MacTel Consortium, *et al.* (2021). Identification of genetic factors influencing metabolic dysregulation and retinal support for MacTel, a retinal disorder. Commun. Biol. *4*, 274.

13. Neale, B.M., Fagerness, J., Reynolds, R., Sobrin, L., Parker, M., Raychaudhuri, S., Tan, P.L., Oh, E.C., Merriam, J.E., Souied, E., *et al.* (2010). Genome-wide association study of advanced age-related macular degeneration identifies a role of the hepatic lipase gene (LIPC). Proc. Natl. Acad. Sci. U. S. A. *107*, 7395–400.

14. Burdon, K.P., Macgregor, S., Hewitt, A.W., Sharma, S., Chidlow, G., Mills, R.A., Danoy, P., Casson, R., Viswanathan, A.C., Liu, J.Z., *et al.* (2011). Genome-wide association study identifies susceptibility loci for open angle glaucoma at TMCO1 and CDKN2B-AS1. Nat. Genet. *43*, 574–8.

15. Sheu, W.H.-H., Kuo, J.Z., Lee, I.-T., Hung, Y.-J., Lee, W.-J., Tsai, H.-Y., Wang, J.-S., Goodarzi, M.O., Klein, R., Klein, B.E.K., *et al.* (2013). Genome-wide association study in a Chinese population with diabetic retinopathy. Hum. Mol. Genet. *22*, 3165–73.

16. Li, Z., Qu, J., Xu, X., Zhou, X., Zou, H., Wang, N., Li, T., Hu, X., Zhao, Q., Chen, P., *et al.* (2011). A genome-wide association study reveals association between common variants in an intergenic region of 4q25 and high-grade myopia in the Chinese Han population. Hum. Mol. Genet. *20*, 2861–8.

17. Kopplin, L.J., Igo, R.P., Wang, Y., Sivakumaran, T.A., Hagstrom, S.A., Peachey, N.S., Francis, P.J., Klein, M.L., SanGiovanni, J.P., Chew, E.Y., *et al.* (2010). Genome-wide association identifies SKIV2L and MYRIP as protective factors for age-related macular degeneration. Genes Immun. *11*, 609–21.

18. Nakano, M., Ikeda, Y., Tokuda, Y., Fuwa, M., Omi, N., Ueno, M., Imai, K., Adachi, H., Kageyama, M., Mori, K., *et al.* (2012). Common variants in CDKN2B-AS1 associated with optic-nerve vulnerability of glaucoma identified by genome-wide association studies in Japanese. PLoS One *7*, e33389.

19. Awata, T., Yamashita, H., Kurihara, S., Morita-Ohkubo, T., Miyashita, Y., Katayama, S., Mori, K., Yoneya, S., Kohda, M., Okazaki, Y., *et al.* (2014). A genome-wide association study for diabetic retinopathy in a Japanese population: potential association with a long intergenic non-coding RNA. PLoS One *9*, e111715.

20. Shi, Y., Qu, J., Zhang, D., Zhao, P., Zhang, Q., Tam, P.O.S., Sun, L., Zuo, X., Zhou, X., Xiao, X., *et al.* (2011). Genetic variants at 13q12.12 are associated with high myopia in the Han Chinese population. Am. J. Hum. Genet. *88*, 805–813.

21. Yu, Y., Bhangale, T.R., Fagerness, J., Ripke, S., Thorleifsson, G., Tan, P.L., Souied, E.H., Richardson, A.J., Merriam, J.E., Buitendijk, G.H.S., *et al.* (2011). Common variants near FRK/COL10A1 and VEGFA are associated with advanced age-related macular degeneration. Hum. Mol. Genet. *20*, 3699–709.

22. Osman, W., Low, S.-K., Takahashi, A., Kubo, M., Nakamura, Y. (2012). A genome-wide association study in the Japanese population confirms 9p21 and 14q23 as susceptibility loci for primary open angle glaucoma. Hum. Mol. Genet. *21*, 2836–42.

23. Burdon, K.P., Fogarty, R.D., Shen, W., Abhary, S., Kaidonis, G., Appukuttan, B., Hewitt, A.W., Sharma, S., Daniell, M., Essex, R.W., *et al.* (2015). Genome-wide association study for sight-threatening diabetic retinopathy reveals association with genetic variation near the GRB2 gene. Diabetologia *58*, 2288–97.

24. Fan, Q., Barathi, V.A., Cheng, C.-Y., Zhou, X., Meguro, A., Nakata, I., Khor, C.-C., Goh, L.-

K., Li, Y.-J., Lim, W., *et al.* (2012). Genetic variants on chromosome 1q41 influence ocular axial length and high myopia. PLoS Genet. *8*, e1002753.

25. Arakawa, S., Takahashi, A., Ashikawa, K., Hosono, N., Aoi, T., Yasuda, M., Oshima, Y., Yoshida, S., Enaida, H., Tsuchihashi, T., *et al.* (2011). Genome-wide association study identifies two susceptibility loci for exudative age-related macular degeneration in the Japanese population. Nat. Genet. *43*, 1001–4.

26. Wiggs, J.L., Yaspan, B.L., Hauser, M.A., Kang, J.H., Allingham, R.R., Olson, L.M., Abdrabou, W., Fan, B.J., Wang, D.Y., Brodeur, W., *et al.* (2012). Common variants at 9p21 and 8q22 are associated with increased susceptibility to optic nerve degeneration in glaucoma. PLoS Genet. *8*, e1002654.

27. Graham, P.S., Kaidonis, G., Abhary, S., Gillies, M.C., Daniell, M., Essex, R.W., Chang, J.H., Lake, S.R., Pal, B., Jenkins, A.J., *et al.* (2018). Genome-wide association studies for diabetic macular edema and proliferative diabetic retinopathy. BMC Med. Genet. *19*, 71.

28. Meng, W., Butterworth, J., Bradley, D.T., Hughes, A.E., Soler, V., Calvas, P., Malecaze, F. (2012). A genome-wide association study provides evidence for association of chromosome 8p23 (MYP10) and 10q21.1 (MYP15) with high myopia in the French Population. Invest. Ophthalmol. Vis. Sci. *53*, 7983–8.

29. Cipriani, V., Leung, H.-T., Plagnol, V., Bunce, C., Khan, J.C., Shahid, H., Moore, A.T., Harding, S.P., Bishop, P.N., Hayward, C., *et al.* (2012). Genome-wide association study of age-related macular degeneration identifies associated variants in the TNXB-FKBPL-NOTCH4 region of chromosome 6p21.3. Hum. Mol. Genet. *21*, 4138–50.

30. Takamoto, M., Kaburaki, T., Mabuchi, A., Araie, M., Amano, S., Aihara, M., Tomidokoro, A., Iwase, A., Mabuchi, F., Kashiwagi, K., *et al.* (2012). Common variants on chromosome 9p21 are associated with normal tension glaucoma. PLoS One *7*, e40107.

31. Meng, W., Shah, K.P., Pollack, S., Toppila, I., Hebert, H.L., McCarthy, M.I., Groop, L., Ahlqvist, E., Lyssenko, V., Agardh, E., *et al.* (2018). A genome-wide association study suggests new evidence for an association of the NADPH Oxidase 4 (NOX4) gene with severe diabetic retinopathy in type 2 diabetes. Acta Ophthalmol. *96*, e811–e819.

32. Shi, Y., Gong, B., Chen, L., Zuo, X., Liu, X., Tam, P.O.S., Zhou, X., Zhao, P., Lu, F., Qu, J., *et al.* (2013). A genome-wide meta-analysis identifies two novel loci associated with high myopia in the Han Chinese population. Hum. Mol. Genet. *22*, 2325–33.

33. Sobrin, L., Ripke, S., Yu, Y., Fagerness, J., Bhangale, T.R., Tan, P.L., Souied, E.H., Buitendijk, G.H.S., Merriam, J.E., Richardson, A.J., *et al.* (2012). Heritability and genome-wide association study to assess genetic differences between advanced age-related macular degeneration subtypes. Ophthalmology *119*, 1874–85.

34. Vithana, E.N., Khor, C.-C., Qiao, C., Nongpiur, M.E., George, R., Chen, L.-J., Do, T., Abu-Amero, K., Huang, C.K., Low, S., *et al.* (2012). Genome-wide association analyses identify three new susceptibility loci for primary angle closure glaucoma. Nat. Genet. *44*, 1142–1146.

35. Pollack, S., Igo, R.P., Jensen, R.A., Christiansen, M., Li, X., Cheng, C.-Y., Ng, M.C.Y., Smith, A. V, Rossin, E.J., Segrè, A. V, *et al.* (2019). Multiethnic Genome-Wide Association Study of Diabetic Retinopathy Using Liability Threshold Modeling of Duration of Diabetes and Glycemic Control. Diabetes *68*, 441–456.

36. Khor, C.C., Miyake, M., Chen, L.J., Shi, Y., Barathi, V.A., Qiao, F., Nakata, I., Yamashiro, K., Zhou, X., Tam, P.O.S., *et al.* (2013). Genome-wide association study identifies ZFHX1B as a susceptibility locus for severe myopia. Hum. Mol. Genet. *22*, 5288–94.

37. Holliday, E.G., Smith, A. V, Cornes, B.K., Buitendijk, G.H.S., Jensen, R.A., Sim, X., Aspelund, T., Aung, T., Baird, P.N., Boerwinkle, E., *et al.* (2013). Insights into the genetic architecture of early stage age-related macular degeneration: a genome-wide association study meta-analysis. PLoS One *8*, e53830.

38. Nakano, M., Ikeda, Y., Tokuda, Y., Fuwa, M., Ueno, M., Imai, K., Sato, R., Omi, N., Adachi, H., Kageyama, M., *et al.* (2014). Novel common variants and susceptible haplotype for exfoliation glaucoma specific to Asian population. Sci. Rep. *4*, 5340.

39. Meng, W., Chan, B.W., Ezeonwumelu, C., Hébert, H.L., Campbell, A., Soler, V., Palmer, C.N. (2019). A genome-wide association study implicates that the TTC39C gene is associated with diabetic maculopathy with decreased visual acuity. Ophthalmic Genet. *40*, 252–258.

40. Simpson, C.L., Wojciechowski, R., Oexle, K., Murgia, F., Portas, L., Li, X., Verhoeven, V.J.M., Vitart, V., Schache, M., Hosseini, S.M., *et al.* (2014). Genome-wide meta-analysis of myopia and hyperopia provides evidence for replication of 11 loci. PLoS One *9*, e107110.

41. Fritsche, L.G., Chen, W., Schu, M., Yaspan, B.L., Yu, Y., Thorleifsson, G., Zack, D.J., Arakawa, S., Cipriani, V., Ripke, S., *et al.* (2013). Seven new loci associated with age-related macular degeneration. Nat. Genet. *45*, 433–9, 439e1-2.

42. Hoffmann, T.J., Tang, H., Thornton, T.A., Caan, B., Haan, M., Millen, A.E., Thomas, F., Risch, N. (2014). Genome-wide association and admixture analysis of glaucoma in the Women's Health Initiative. Hum. Mol. Genet. *23*, 6634–43.

43. Liu, C., Chen, G., Bentley, A.R., Doumatey, A., Zhou, J., Adeyemo, A., Yang, J., Rotimi, C. (2019). Genome-wide association study for proliferative diabetic retinopathy in Africans. NPJ genomic Med. *4*, 20.

44. Pickrell, J.K., Berisa, T., Liu, J.Z., Ségurel, L., Tung, J.Y., Hinds, D.A. (2016). Detection and interpretation of shared genetic influences on 42 human traits. Nat. Genet. *48*, 709–17.

45. Naj, A.C., Scott, W.K., Courtenay, M.D., Cade, W.H., Schwartz, S.G., Kovach, J.L., Agarwal, A., Wang, G., Haines, J.L., Pericak-Vance, M.A. (2013). Genetic factors in nonsmokers with age-related macular degeneration revealed through genome-wide gene-environment interaction analysis. Ann. Hum. Genet. *77*, 215–31.

46. Chen, Y., Lin, Y., Vithana, E.N., Jia, L., Zuo, X., Wong, T.Y., Chen, L.J., Zhu, X., Tam, P.O.S., Gong, B., *et al.* (2014). Common variants near ABCA1 and in PMM2 are associated with primary open-angle glaucoma. Nat. Genet. *46*, 1115–9.

47. Hsieh, A.-R., Huang, Y.-C., Yang, Y.-F., Lin, H.-J., Lin, J.-M., Chang, Y.-W., Wu, C.-M., Liao, W.-L., Tsai, F.-J. (2020). Lack of association of genetic variants for diabetic retinopathy in Taiwanese patients with diabetic nephropathy. BMJ open diabetes Res. care *8*.

48. Tedja, M.S., Wojciechowski, R., Hysi, P.G., Eriksson, N., Furlotte, N.A., Verhoeven, V.J.M., Iglesias, A.I., Meester-Smoor, M.A., Tompson, S.W., Fan, Q., *et al.* (2018). Genome-wide association meta-analysis highlights light-induced signaling as a driver for refractive error. Nat. Genet. *50*, 834–848.

49. Cheng, C.-Y., Yamashiro, K., Chen, L.J., Ahn, J., Huang, L., Huang, L., Cheung, C.M.G., Miyake, M., Cackett, P.D., Yeo, I.Y., *et al.* (2015). New loci and coding variants confer risk for age-related macular degeneration in East Asians. Nat. Commun. *6*, 6063.

50. Gharahkhani, P., Burdon, K.P., Fogarty, R., Sharma, S., Hewitt, A.W., Martin, S., Law, M.H., Cremin, K., Bailey, J.N.C., Loomis, S.J., *et al.* (2014). Common variants near ABCA1, AFAP1 and GMDS confer risk of primary open-angle glaucoma. Nat. Genet. *46*, 1120–1125.

51. Imamura, M., Takahashi, A., Matsunami, M., Horikoshi, M., Iwata, M., Araki, S.-I., Toyoda, M., Susarla, G., Ahn, J., Park, K.H., *et al.* (2021). Genome-wide association studies identify two novel loci conferring susceptibility to diabetic retinopathy in Japanese patients with type 2 diabetes. Hum. Mol. Genet. *30*, 716–726.

52. Boutin, T.S., Charteris, D.G., Chandra, A., Campbell, S., Hayward, C., Campbell, A., UK Biobank Eye & Vision Consortium, Nandakumar, P., Hinds, D., 23andMe Research Team, *et al.* (2020). Insights into the genetic basis of retinal detachment. Hum. Mol. Genet. *29*, 689–702.

53. Fritsche, L.G., Igl, W., Bailey, J.N.C., Grassmann, F., Sengupta, S., Bragg-Gresham, J.L., Burdon, K.P., Hebbring, S.J., Wen, C., Gorski, M., *et al.* (2016). A large genome-wide association study of age-related macular degeneration highlights contributions of rare and common variants. Nat. Genet. *48*, 134–43.

54. Aung, T., Ozaki, M., Mizoguchi, T., Allingham, R.R., Li, Z., Haripriya, A., Nakano, S., Uebe, S., Harder, J.M., Chan, A.S.Y., *et al.* (2015). A common variant mapping to CACNA1A is associated with susceptibility to exfoliation syndrome. Nat. Genet. *47*, 387–92.

55. Meguro, A., Yamane, T., Takeuchi, M., Miyake, M., Fan, Q., Zhao, W., Wang, I.-J., Mizuki, Y., Yamada, N., Nomura, N., *et al.* (2020). Genome-Wide Association Study in Asians Identifies Novel Loci for High Myopia and Highlights a Nervous System Role in Its Pathogenesis. Ophthalmology *127*, 1612–1624.

56. Ruamviboonsuk, P., Tadarati, M., Singhanetr, P., Wattanapokayakit, S., Kunhapan, P., Wanitchanon, T., Wichukchinda, N., Mushiroda, T., Akiyama, M., Momozawa, Y., *et al.* (2017). Genome-wide association study of neovascular age-related macular degeneration in the Thai population. J. Hum. Genet. *62*, 957–962.

57. Li, Z., Allingham, R.R., Nakano, M., Jia, L., Chen, Y., Ikeda, Y., Mani, B., Chen, L.-J., Kee, C., Garway-Heath, D.F., *et al.* (2015). A common variant near TGFBR3 is associated with primary open angle glaucoma. Hum. Mol. Genet. *24*, 3880–92.

58. Tideman, J.W.L., Pärssinen, O., Haarman, A.E.G., Khawaja, A.P., Wedenoja, J., Williams, K.M., Biino, G., Ding, X., Kähönen, M., Lehtimäki, T., *et al.* (2021). Evaluation of Shared Genetic Susceptibility to High and Low Myopia and Hyperopia. JAMA Ophthalmol. *139*, 601–609.

59. Persad, P.J., Heid, I.M., Weeks, D.E., Baird, P.N., de Jong, E.K., Haines, J.L., Pericak-Vance, M.A., Scott, W.K., International Age-Related Macular Degeneration Genomics Consortium (IAMDGC) (2017). Joint Analysis of Nuclear and Mitochondrial Variants in Age-Related Macular Degeneration Identifies Novel Loci TRPM1 and ABHD2/RLBP1. Invest. Ophthalmol. Vis. Sci. *58*, 4027–4038.

60. Vishal, M., Sharma, A., Kaurani, L., Alfano, G., Mookherjee, S., Narta, K., Agrawal, J., Bhattacharya, I., Roychoudhury, S., Ray, J., *et al.* (2016). Genetic association and stress

mediated down-regulation in trabecular meshwork implicates MPP7 as a novel candidate gene in primary open angle glaucoma. BMC Med. Genomics *9*, 15.

61. Yan, Q., Ding, Y., Liu, Y., Sun, T., Fritsche, L.G., Clemons, T., Ratnapriya, R., Klein, M.L., Cook, R.J., Liu, Y., *et al.* (2018). Genome-wide analysis of disease progression in age-related macular degeneration. Hum. Mol. Genet. *27*, 929–940.

62. Khor, C.C., Do, T., Jia, H., Nakano, M., George, R., Abu-Amero, K., Duvesh, R., Chen, L.J., Li, Z., Nongpiur, M.E., *et al.* (2016). Genome-wide association study identifies five new susceptibility loci for primary angle closure glaucoma. Nat. Genet. *48*, 556–62.

63. Han, X., Gharahkhani, P., Mitchell, P., Liew, G., Hewitt, A.W., MacGregor, S. (2020). Genome-wide meta-analysis identifies novel loci associated with age-related macular degeneration. J. Hum. Genet. *65*, 657–665.

64. Verma, S.S., Cooke Bailey, J.N., Lucas, A., Bradford, Y., Linneman, J.G., Hauser, M.A., Pasquale, L.R., Peissig, P.L., Brilliant, M.H., McCarty, C.A., *et al.* (2016). Epistatic Gene-Based Interaction Analyses for Glaucoma in eMERGE and NEIGHBOR Consortium. PLoS Genet. *12*, e1006186.

65. Winkler, T.W., Grassmann, F., Brandl, C., Kiel, C., Günther, F., Strunz, T., Weidner, L., Zimmermann, M.E., Korb, C.A., Poplawski, A., *et al.* (2020). Genome-wide association meta-analysis for early age-related macular degeneration highlights novel loci and insights for advanced disease. BMC Med. Genomics *13*, 120.

66. Bailey, J.N.C., Loomis, S.J., Kang, J.H., Allingham, R.R., Gharahkhani, P., Khor, C.C., Burdon, K.P., Aschard, H., Chasman, D.I., Igo, R.P., *et al.* (2016). Genome-wide association analysis identifies TXNRD2, ATXN2 and FOXC1 as susceptibility loci for primary open-angle glaucoma. Nat. Genet. *48*, 189–94.

67. Guenther, F., Brandl, C., Winkler, T.W., Wanner, V., Stark, K., Kuechenhoff, H., Heid, I.M. (2020). Chances and challenges of machine learning-based disease classification in genetic association studies illustrated on age-related macular degeneration. Genet. Epidemiol. *44*, 759–777.

68. Zagajewska, K., Piątkowska, M., Goryca, K., Bałabas, A., Kluska, A., Paziewska, A., Pośpiech, E., Grabska-Liberek, I., Hennig, E.E. (2018). GWAS links variants in neuronal development and actin remodeling related loci with pseudoexfoliation syndrome without glaucoma. Exp. Eye Res. *168*, 138–148.

69. Gharahkhani, P., Burdon, K.P., Cooke Bailey, J.N., Hewitt, A.W., Law, M.H., Pasquale, L.R., Kang, J.H., Haines, J.L., Souzeau, E., Zhou, T., *et al.* (2018). Analysis combining correlated glaucoma traits identifies five new risk loci for open-angle glaucoma. Sci. Rep. *8*, 3124.

70. Choquet, H., Paylakhi, S., Kneeland, S.C., Thai, K.K., Hoffmann, T.J., Yin, J., Kvale, M.N., Banda, Y., Tolman, N.G., Williams, P.A., *et al.* (2018). A multiethnic genome-wide association study of primary open-angle glaucoma identifies novel risk loci. Nat. Commun. *9*, 2278.

71. MacGregor, S., Ong, J.-S., An, J., Han, X., Zhou, T., Siggs, O.M., Law, M.H., Souzeau, E., Sharma, S., Lynn, D.J., *et al.* (2018). Genome-wide association study of intraocular pressure uncovers new pathways to glaucoma. Nat. Genet. *50*, 1067–1071.

72. Bonnemaijer, P.W.M., Iglesias, A.I., Nadkarni, G.N., Sanyiwa, A.J., Hassan, H.G., Cook, C., GIGA Study Group, Simcoe, M., Taylor, K.D., Schurmann, C., *et al.* (2018). Genome-wide association study of primary open-angle glaucoma in continental and admixed African populations. Hum. Genet. *137*, 847–862.

73. Zhou, W., Nielsen, J.B., Fritsche, L.G., Dey, R., Gabrielsen, M.E., Wolford, B.N., LeFaive, J., VandeHaar, P., Gagliano, S.A., Gifford, A., *et al.* (2018). Efficiently controlling for case-control imbalance and sample relatedness in large-scale genetic association studies. Nat. Genet. *50*, 1335–1341.

74. Shiga, Y., Akiyama, M., Nishiguchi, K.M., Sato, K., Shimozawa, N., Takahashi, A., Momozawa, Y., Hirata, M., Matsuda, K., Yamaji, T., *et al.* (2018). Genome-wide association study identifies seven novel susceptibility loci for primary open-angle glaucoma. Hum. Mol. Genet. *27*, 1486–1496.

75. Hauser, M.A., Allingham, R.R., Aung, T., Van Der Heide, C.J., Taylor, K.D., Rotter, J.I., Wang, S.-H.J., Bonnemaijer, P.W.M., Williams, S.E., Abdullahi, S.M., *et al.* (2019). Association of Genetic Variants With Primary Open-Angle Glaucoma Among Individuals With African Ancestry. JAMA *322*, 1682–1691.

76. Craig, J.E., Han, X., Qassim, A., Hassall, M., Cooke Bailey, J.N., Kinzy, T.G., Khawaja, A.P., An, J., Marshall, H., Gharahkhani, P., *et al.* (2020). Multitrait analysis of glaucoma identifies new risk loci and enables polygenic prediction of disease susceptibility and progression. Nat. Genet. *52*, 160–166.

77. Kim, Y.W., Kim, Y.J., Cheong, H.S., Shiga, Y., Hashimoto, K., Song, Y.J., Kim, S.H., Choi, H.J., Nishiguchi, K.M., Kawai, Y., *et al.* (2020). Exploring the Novel Susceptibility Gene Variants for Primary Open-Angle Glaucoma in East Asian Cohorts: The GLAU-GENDISK Study. Sci. Rep. *10*, 221.

78. Ishigaki, K., Akiyama, M., Kanai, M., Takahashi, A., Kawakami, E., Sugishita, H., Sakaue, S., Matoba, N., Low, S.-K., Okada, Y., *et al.* (2020). Large-scale genome-wide association study in a Japanese population identifies novel susceptibility loci across different diseases. Nat. Genet. *52*, 669–679.

79. Gharahkhani, P., Jorgenson, E., Hysi, P., Khawaja, A.P., Pendergrass, S., Han, X., Ong, J.S., Hewitt, A.W., Segrè, A. V, Rouhana, J.M., *et al.* (2021). Genome-wide meta-analysis identifies 127 open-angle glaucoma loci with consistent effect across ancestries. Nat. Commun. *12*, 1258.

80. Sakaue, S., Kanai, M., Tanigawa, Y., Karjalainen, J., Kurki, M., Koshiba, S., Narita, A., Konuma, T., Yamamoto, K., Akiyama, M., *et al.* (2021). A cross-population atlas of genetic associations for 220 human phenotypes. Nat. Genet. *53*, 1415–1424.
