# Supplementary material for: Single-cell multiome of the human retina and deep learning nominate causal variants in complex eye diseases
Source: Cell Genom. 2022 Jul 27;2(8):100164. doi: 10.1016/j.xgen.2022.100164 (PMC9584034; doi:10.1016/j.xgen.2022.100164)
Supplement: Document S2. Article plus supplemental information [file mmc10.pdf]

Cell Genomics

Resource

Single-cell multiome of the human retina and deep learning nominate causal variants in complex eye diseases

Graphical abstract

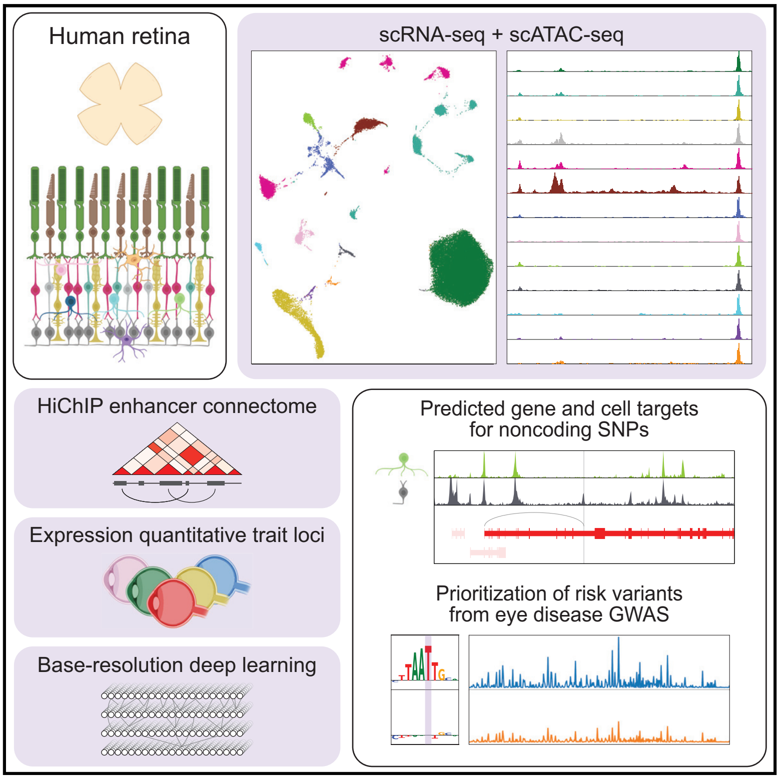

Authors

Sean K. Wang, Surag Nair, Rui Li, ..., Christy Luong, Anshul Kundaje, Howard Y. Chang

Correspondence

howchang@stanford.edu

In brief

Wang et al. present a joint single-cell atlas of gene expression and chromatin accessibility of the adult human retina, which they use to analyze risk variants from genome-wide association studies of five eye diseases. They integrate this atlas with chromatin conformation data, expression quantitative trait loci, and deep learning models to nominate gene and cellular targets in the retina for hundreds of noncoding variants implicated in vision disorders.

Highlights

- Single-cell RNA and chromatin profiling of human retina characterizes 13 cell types
- H3K27ac HiChIP of human retina identifies enhancer-promoter connections
- Deep learning predicts effects of single base changes on chromatin accessibility
- Integrative approach prioritizes noncoding risk variants in complex eye diseases

Wang et al., 2022, Cell Genomics *2*, 100164
August 10, 2022 © 2022 The Author(s).
https://doi.org/10.1016/j.xgen.2022.100164

CellPress

Cell Genomics

CellPress
OPEN ACCESS

Resource

Single-cell multiome of the human retina and deep learning nominate causal variants in complex eye diseases

Sean K. Wang,[1,2] Surag Nair,[3] Rui Li,[1] Katerina Kraft,[1] Anusri Pampari,[3] Aman Patel,[3] Joyce B. Kang,[4,5] Christy Luong,[1,6] Anshul Kundaje,[3,7] and Howard Y. Chang[1,8,9,*]
[1]Center for Personal Dynamic Regulomes, Stanford University, Stanford, CA, USA
[2]Department of Ophthalmology, Stanford University School of Medicine, Stanford, CA, USA
[3]Department of Computer Science, Stanford University, Stanford, CA, USA
[4]Department of Biomedical Informatics, Harvard Medical School, Boston, MA, USA
[5]Center for Data Sciences, Brigham and Women's Hospital, Boston, MA, USA
[6]Department of Chemical and Systems Biology, Stanford University School of Medicine, Stanford, CA, USA
[7]Department of Genetics, Stanford University School of Medicine, Stanford, CA, USA
[8]Howard Hughes Medical Institute, Stanford University, Stanford, CA, USA
[9]Lead contact
*Correspondence: howchang@stanford.edu
https://doi.org/10.1016/j.xgen.2022.100164

SUMMARY

Genome-wide association studies (GWASs) of eye disorders have identified hundreds of genetic variants associated with ocular disease. However, the vast majority of these variants are noncoding, making it challenging to interpret their function. Here we present a joint single-cell atlas of gene expression and chromatin accessibility of the adult human retina with more than 50,000 cells, which we used to analyze single-nucleotide polymorphisms (SNPs) implicated by GWASs of age-related macular degeneration, glaucoma, diabetic retinopathy, myopia, and type 2 macular telangiectasia. We integrate this atlas with a HiChIP enhancer connectome, expression quantitative trait loci (eQTL) data, and base-resolution deep learning models to predict noncoding SNPs with causal roles in eye disease, assess SNP impact on transcription factor binding, and define their known and novel target genes. Our efforts nominate pathogenic SNP-target gene interactions for multiple vision disorders and provide a potentially powerful resource for interpreting noncoding variation in the eye.

INTRODUCTION

Genome-wide association studies (GWASs) of eye disorders, such as glaucoma, myopia, and age-related macular degeneration (AMD), have uncovered hundreds of genetic polymorphisms associated with ocular disease.[1–5] However, the vast majority of variants identified by GWASs reside in noncoding regions of the genome, making it challenging to interpret their function.[6] To better understand how noncoding variants mechanistically contribute to ocular pathology, it would be valuable to map in which cell types their corresponding loci are active. This information would provide novel insights into the cellular biology of genetically complex eye diseases and help nominate specific cell types as targets for therapies.

A recent advance in studying the noncoding genome has been the development of single-cell multiomics technologies, such as paired single-cell RNA sequencing (scRNA-seq) and single-cell assay for transposase-accessible chromatin sequencing (scATAC-seq). While scRNA-seq can classify the different cell types of a tissue based on their transcriptional profiles, its combination with scATAC-seq allows for the additional mapping of cell-type-specific chromatin accessibility. Together, these techniques can reveal the activity of noncoding DNA elements identified by GWASs and have been used to interrogate risk variants for conditions such as Alzheimer disease, Parkinson disease, autism spectrum disorder, and autoimmunity.[7–9]

Investigations of the noncoding genome have likewise benefitted from analytical innovations, such as application of convolutional neural network (CNN)-based deep learning to predict the effects of noncoding polymorphisms.[10–12] Progress in this area has recently led to models with resolution down to a single nucleotide, enabling accurate determination of the critical bases within *cis*-regulatory sequences.[8,12,13] These models offer a validated approach to prioritize noncoding variants with functional relevance and are particularly suitable for tissues in which experimental manipulation is difficult.

Here we generated a joint scRNA- and scATAC-seq atlas of the adult human retina composed of more than 50,000 cells and used it to analyze single-nucleotide polymorphisms (SNPs) implicated by GWAS of five eye diseases: AMD, glaucoma, diabetic retinopathy (DR), myopia, and type 2 macular telangiectasia (MacTel). Layering this atlas with a HiChIP enhancer connectome,[14]

This is an open access article under the CC BY license (http://creativecommons.org/licenses/by/4.0/).

expression quantitative trait locus (eQTL) data,[15] and base-resolution deep learning models,[12] we predicted noncoding SNPs with causal roles in eye disease. Our efforts nominate pathogenic SNP-target gene interactions for multiple vision disorders and provide a potentially powerful resource for interpreting noncoding variation in the eye.

RESULTS

Single-cell multiomics reveal the gene expression and chromatin accessibility landscapes of cell types in the human retina

To generate a single-cell multiome of the human retina, we performed joint scRNA- and scATAC-seq profiling on eight postmortem retinas from four individuals who had no history of eye disease (Table S1). After quality control filtering (Figure S1) and removal of putative doublets (Figures S2A and S2B), we obtained a total of 51,645 human retinal cells in 22 clusters that we assigned to 13 different cell types (Figures 1A, 1B, and S2C). These included abundant cell types, like rod photoreceptors and Müller glia, as well as rarer cell types, such as astrocytes and microglia, which each constituted only 0.4% of profiled cells (Figure 1C; Table S2). Consistent with published scRNA-seq studies of the human retina,[16–19] we observed cell-type-specific expression of many genes, including *PDE6A* in rod photoreceptors, *GRIK1* in OFF-cone bipolar cells, *RLBP1* in Müller glia, *GRM6* in ON-cone and rod bipolar cells, *PRKCA* in rod bipolar cells, *ARR3* in cone photoreceptors, *GAD1* in GABAergic (GABA-) amacrine cells, *ONECUT1* in horizontal cells, *SLC6A9* in AII- and other glycinergic (gly-) amacrine cells, *NEFL* in retinal ganglion cells, *GJD2* in AII-amacrine cells, *GFAP* in astrocytes, and *C1QA* in microglia (Figure 1D and S3A). We also identified a list of candidate marker genes based on differential expression for each of the 13 cell types (Data S1).

Using shared barcodes from joint multiomics profiling, we next assigned scATAC-seq profiles to the 13 cell types characterized by scRNA-seq. The resulting chromatin accessibility profiles clustered by cell type (Figure S4A) and were highly similar to those from a public human retina scATAC-seq dataset (Figure S3B), supporting the validity of our multiome. Peak calling performed on scATAC-seq profiles from each cell type combined into pseudobulk ATAC replicates uncovered a total of 620,386 chromatin accessibility peaks (Figure 2A; Data S2). These scATAC peaks were concordant among donors (Figures S4B and S4C) and included more than 90% of peaks from a published bulk ATAC-seq study of the human retina (Figures 2B),[20] indicating that single-cell multiomics can recapitulate bulk ATAC-seq data. Conversely, more than half of the scATAC peaks were unique to the single-cell dataset (Figure 2B), and nearly 40% of scATAC peaks were accessible in only one cell type (Figure S5A). In line with this, we found 197,826 scATAC marker peaks enriched in a cell-type-specific manner (Figure 2C; Data S3), including many located near cell-type-specific genes (Figure 2D).

With these scATAC peaks, we conducted a motif enrichment analysis to predict which transcription factors (TFs) might be active in each cell type (Figure 3A; Data S4). In accordance with published literature, we observed enrichment of binding motifs for TFs with known cell-type-specific functions, such as OTX2 in photoreceptors and bipolar cells,[21] ONECUT family members in horizontal cells,[22] POU4F family members in retinal ganglion cells,[23] and SPI1 (PU.1) in microglia.[24] For some TFs, cell-type-specific activity was also supported by footprinting analysis of scATAC peaks (Figure 3B), which revealed motif centers to be protected from Tn5 transposition, consistent with TF occupancy. These data offer a cell-type-specific catalog of candidate TFs in the adult retina and may aid our understanding of gene-regulatory networks controlling vision.

Single-cell multiomics uncover the cellular contexts of variants implicated by ocular disease GWASs

With our single-cell multiome, we sought to better understand risk loci identified by GWASs of complex eye disorders. To this end, we compiled a list of 1,331 unique index SNPs from the NHGRI-EBI GWAS Catalog representing GWAS hits for five eye diseases: AMD, glaucoma, DR, myopia, and MacTel (Figure 4A; Table S3).[25] The vast majority (96.5%) of these SNPs localized to noncoding regions of the genome and, thus, could not be interpreted with scRNA-seq data alone. We performed linkage disequilibrium (LD) expansion on all index SNPs to include nearby variants with high probability of coinheritance (LD $R^2 > 0.9$ based on phase 3 genotypes from the 1000 Genomes Project) (Figure S5B).[26] From this, we obtained a total of 7,100 SNPs in loci associated with complex eye disorders, 7,034 (99.1%) of which were noncoding.

We first examined the small number of LD expanded SNPs that encoded an amino acid change or premature stop codon (Data S5). Using our scRNA-seq data, we found that coding variants implicated in AMD and glaucoma were most strongly expressed in microglia and Müller glia, respectively (Figure S6A). As a comparison, we also assessed the cell type expression of mutations known to cause monogenic retinal diseases based on gene annotations from the Retinal Information Network (Figure S6B).[27] Consistent with expectations, genes for primary photoreceptor disorders, such as cone-rod dystrophy and retinitis pigmentosa, were most highly expressed in rods and cones, whereas disease genes for optic atrophy, a condition hallmarked by degeneration of retinal ganglion cells, showed the greatest expression in this cell type.

We next turned to the 7,034 unique SNPs after LD expansion that localized to the noncoding genome (Data S6). To determine in which retinal cell types these SNPs might be active, we overlapped SNP locations with scATAC peaks from our dataset. We found that 1,152 noncoding SNPs (16.4%) overlapped with a scATAC peak (Figure 4B, S5C, and S5D) and that most SNP-containing peaks were present in only one or two cell types (Figure S5E). We then conducted two orthogonal analyses to refine our list of SNPs for those more likely to possess gene regulatory functions. First we identified SNPs in scATAC peaks that were co-accessible with peaks in promoter regions, reasoning that this would select for SNPs in active enhancers. We detected 39,552 such promoter peaks in the human retina, 58.3% of which were co-accessible with at least one scATAC peak (Figure 4C). Leveraging our paired scRNA- and scATAC-seq data, we also searched for SNPs in peaks whose accessibility correlated with the expression of a nearby gene and termed genes that met this criterion "predicted target genes." Using this method, we predicted target genes for

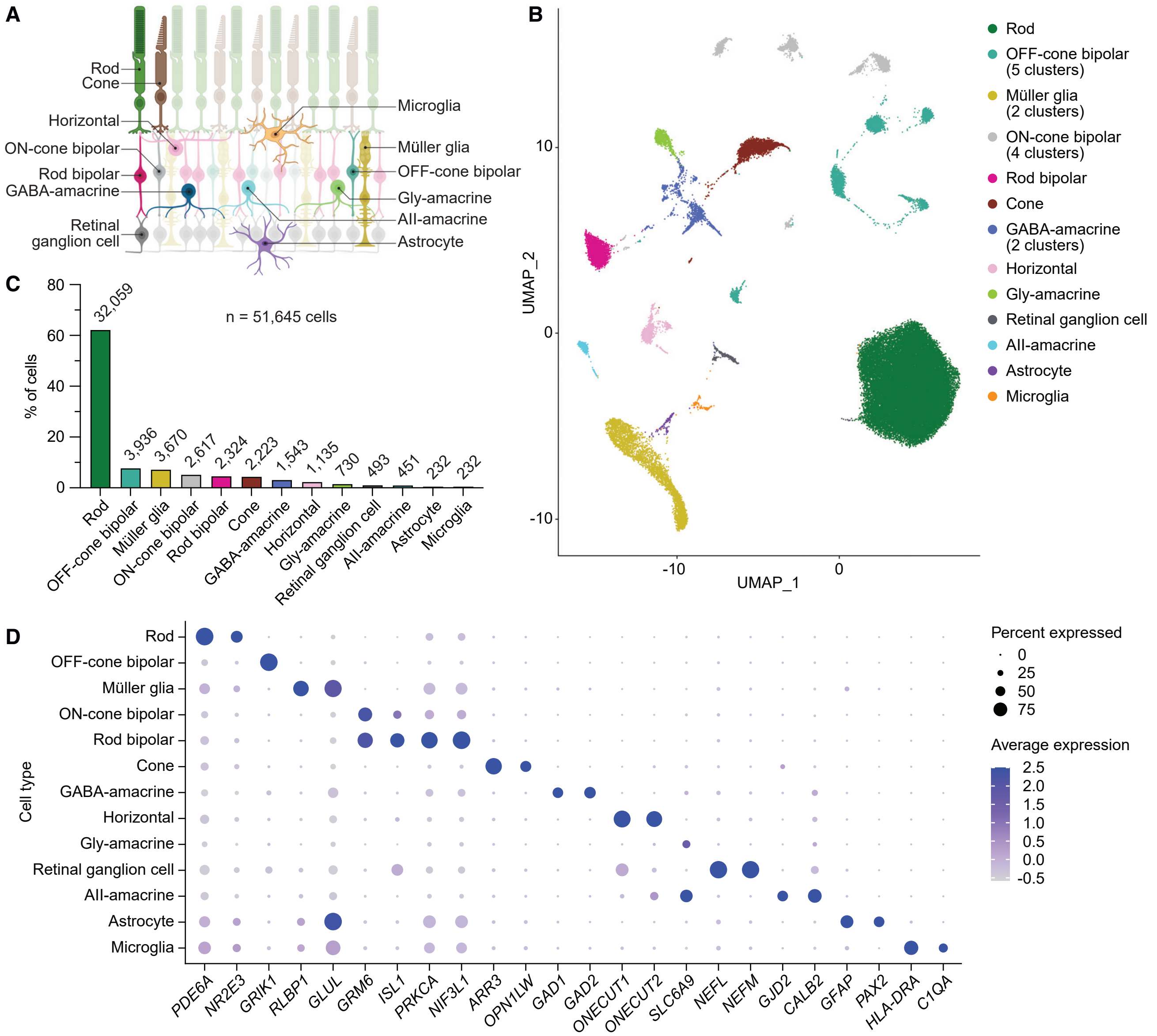

Figure 1. Transcriptional profiles from joint scRNA- and ATAC-seq identify major cell types of the human retina

(A) Schematic of the human retina, depicting the cell types analyzed in this study.

(B) Uniform manifold approximation and projection (UMAP) plot of the 51,645 human retinal cells detected by scRNA-seq after quality control filtering and removal of putative doublets. Eight postmortem retinas from four donors were profiled. A total of 22 clusters were resolved and assigned to 13 cell types.

(C) Frequency of different cell types in the human retina as determined by scRNA-seq. Numbers above each bar denote absolute counts out of 51,645.

(D) Dot plot visualizing the normalized RNA expression of selected marker genes by cell type. The color and size of each dot correspond to the average expression level and fraction of expressing cells, respectively.

199,055 (32.1%) of the 620,386 scATAC peaks in our dataset (Figures 4D and S5F). For nearly half (44.3%) of these peaks, our predictions differed from the nearest gene on the linear genome (Figure S5G), suggesting that noncoding SNPs do not necessarily regulate their nearest gene.

We identified 241 SNPs in scATAC peaks that were co-accessible with promoter peaks and 374 SNPs that had predicted target genes, with 202 SNPs meeting both criteria. As an example, we examined rs4821699 residing in an intron of *TRIOBP* on chromosome 22. This locus has been implicated in glaucoma by multiple GWASs and encodes a protein thought to regulate cytoskeletal organization.[2,28,29] We observed that rs4821699 was most accessible in retinal ganglion cells (Figure 4E), the major cell type that undergoes degeneration during glaucoma. Based on correlations with gene expression, the peak containing this SNP was predicted to target *TRIOBP*. We hypothesize that rs4821699 might therefore play a role in glaucoma by altering *TRIOBP* expression in retinal ganglion cells.

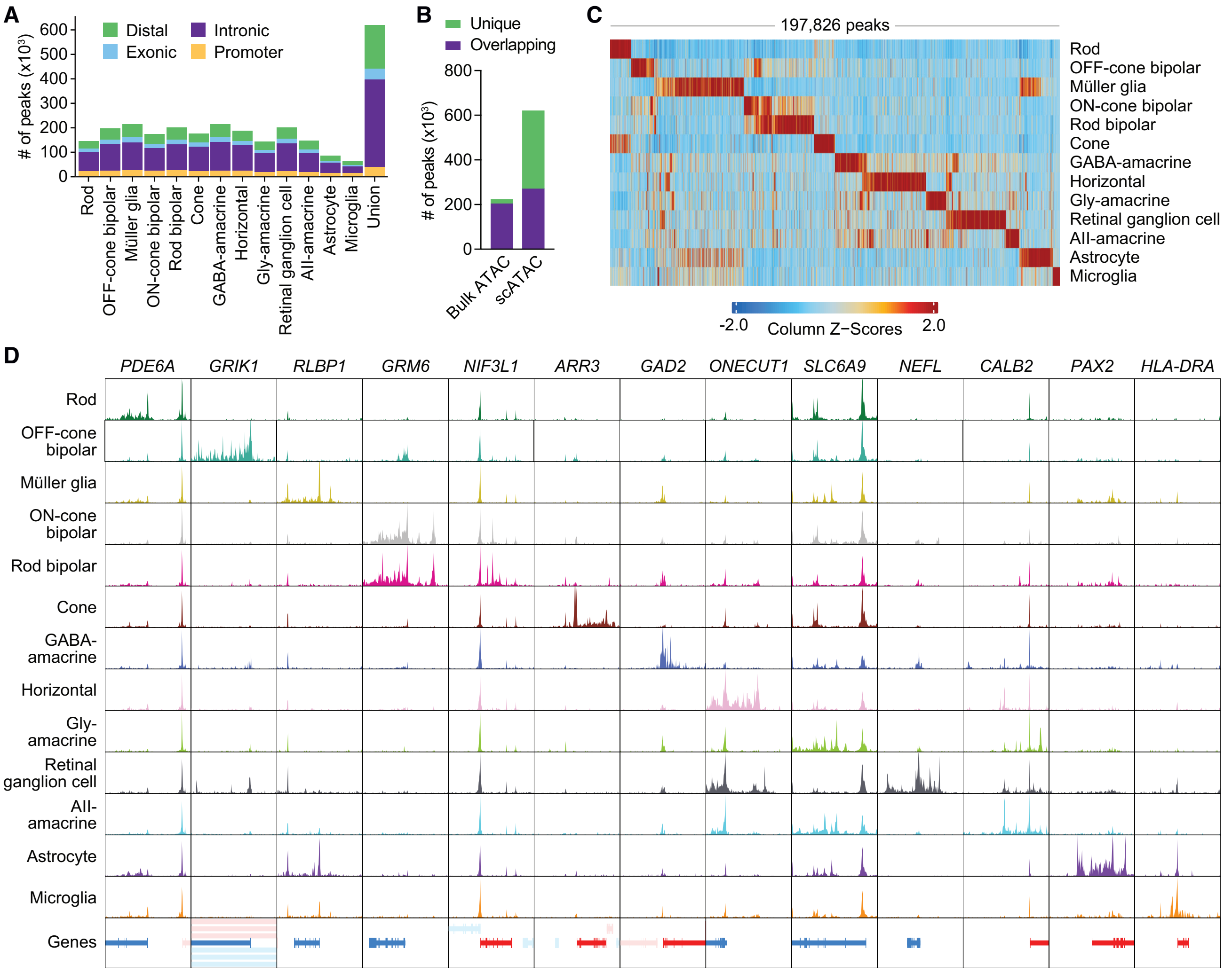

Figure 2. Chromatin accessibility profiles from joint scRNA- and ATAC-seq of the human retina reveal cell-type-specific epigenetic landscapes

(A) Number of chromatin accessibility peaks for each cell type as determined by scATAC-seq. Peaks were required to be present in a least two pseudo-bulk ATAC replicates (n = 2 for astrocyte and microglia, n = 5 for all other cell types).

(B) Overlap of scATAC peaks with peaks from published human retina bulk ATAC-seq data. Overlapping was defined as peaks with any overlapping bases.

(C) Heatmap of scATAC marker peaks enriched in each cell type. Each column represents a marker peak.

(D) Sequencing tracks of chromatin accessibility near selected marker genes by cell type. Each track represents the aggregate scATAC signal of all cells from the given cell type normalized by the total number of reads in TSS regions. Genes in the sense direction (TSS on the left) are shown in red, and genes in the antisense direction (TSS on the right) are shown in blue. Coordinates for each region are as follows: *PDE6A* (chr5:149924792–149964793), *GRIK1* (chr21:29905031–29955033), *RLBP1* (chr15:89201750–89241751), *GRM6* (chr5:178975297–179015298), *NIF3L1* (chr2:200874325–200914327), *ARR3* (chrX:70248304–70288305), *GAD2* (chr10:26186306–26246307), *ONECUT1* (chr15:52781076–52821078), *SLC6A9* (chr1:44005465–44035467), *NEFL* (chr8:24937109–24977110), *CALB2* (chr16:71323711–71368713), *PAX2* (chr10:100715602–100755603), and *HLA-DRA* (chr6:32419841–32459842).

A handful of SNPs associated with eye diseases have been studied experimentally using retinal organoids derived from induced pluripotent stem cells. One such SNP is rs17421627, an index SNP from GWASs of MacTel, representing a T-to-G substitution on chromosome 5.[5,30] We determined rs17421627 to be one of only five SNPs for MacTel with a predicted target gene and found the SNP to be most accessible in Müller glia and astrocytes (Figure 4E). Using linked gene expression data, we also predicted rs17421627 to act on LINC00461, a long noncoding RNA. Consistent with these predictions, deletion of the locus containing rs17421627 in human retinal organoids has been shown to significantly downregulate LINC00461, with the strongest effect in Müller glia.[31] These examples illustrate how single-cell multiomics can reveal the cellular targets of noncoding variants in the retina and show how they might contribute to eye disorders.

Integration of the single-cell multiome with HiChIP and eQTL data validates SNP-target gene predictions

To further prioritize our list of SNPs, we combined our data with two complementary methods for identifying functional SNP-gene

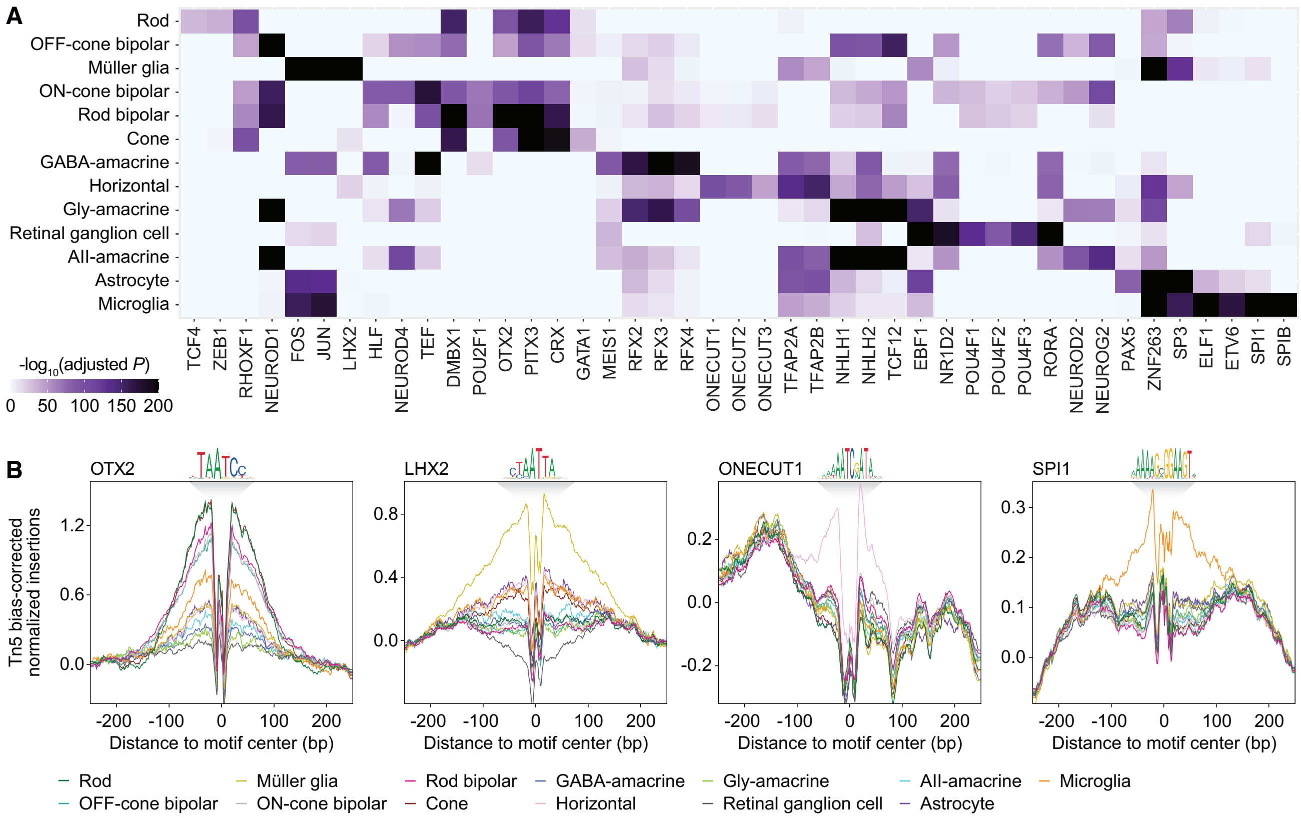

Figure 3. Motif analysis of accessible DNA regions in the human retina predicts cell-type-specific TFs

(A) Heatmap of selected TF binding motifs enriched in each cell type. Darker colors indicate more significant enrichment.

(B) Footprinting analysis of selected TFs across cell types. Footprints were corrected for Tn5 insertion bias by subtracting the Tn5 insertion signal from the footprinting signal.

interactions genome wide. We first performed HiChIP for acetylated histone H3 lysine 27 (H3K27ac), a mark of active enhancers and promoters,[32] to characterize the three-dimensional (3D) enhancer "connectome" of the human retina (Figures S7A and S7B).[14,33] We uncovered 16,692 loop anchors connected by 9,670 HiChIP loops, including several linking regions of chromatin accessibility to the transcription start sites (TSSs) of cell-type-specific genes (Figure S7C; Data S7). Of these loops, more than 95% overlapped with a scATAC peak in both anchors, and more than 99% overlapped with a peak in at least one anchor (Figure 5A). This result shows that accessible chromatin sites identified in scATAC-seq data possess biochemical characteristics of active enhancers and supports their connection to target genes. We also analyzed our list of SNPs using published human retina eQTL data from the Eye Genotype Expression (EyeGEx) database.[15] For more than 90% of SNPs in scATAC peaks, retina eQTL data were available (Figure 5B), enabling genes whose mRNA expression in the human retina changed with specific SNPs to be identified at the bulk tissue level.

We found 187 disease-associated SNPs in scATAC peaks that were linked to a gene by a H3K27ac HiChIP loop. These included rs9966620, the top SNP from a GWAS of DR representing a G-to-A transition in an intron of *TTC39C* on chromosome 18.[34] Using our multiome, we determined that the scATAC peak containing rs9966620 was most accessible in rods (Figure 5C). However, this peak also correlated with the expression of multiple target genes, hampering efforts to interpret how the SNP might function. Incorporating our HiChIP data, we were able to locate a 3D loop connecting rs9966620 with a region 75 kb upstream. This region intersected the TSS of only one gene, *TTC39C-AS1*, suggesting that rs9966620 may modulate DR risk by interacting with *TTC39C-AS1* in rods.

We also detected 596 disease-associated SNPs in scATAC peaks that were significantly associated with a gene by eQTL analysis. One example is rs2730260, an SNP in an intron of *VIPR2* that has been implicated in myopia.[35] This locus encodes one of two known receptors for vasoactive intestinal peptide (VIP), a signaling molecule involved in visual processing.[36] We found that rs2730260 resided in a chromatin accessibility peak specific to Müller glia that again had multiple predicted target genes (Figure 5C). This ambiguity was clarified by retina eQTL data, which showed that variation at rs2730260 significantly correlated with the expression of only *VIPR2* (Figure 5D), supporting this gene as the SNP's primary target. Integration of eQTL data similarly improved our interpretation of rs66475830 on chromosome 6 in the *FRK-NT5DC1-COL10A1* risk locus for AMD.[37,38] This region contains nearly 20 genes within a span of a megabase, making it particularly difficult to functionally annotate GWAS hits. From our single-cell data, we

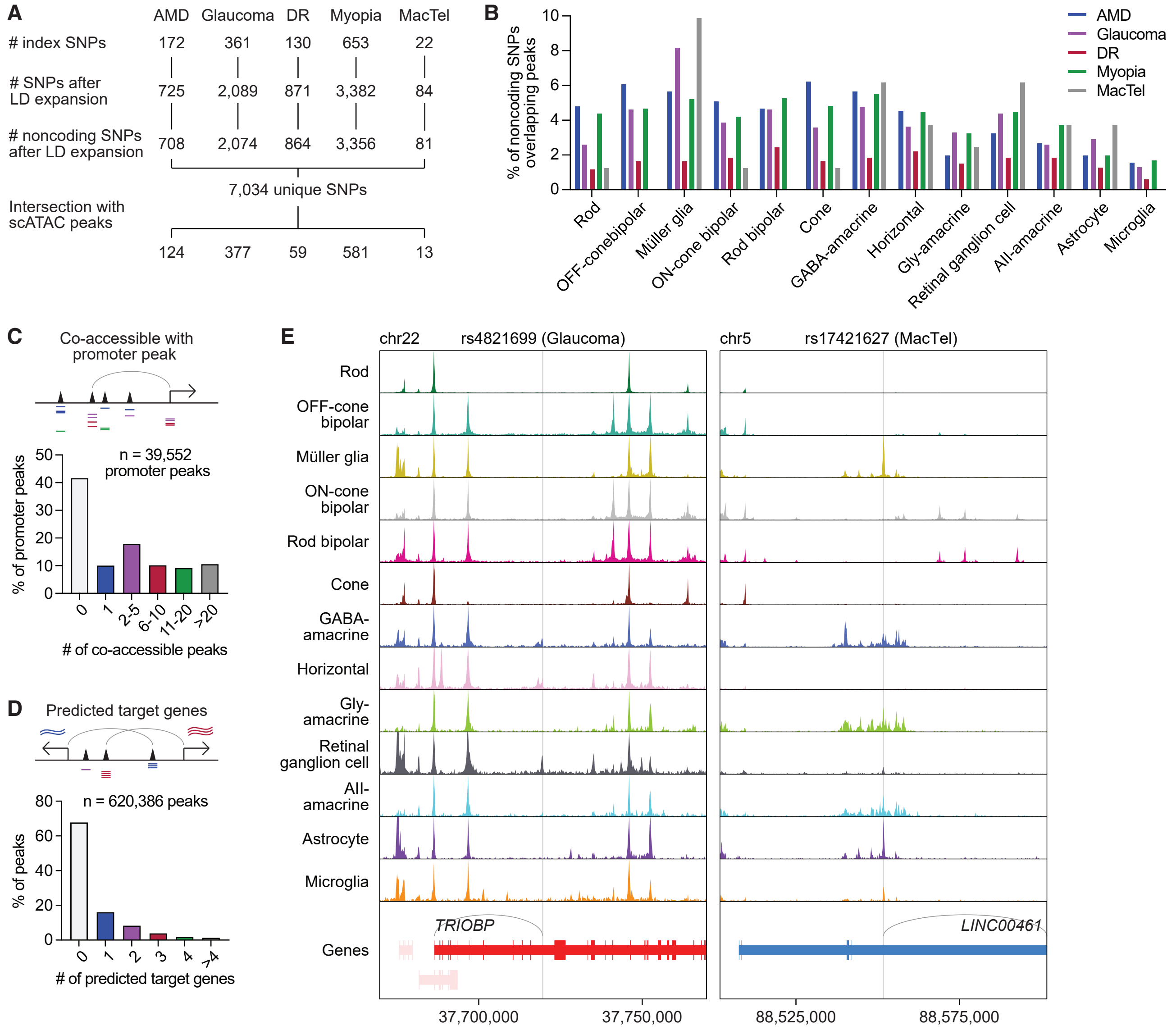

Figure 4. Single-cell multiomics pinpoints the cellular targets of noncoding variants in eye diseases

(A) Overview of SNP selection for interrogating ocular disease GWASs. Index SNPs obtained from GWASs of each disease were subjected to LD expansion, and the resulting noncoding SNPs intersected with scATAC peaks.

(B) Percentage of LD expanded noncoding SNPs from each disease that overlapped with chromatin accessibility peaks for each cell type.

(C) Number of scATAC peaks co-accessible with each promoter peak. Co-accessible was defined as scATAC peaks whose accessibility showed a correlation score greater than 0.3.

(D) Number of predicted target genes for each scATAC peak. Predicted target genes were defined as genes whose RNA expression showed a correlation score >0.3 relative to the accessibility of the tested scATAC peak.

(E) Sequencing tracks of chromatin accessibility near rs4821699 (chr22:37719685) and rs17421627 (chr5:88551768). Genes in the sense and antisense directions are shown in red and blue, respectively. The location of each SNP is depicted by a vertical gray line. Gray arcs indicate predicted target genes for the scATAC peak containing the SNP of interest.

determined rs66475830 to be accessible in amacrine and horizontal cells and predicted *TSPYL1* and *TSPYL4* as target genes (Figure 5C). Retina eQTL analysis revealed that variation at this position was significantly associated with *TSPYL4* expression but not that of other nearby genes (Figure 5D), nominating *TSPYL4* as the effector gene of rs66475830.

Last, we identified many SNP-target gene relationships supported by HiChIP and eQTL data, such as rs77272443 and rs4102217 located in risk loci for myopia and glaucoma, respectively.[39,40] For both of these SNPs, HiChIP and eQTL analyses again refined target gene predictions (Figures S8A and S8B), demonstrating how the combination of single-cell

CellPress
OPEN ACCESS

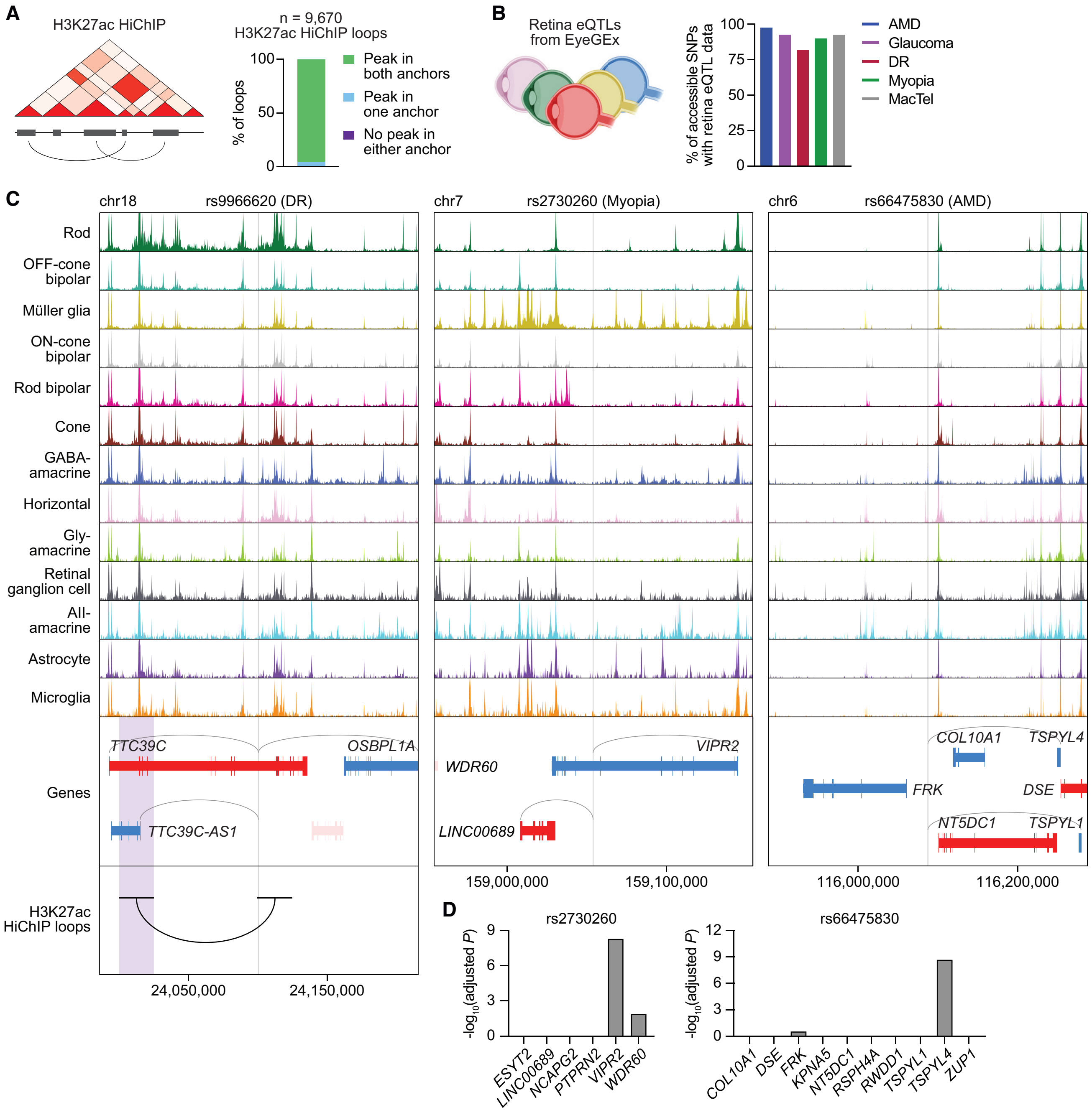

Figure 5. Integration of the single-cell multiome with HiChIP and eQTL data prioritizes functional noncoding polymorphisms in the human retina

(A) Overlap of H3K27ac HiChIP loop anchors (n = 2 biological replicates) with scATAC peaks.

(B) Percentage of SNPs in scATAC peaks for each disease with available retina eQTL data.

(C) Sequencing tracks of chromatin accessibility near rs9966620 (chr18:24100771), rs2730260 (chr7:159054238), and rs66475830 (chr6:116087639). Genes in the sense and antisense directions are shown in red and blue, respectively. The location of each SNP is depicted by a vertical gray line. Gray arcs indicate predicted target genes for the scATAC peak containing the SNP of interest. The black arc overlapping with rs9966620 indicates a H3K27ac HiChIP loop with the region encompassed by the opposite anchor, highlighted in purple.

(D) Significance of SNP-gene associations for rs2730260 or rs66475830 and their nearby genes, as determined by retina eQTL analysis. Adjusted p values for each gene were calculated by multiplying the nominal p value listed in the EyeGEx database by the number of SNP-gene pairs tested for that SNP.

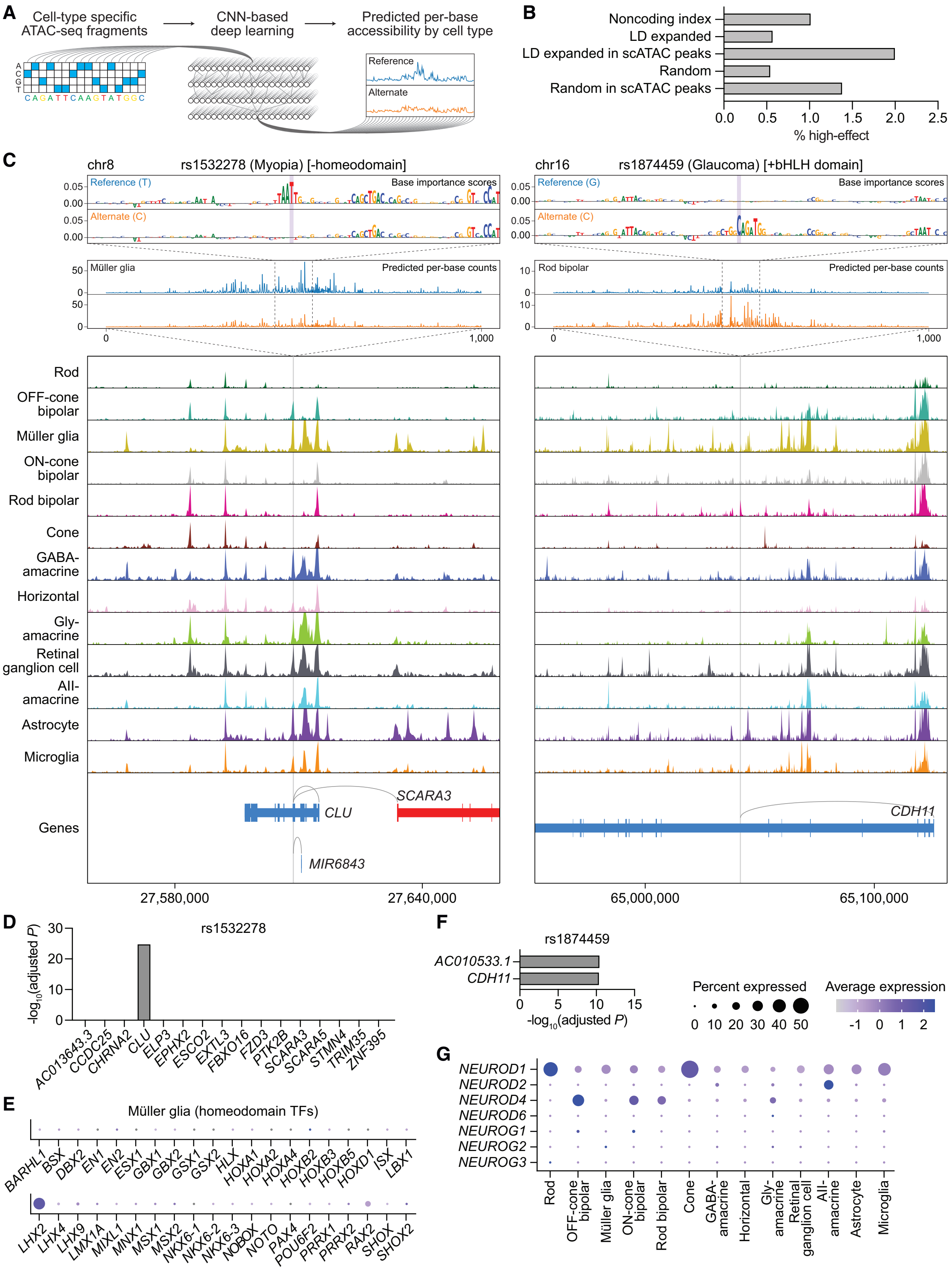

(legend on next page)

multiomics with other assays can enhance interpretation of noncoding variants in eye disease.

Integration of the single-cell multiome with base-resolution deep learning nominates functional mechanisms for disease-associated SNPs

CNN-based deep learning models have proven capable of discerning disease-associated SNPs from other noncoding variants.[8,10,11] As a final method to prioritize SNPs in our dataset, we therefore trained CNNs derived from the BPNet architecture on scATAC-seq profiles for each of the 13 retinal cell types (Figure 6A, S9A, and S9B).[12] At each SNP region, we compared the projected per-base change in chromatin accessibility between reference and alternate alleles using models specific to the different cell types. These calculations allowed us to identify "high-effect" SNPs, which we defined as SNPs predicted to cause a statistically significant (false discovery rate < 0.01) absolute log2 fold change of allele-specific read counts of greater than 0.5 in local chromatin accessibility in any cell type.

We found 23 SNPs (2.0%) residing in scATAC peaks that qualified as high-effect, a greater percentage than among index SNPs, LD expanded SNPs, random SNPs matched for GC content, and random SNPs residing in scATAC peaks (Figure 6B; Data S8). One of the top-scoring SNPs was rs1532278, an index SNP associated with myopia and residing in an intron of *CLU* on chromosome 8.[3] Our atlas predicted rs1532278 to regulate *CLU*, a notion reinforced by eQTL data, and determined the SNP to be accessible in nine of 13 retinal cell types (Figures 6C and 6D). Despite this, base-resolution models projected a T-to-C transition at rs1532278 to alter chromatin accessibility only in Müller glia, specifically by disrupting the motif of a homeodomain TF. Our findings suggest that even though rs1532278 is accessible across multiple cell types, its functional effect in the retina might be restricted to Müller glia because of a cell-type-specific homeodomain TF. We speculate that this TF could be LHX2, given its robust expression in Müller glia by our scRNA-seq data (Figure 6E) as well as data from animal models.[41]

Another high-effect SNP was rs1874459 located in an intron of *CDH11* on chromosome 16, a locus implicated by multiple GWASs for glaucoma.[2,28] Using our multiome, we found rs1874459 to be most accessible in rod bipolar cells and predicted *CDH11* as one of its target genes, an idea supported by eQTL data (Figures 6C and 6F). Incorporating base-resolution models, we then determined that the G-to-C transversion represented by rs1874459 introduced a new basic-helix-loop-helix (bHLH) domain, which was expected to increase accessibility in rod bipolar, OFF-cone bipolar, ON-cone bipolar, gly-amacrine, and AII-amacrine cells. Of the bHLH TFs, members of the neuroD and neurogenin families in particular were predicted by motif analysis to be significantly enriched in these five cell types (Figure 3A; Data S4). We thus compared all neuroD and neurogenin family members using our scRNA-seq data, which revealed only NEUROD4 to be specific to bipolar and amacrine cells (Figure 6G), consistent with its role in specifying these cell types during development.[42,43] Our results suggest that rs1874459 may act on *CDH11* in bipolar and amacrine cells by creating a new bHLH domain recognized by NEUROD4.

DISCUSSION

In this study, we applied single-cell multiomics, HiChIP, eQTL analysis, and base-resolution deep learning to the human retina to decipher the role of noncoding risk variants in five eye diseases. Integrating these methods allowed us to predict gene and cellular targets in the retina for hundreds of SNPs and nominate dozens as potentially pathogenic and meriting functional validation. From an initial list of more than 7,000 noncoding SNPs, we identified 1,152 located in chromatin accessibility peaks. We subsequently focused on SNPs (1) that were co-accessible with a promoter, (2) whose accessibility correlated with the expression of a nearby gene, (3) that were linked to a gene in 3D space by a H3K27ac HiChIP loop, (4) that demonstrated significant association with a gene based on retina eQTL data, and (5) that were predicted to alter local chromatin accessibility as determined by base-resolution models. We propose that SNPs meeting most or all of these criteria (Figure S10; Data S6) be prioritized in future validation efforts.

Our findings build upon recent studies that used primarily fetal tissue and stem-cell-derived organoids to map cell-type-specific chromatin accessibility in the human retina.[31,44] Datasets from these studies are a rich resource for decoding retinal development and understanding congenital eye diseases. However, they might not fully recapitulate the biology of the mature retina, making them potentially less suitable for studying vision

Figure 6. Integration of the single-cell multiome with base-resolution deep learning nominates functional mechanisms for disease-associated SNPs

(A) Schematic of the CNN-based deep learning pipeline.

(B) Percentage of noncoding index SNPs (n = 1,284), LD expanded SNPs (n = 7,034), LD expanded SNPs in scATAC peaks (n = 1,152), randomly selected GC-matched SNPs (n = 9,984), and randomly selected SNPs in scATAC peaks (n = 1,160) that were categorized as high-effect.

(C) Top: predicted per-base accessibility for rs1532278 (chr8:27608798) and rs1874459 (chr16:65041801) in Müller glia and rod bipolar cells, respectively, as determined by deep learning models. A 100-bp window depicts the importance of each base to predicted accessibility at the SNP, and a 1,000-bp window depicts predicted per-base counts for the reference (blue) and alternate (orange) alleles. SNP bases are highlighted in purple. For rs1874459, similar changes in accessibility were predicted for OFF-cone bipolar, ON-cone bipolar, gly-amacrine, and AII-amacrine cells. Bottom: sequencing tracks of chromatin accessibility near rs1532278 and rs1874459. Genes in the sense and antisense directions are shown in red and blue, respectively. The location of each SNP is depicted by a vertical gray line. Gray arcs indicate predicted target genes for the scATAC peak containing the SNP of interest.

(D and F) Significance of SNP-gene associations for rs1532278 (D) or rs1874459 (F) and their nearby genes, as determined by retina eQTL analysis. Adjusted p values for each gene were calculated by multiplying the nominal p value listed in the EyeGEx database by the number of SNP-gene pairs tested for that SNP.

(E) Dot plot visualizing the normalized RNA expression of 40 different homeodomain TFs in Müller glia. The selected TFs correspond to the 40 homeodomain factors whose binding motifs were most significantly enriched in Müller glia, as determined by motif analysis (Data S4).

(G) Dot plot visualizing the normalized RNA expression of neuroD and neurogenin family members by cell type.

disorders that present later in life. Here we generated a single-cell multiome that complements prior datasets by pinpointing cellular targets for disease-associated SNPs in the adult human retina. We combined this multiome with multiple orthogonal analyses to define putative SNP-target gene interactions. By performing base-resolution deep learning, we were able to uncover insights not readily apparent from single-cell, HiChIP, and eQTL data, such as the predicted effect of SNPs on TF binding and the directionality of these effects. To facilitate its use, our atlas is publicly available at https://eyemultiome.su.domains/.

Limitations of the study

Limitations of this study include the relatively low number of cells profiled from rarer cell types like microglia and retinal ganglion cells. Expansion of the current atlas with more cells and donors would be helpful to resolve functionally distinct cell subtypes and could reveal subtype-specific regions of chromatin accessibility that were overlooked here. During SNP prioritization, use of HiChIP and eQTL data from bulk retina may have also favored SNPs from more abundant cell types. Limited overlap of GWAS hits and significant eQTL because of systemic differences in study design may have led to lower concordance among our prioritization criteria.[45]

Finally, it should be noted that the majority of SNPs we examined did not overlap with any chromatin accessibility peaks, suggesting that they were not active in the retina. We hypothesize that many of these unassigned SNPs may instead function in other parts of the eye and thus could not be captured by our analysis. For instance, although the neural retina is damaged in AMD and DR, the retinal pigment epithelium and vasculature, respectively, are thought to be the primary sites of pathology.[46,47] In glaucoma, the trabecular meshwork and ciliary body can modulate disease severity as evidenced by treatments that act on these tissues.[48] Likewise, the choroid and sclera may be involved in myopia, given that they elongate alongside the retina with increasing nearsightedness.[49] Multiomics characterization of these additional ocular regions would enable a more complete understanding of how noncoding SNPs contribute to vision disorders.

STAR★METHODS

Detailed methods are provided in the online version of this paper and include the following:

- KEY RESOURCES TABLE
- RESOURCE AVAILABILITY
  - ○ Lead contact
  - ○ Materials availability
  - ○ Data and code availability
- EXPERIMENTAL MODEL AND SUBJECT DETAILS
- METHOD DETAILS
  - ○ Nuclei isolation
  - ○ scRNA- and scATAC-seq library generation
  - ○ HiChIP library generation
- QUANTIFICATION AND STATISTICAL ANALYSIS
  - ○ scRNA- and scATAC-seq data preprocessing and quality control
  - ○ scRNA-seq data analysis
  - ○ scATAC-seq data analysis
  - ○ Bulk ATAC-seq data analysis
  - ○ Sequencing tracks
  - ○ Motif enrichment analysis
  - ○ SNP selection and LD expansion
  - ○ HiChIP data analysis
  - ○ eQTL analysis
  - ○ Deep learning model training
  - ○ SNP scoring with BPNet

SUPPLEMENTAL INFORMATION

Supplemental information can be found online at https://doi.org/10.1016/j.xgen.2022.100164.

ACKNOWLEDGMENTS

The authors are grateful to Lions VisionGift, Lions Gift of Sight, and the donors who made this study possible. Computing for this project was performed on the Sherlock cluster, a resource provided and maintained by the Stanford Research Computing Center. This work was supported by National Institutes of Health (NIH) grant RM1-HG007735 (to H.Y.C.). S.K.W. is supported by a National Eye Institute training grant (T32EY027816). H.Y.C. is an Investigator of the Howard Hughes Medical Institute.

AUTHOR CONTRIBUTIONS

S.K.W. and H.Y.C. conceived the project and designed experiments. S.K.W., R.L., K.K., and C.L. performed experiments. S.K.W., S.N., A. Pampari, A. Patel, and J.B.K. performed data analyses. A.K. and H.Y.C. supervised the work. S.K.W. and H.Y.C. wrote the manuscript with input from all authors.

DECLARATION OF INTERESTS

H.Y.C. is a co-founder of Accent Therapeutics, Boundless Bio, Cartography Biosciences, and Circ Bio, and an advisor to 10× Genomics, Arsenal Biosciences, and Spring Discovery. A.K. is a co-founder of RavelBio, a consulting fellow with Illumina, and a member of the SAB of OpenTargets, PatchBio, SerImmune, and owns equity in DeepGenomics, Freenome, and ImmunAI.

Received: March 23, 2022
Revised: May 10, 2022
Accepted: July 7, 2022
Published: July 27, 2022

REFERENCES

1. Fritsche, L.G., Igl, W., Bailey, J.N.C., Grassmann, F., Sengupta, S., Bragg-Gresham, J.L., Burdon, K.P., Hebbring, S.J., Wen, C., Gorski, M., et al. (2016). A large genome-wide association study of age-related macular degeneration highlights contributions of rare and common variants. Nat. Genet. *48*, 134–143.
2. MacGregor, S., Ong, J.-S., An, J., Han, X., Zhou, T., Siggs, O.M., Law, M.H., Souzeau, E., Sharma, S., Lynn, D.J., et al. (2018). Genome-wide association study of intraocular pressure uncovers new pathways to glaucoma. Nat. Genet. *50*, 1067–1071.
3. Tedja, M.S., Wojciechowski, R., Hysi, P.G., Eriksson, N., Furlotte, N.A., Verhoeven, V.J.M., Iglesias, A.I., Meester-Smoor, M.A., Tompson, S.W., Fan, Q., et al. (2018). Genome-wide association meta-analysis highlights light-induced signaling as a driver for refractive error. Nat. Genet. *50*, 834–848.
4. Grassi, M.A., Tikhomirov, A., Ramalingam, S., Below, J.E., Cox, N.J., and Nicolae, D.L. (2011). Genome-wide meta-analysis for severe diabetic retinopathy. Hum. Mol. Genet. *20*, 2472–2481.

5. Scerri, T.S., Quaglieri, A., Cai, C., Zernant, J., Matsunami, N., Baird, L., Scheppke, L., Bonelli, R., Yannuzzi, L.A., Friedlander, M., et al. (2017). Genome-wide analyses identify common variants associated with macular telangiectasia type 2. Nat. Genet. *49*, 559–567.
6. Cano-Gamez, E., and Trynka, G. (2020). From GWAS to function: using functional Genomics to identify the mechanisms underlying complex diseases. Front. Genet. *11*, 424.
7. Corces, M.R., Shcherbina, A., Kundu, S., Gloudemans, M.J., Frésard, L., Granja, J.M., Louie, B.H., Eulalio, T., Shams, S., Bagdatli, S.T., et al. (2020). Single-cell epigenomic analyses implicate candidate causal variants at inherited risk loci for Alzheimer's and Parkinson's diseases. Nat. Genet. *52*, 1158–1168.
8. Trevino, A.E., Müller, F., Andersen, J., Sundaram, L., Kathiria, A., Shcherbina, A., Farh, K., Chang, H.Y., Pașca, A.M., Kundaje, A., et al. (2021). Chromatin and gene-regulatory dynamics of the developing human cerebral cortex at single-cell resolution. Cell *184*, 5053–5069.e23.
9. King, H.W., Wells, K.L., Shipony, Z., Kathiria, A.S., Wagar, L.E., Lareau, C., Orban, N., Capasso, R., Davis, M.M., Steinmetz, L.M., et al. (2021). Integrated single-cell transcriptomics and epigenomics reveals strong germinal center-associated etiology of autoimmune risk loci. Sci. Immunol. *6*, eabh3768.
10. Zhou, J., and Troyanskaya, O.G. (2015). Predicting effects of noncoding variants with deep learning-based sequence model. Nat. Methods *12*, 931–934.
11. Kelley, D.R., Snoek, J., and Rinn, J.L. (2016). Basset: learning the regulatory code of the accessible genome with deep convolutional neural networks. Genome Res. *26*, 990–999.
12. Avsec, Ž., Weilert, M., Shrikumar, A., Krueger, S., Alexandari, A., Dalal, K., Fropf, R., McAnany, C., Gagneur, J., Kundaje, A., and Zeitlinger, J. (2021). Base-resolution models of transcription-factor binding reveal soft motif syntax. Nat. Genet. *53*, 354–366.
13. Kim, D.S., Risca, V.I., Reynolds, D.L., Chappell, J., Rubin, A.J., Jung, N., Donohue, L.K.H., Lopez-Pajares, V., Kathiria, A., Shi, M., et al. (2021). The dynamic, combinatorial cis-regulatory lexicon of epidermal differentiation. Nat. Genet. *53*, 1564–1576.
14. Mumbach, M.R., Rubin, A.J., Flynn, R.A., Dai, C., Khavari, P.A., Greenleaf, W.J., and Chang, H.Y. (2016). HiChIP: efficient and sensitive analysis of protein-directed genome architecture. Nat. Methods *13*, 919–922.
15. Ratnapriya, R., Sosina, O.A., Starostik, M.R., Kwicklis, M., Kapphahn, R.J., Fritsche, L.G., Walton, A., Arvanitis, M., Gieser, L., Pietraszkiewicz, A., et al. (2019). Retinal transcriptome and eQTL analyses identify genes associated with age-related macular degeneration. Nat. Genet. *51*, 606–610.
16. Menon, M., Mohammadi, S., Davila-Velderrain, J., Goods, B.A., Cadwell, T.D., Xing, Y., Stemmer-Rachamimov, A., Shalek, A.K., Love, J.C., Kellis, M., and Hafler, B.P. (2019). Single-cell transcriptomic atlas of the human retina identifies cell types associated with age-related macular degeneration. Nat. Commun. *10*, 4902.
17. Lukowski, S.W., Lo, C.Y., Sharov, A.A., Nguyen, Q., Fang, L., Hung, S.S., Zhu, L., Zhang, T., Grünert, U., Nguyen, T., et al. (2019). A single-cell transcriptome atlas of the adult human retina. EMBO J. *38*, e100811.
18. Yan, W., Peng, Y.-R., van Zyl, T., Regev, A., Shekhar, K., Juric, D., and Sanes, J.R. (2020). Cell atlas of the human fovea and peripheral retina. Sci. Rep. *10*, 9802.
19. Lyu, Y., Zauhar, R., Dana, N., Strang, C.E., Hu, J., Wang, K., Liu, S., Pan, N., Gamlin, P., Kimble, J.A., et al. (2021). Implication of specific retinal cell-type involvement and gene expression changes in AMD progression using integrative analysis of single-cell and bulk RNA-seq profiling. Sci. Rep. *11*, 15612.
20. Wang, J., Zibetti, C., Shang, P., Sripathi, S.R., Zhang, P., Cano, M., Hoang, T., Xia, S., Ji, H., Merbs, S.L., et al. (2018). ATAC-Seq analysis reveals a widespread decrease of chromatin accessibility in age-related macular degeneration. Nat. Commun. *9*, 1364.
21. Wang, S., Sengel, C., Emerson, M.M., and Cepko, C.L. (2014). A gene regulatory network controls the binary fate decision of rod and bipolar cells in the vertebrate retina. Dev. Cell *30*, 513–527.
22. Wu, F., Li, R., Umino, Y., Kaczynski, T.J., Sapkota, D., Li, S., Xiang, M., Fliesler, S.J., Sherry, D.M., Gannon, M., et al. (2013). Onecut1 is essential for horizontal cell genesis and retinal integrity. J. Neurosci. *33*, 13053–13065.
23. Gan, L., Xiang, M., Zhou, L., Wagner, D.S., Klein, W.H., and Nathans, J. (1996). POU domain factor Brn-3b is required for the development of a large set of retinal ganglion cells. Proc. Natl. Acad. Sci. USA *93*, 3920–3925.
24. Smith, A.M., Gibbons, H.M., Oldfield, R.L., Bergin, P.M., Mee, E.W., Faull, R.L.M., and Dragunow, M. (2013). The transcription factor PU.1 is critical for viability and function of human brain microglia. Glia *61*, 929–942.
25. Buniello, A., MacArthur, J.A.L., Cerezo, M., Harris, L.W., Hayhurst, J., Malangone, C., McMahon, A., Morales, J., Mountjoy, E., Sollis, E., et al. (2019). The NHGRI-EBI GWAS Catalog of published genome-wide association studies, targeted arrays and summary statistics 2019. Nucleic Acids Res. *47*, D1005–D1012.
26. 1000 Genomes Project Consortium; Abecasis, G.R., Auton, A., Brooks, L.D., DePristo, M.A., Durbin, R.M., Handsaker, R.E., Kang, H.M., Marth, G.T., and McVean, G.A. (2012). An integrated map of genetic variation from 1, 092 human genomes. Nature *491*, 56–65.
27. Daiger, S.P., Sullivan, L.S., and Bowne, S.J. (2013). Genes and mutations causing retinitis pigmentosa. Clin. Genet. *84*, 132–141.
28. Gharahkhani, P., Jorgenson, E., Hysi, P., Khawaja, A.P., Pendergrass, S., Han, X., Ong, J.S., Hewitt, A.W., Segrè, A.V., Rouhana, J.M., et al. (2021). Genome-wide meta-analysis identifies 127 open-angle glaucoma loci with consistent effect across ancestries. Nat. Commun. *12*, 1258.
29. Shahin, H., Walsh, T., Sobe, T., Abu Sa'ed, J., Abu Rayan, A., Lynch, E.D., Lee, M.K., Avraham, K.B., King, M.-C., and Kanaan, M. (2006). Mutations in a novel isoform of TRIOBP that encodes a filamentous-actin binding protein are responsible for DFNB28 recessive nonsyndromic hearing loss. Am. J. Hum. Genet. *78*, 144–152.
30. Bonelli, R., Jackson, V.E., Prasad, A., Munro, J.E., Farashi, S., Heeren, T.F.C., Pontikos, N., Scheppke, L., Friedlander, M., MacTel Consortium, et al. (2021). Identification of genetic factors influencing metabolic dysregulation and retinal support for MacTel, a retinal disorder. Commun. Biol. *4*, 473.
31. Thomas, E.D., Timms, A.E., Giles, S., Harkins-Perry, S., Lyu, P., Hoang, T., Qian, J., Jackson, V.E., Bahlo, M., Blackshaw, S., et al. (2022). Cell-specific cis-regulatory elements and mechanisms of non-coding genetic disease in human retina and retinal organoids. Dev. Cell *57*, 820–836.e6.
32. Creyghton, M.P., Cheng, A.W., Welstead, G.G., Kooistra, T., Carey, B.W., Steine, E.J., Hanna, J., Lodato, M.A., Frampton, G.M., Sharp, P.A., et al. (2010). Histone H3K27ac separates active from poised enhancers and predicts developmental state. Proc. Natl. Acad. Sci. USA *107*, 21931–21936.
33. Mumbach, M.R., Satpathy, A.T., Boyle, E.A., Dai, C., Gowen, B.G., Cho, S.W., Nguyen, M.L., Rubin, A.J., Granja, J.M., Kazane, K.R., et al. (2017). Enhancer connectome in primary human cells identifies target genes of disease-associated DNA elements. Nat. Genet. *49*, 1602–1612.
34. Meng, W., Chan, B.W., Ezeonwumelu, C., Hébert, H.L., Campbell, A., Soler, V., and Palmer, C.N. (2019). A genome-wide association study implicates that the TTC39C gene is associated with diabetic maculopathy with decreased visual acuity. Ophthalmic Genet. *40*, 252–258.
35. Shi, Y., Gong, B., Chen, L., Zuo, X., Liu, X., Tam, P.O.S., Zhou, X., Zhao, P., Lu, F., Qu, J., et al. (2013). A genome-wide meta-analysis identifies two novel loci associated with high myopia in the Han Chinese population. Hum. Mol. Genet. *22*, 2325–2333.
36. Park, S.J.H., Borghuis, B.G., Rahmani, P., Zeng, Q., Kim, I.-J., and Demb, J.B. (2015). Function and circuitry of VIP+ interneurons in the mouse retina. J. Neurosci. *35*, 10685–10700.

37. Yu, Y., Bhangale, T.R., Fagerness, J., Ripke, S., Thorleifsson, G., Tan, P.L., Souied, E.H., Richardson, A.J., Merriam, J.E., Buitendijk, G.H.S., et al. (2011). Common variants near FRK/COL10A1 and VEGFA are associated with advanced age-related macular degeneration. Hum. Mol. Genet. *20*, 3699–3709.

38. Fritsche, L.G., Chen, W., Schu, M., Yaspan, B.L., Yu, Y., Thorleifsson, G., Zack, D.J., Arakawa, S., Cipriani, V., Ripke, S., et al. (2013). Seven new loci associated with age-related macular degeneration. Nat. Genet. *45*, 433–439.e1-2.

39. Tideman, J.W.L., Pärssinen, O., Haarman, A.E.G., Khawaja, A.P., Wedenoja, J., Williams, K.M., Biino, G., Ding, X., Kähönen, M., Lehtimäki, T., et al. (2021). Evaluation of shared genetic susceptibility to high and low myopia and hyperopia. JAMA Ophthalmol. *139*, 601–609.

40. Craig, J.E., Han, X., Qassim, A., Hassall, M., Cooke Bailey, J.N., Kinzy, T.G., Khawaja, A.P., An, J., Marshall, H., Gharahkhani, P., et al. (2020). Multitrait analysis of glaucoma identifies new risk loci and enables polygenic prediction of disease susceptibility and progression. Nat. Genet. *52*, 160–166.

41. de Melo, J., Miki, K., Rattner, A., Smallwood, P., Zibetti, C., Hirokawa, K., Monuki, E.S., Campochiaro, P.A., and Blackshaw, S. (2012). Injury-independent induction of reactive gliosis in retina by loss of function of the LIM homeodomain transcription factor Lhx2. Proc. Natl. Acad. Sci. USA *109*, 4657–4662.

42. Hatakeyama, J., Tomita, K., Inoue, T., and Kageyama, R. (2001). Roles of homeobox and bHLH genes in specification of a retinal cell type. Development *128*, 1313–1322.

43. Inoue, T., Hojo, M., Bessho, Y., Tano, Y., Lee, J.E., and Kageyama, R. (2002). Math3 and NeuroD regulate amacrine cell fate specification in the retina. Development *129*, 831–842.

44. Finkbeiner, C., Ortuño-Lizarán, I., Sridhar, A., Hooper, M., Petter, S., and Reh, T.A. (2022). Single-cell ATAC-seq of fetal human retina and stem-cell-derived retinal organoids shows changing chromatin landscapes during cell fate acquisition. Cell Rep. *38*, 110294.

45. Mostafavi, H., Spence, J.P., Naqvi, S., and Pritchard, J.K. (2022). Limited overlap of eQTLs and GWAS hits due to systematic differences in discovery. Preprint at bioRxiv. https://doi.org/10.1101/2022.05.07.491045.

46. Fleckenstein, M., Keenan, T.D.L., Guymer, R.H., Chakravarthy, U., Schmitz-Valckenberg, S., Klaver, C.C., Wong, W.T., and Chew, E.Y. (2021). Age-related macular degeneration. Nat. Rev. Dis. Prim. *7*, 31.

47. Duh, E.J., Sun, J.K., and Stitt, A.W. (2017). Diabetic retinopathy: current understanding, mechanisms, and treatment strategies. JCI Insight *2*, 93751.

48. Weinreb, R.N., Aung, T., and Medeiros, F.A. (2014). The pathophysiology and treatment of glaucoma: a review. JAMA *311*, 1901–1911.

49. Morgan, I.G., Ohno-Matsui, K., and Saw, S.-M. (2012). Myopia. Lancet *379*, 1739–1748.

50. Corces, M.R., Trevino, A.E., Hamilton, E.G., Greenside, P.G., Sinnott-Armstrong, N.A., Vesuna, S., Satpathy, A.T., Rubin, A.J., Montine, K.S., Wu, B., et al. (2017). An improved ATAC-seq protocol reduces background and enables interrogation of frozen tissues. Nat. Methods *14*, 959–962.

51. Tan, L. (2021). Determining the 3D genome structure of a single mammalian cell with Dip-C. STAR Protoc. *2*, 100622.

52. Granja, J.M., Corces, M.R., Pierce, S.E., Bagdatli, S.T., Choudhry, H., Chang, H.Y., and Greenleaf, W.J. (2021). ArchR is a scalable software package for integrative single-cell chromatin accessibility analysis. Nat. Genet. *53*, 403–411.

53. Stuart, T., Butler, A., Hoffman, P., Hafemeister, C., Papalexi, E., Mauck, W.M., Hao, Y., Stoeckius, M., Smibert, P., and Satija, R. (2019). Comprehensive integration of single-cell data. Cell *177*, 1888–1902.e21.

54. Korsunsky, I., Millard, N., Fan, J., Slowikowski, K., Zhang, F., Wei, K., Baglaenko, Y., Brenner, M., Loh, P.-R., and Raychaudhuri, S. (2019). Fast, sensitive and accurate integration of single-cell data with Harmony. Nat. Methods *16*, 1289–1296.

55. Feng, J., Liu, T., Qin, B., Zhang, Y., and Liu, X.S. (2012). Identifying ChIP-seq enrichment using MACS. Nat. Protoc. *7*, 1728–1740.

56. Amemiya, H.M., Kundaje, A., and Boyle, A.P. (2019). The ENCODE blacklist: identification of problematic regions of the genome. Sci. Rep. *9*, 9354.

57. Quinlan, A.R., and Hall, I.M. (2010). BEDTools: a flexible suite of utilities for comparing genomic features. Bioinformatics *26*, 841–842.

58. Langmead, B., and Salzberg, S.L. (2012). Fast gapped-read alignment with Bowtie 2. Nat. Methods *9*, 357–359.

59. Li, D., Hsu, S., Purushotham, D., Sears, R.L., and Wang, T. (2019). WashU Epigenome browser update 2019. Nucleic Acids Res. *47*, W158–W165.

60. Weirauch, M.T., Yang, A., Albu, M., Cote, A.G., Montenegro-Montero, A., Drewe, P., Najafabadi, H.S., Lambert, S.A., Mann, I., Cook, K., et al. (2014). Determination and inference of eukaryotic transcription factor sequence specificity. Cell *158*, 1431–1443.

61. Myers, T.A., Chanock, S.J., and Machiela, M.J. (2020). LDlinkR: an R package for rapidly calculating linkage disequilibrium statistics in diverse populations. Front. Genet. *11*, 157.

62. Sherry, S.T., Ward, M.H., Kholodov, M., Baker, J., Phan, L., Smigielski, E.M., and Sirotkin, K. (2001). dbSNP: the NCBI database of genetic variation. Nucleic Acids Res. *29*, 308–311.

63. Servant, N., Varoquaux, N., Lajoie, B.R., Viara, E., Chen, C.-J., Vert, J.-P., Heard, E., Dekker, J., and Barillot, E. (2015). HiC-Pro: an optimized and flexible pipeline for Hi-C data processing. Genome Biol. *16*, 259.

64. Rao, S.S.P., Huntley, M.H., Durand, N.C., Stamenova, E.K., Bochkov, I.D., Robinson, J.T., Sanborn, A.L., Machol, I., Omer, A.D., Lander, E.S., and Aiden, E.L. (2014). A 3D map of the human genome at kilobase resolution reveals principles of chromatin looping. Cell *159*, 1665–1680.

65. Durand, N.C., Robinson, J.T., Shamim, M.S., Machol, I., Mesirov, J.P., Lander, E.S., and Aiden, E.L. (2016). Juicebox provides a visualization system for hi-C contact maps with unlimited zoom. Cell Syst. *3*, 99–101.

66. Tareen, A., and Kinney, J.B. (2020). Logomaker: beautiful sequence logos in Python. Bioinformatics *36*, 2272–2274.

STAR★METHODS

KEY RESOURCES TABLE

REAGENT or RESOURCE	SOURCE	IDENTIFIER
**Antibodies**		
Anti-histone H3 (acetyl K27) antibody	Abcam	Cat# ab4729; RRID: AB_2118291
**Biological samples**		
Frozen adult postmortem human retinas	Lions VisionGift, Lions Gift of Sight	N/A
**Chemicals, peptides, and recombinant proteins**		
IGEPAL CA-630	Sigma-Aldrich	Cat# I8896
RNase inhibitor	New England BioLabs	Cat# M0314
Protease inhibitor	Roche	Cat# 11697498001
Iodixanol	Sigma-Aldrich	Cat# D1556
Tween-20	Roche	Cat# 11332465001
Bovine serum albumin	Miltenyi Biotec	Cat# 130-091-376
Digitonin	Thermo Fischer Scientific	Cat# BN2006
Glycerol	Ricca Chemical	Cat# 3290-16
Paraformaldehyde	Electron Microscopy Sciences	Cat# 50-980-487
Sodium dodecyl sulfate	Invitrogen	Cat# 15553027
Triton X-100	Sigma-Aldrich	Cat# 93443
MboI	New England BioLabs	Cat# R0147
Biotin-14-dATP	Jena Bioscience	Cat# NU-835-BIO14
Klenow fragment	New England BioLabs	Cat# M0210
T4 DNA ligase	New England BioLabs	Cat# M0202
Dimethylformamide	Sigma-Aldrich	Cat# 227056
Sodium deoxycholate	Bioworld	Cat# 40430018
**Critical commercial assays**		
Single Cell Multiome ATAC + Gene Expression kit	10x Genomics	Cat# 1000285
**Deposited data**		
scRNA-seq, scATAC-seq, and HiChIP data	This paper	Gene Expression Omnibus: GSE196235
BPNet models	This paper	Zenodo: https://doi.org/10.5281/zenodo.6330053
**Software and algorithms**		
Cell Ranger-ARC	10x Genomics	v2.0.0
ArchR	Granja et al., 2021	v1.01
Seurat	Stuart et al., 2019	v3.1.5
Harmony	Korsunsky et al., 2019	v1.0
MACS2	Feng et al., 2012	v2.1.1
BEDTools	Quinlan et al., 2010	v2.30.0
LDlinkR	Myers et al., 2020	v1.2.0
HiC-Pro	Servant et al., 2015	v2.11.0
Juicer	Rao et al., 2014	v1.6
Juicebox	Durand et al., 2016	v1.11.08
Logomaker	Tareen et al., 2020	v0.8
**Other**		
Dounce homogenizers	Sigma-Aldrich	Cat# D8938
SPRIselect beads	Beckman Coulter	Cat# B23317
Protein A Dynabeads	Invitrogen	Cat# 10001D
Streptavidin Dynabeads	Invitrogen	Cat# 65001

RESOURCE AVAILABILITY

Lead contact

Further information and requests for resources and reagents should be directed to and will be fulfilled by the lead contact, Dr. Howard Y. Chang (howchang@stanford.edu).

Materials availability

Due to the limiting nature of primary samples, human tissues used in this study are not available upon request. This study did not involve any other unique materials.

Data and code availability

Raw and processed scRNA-seq, scATAC-seq, and HiChIP data from this study have been uploaded to Gene Expression Omnibus under the accession number GSE196235. A web page summarizing these data is also available at https://eyemultiome.su.domains/. Code used for scRNA- and scATAC-seq analysis is available at https://doi.org/10.5281/zenodo.6795162. Code used for BPNet model training is available at http://doi.org/10.5281/zenodo.6796067. BPNet models are available at https://doi.org/10.5281/zenodo.6330053.

EXPERIMENTAL MODEL AND SUBJECT DETAILS

Postmortem adult human retinas were procured from consented donors by Lions VisionGift (Portland, OR, USA) or Lions Gift of Sight (St Paul, MN, USA) under protocols approved by the Eye Bank Association of America. None of the donors had a history of ocular disease. De-identified retinas were flash-frozen in liquid nitrogen with a maximum death-to-preservation interval of 12 hours and shipped to Stanford University for processing. Donor information is listed in Table S1.

METHOD DETAILS

Nuclei isolation

Nuclei were isolated from frozen retinas using the Omni-ATAC protocol (https://doi.org/10.17504/protocols.io.6t8herw).[50] Briefly, tissues were Dounce homogenized in cold homogenization buffer containing 0.3% IGEPAL CA-630 in the presence of protease and RNase inhibitors to release nuclei from frozen cells. Nuclei were subsequently purified via iodixanol gradient centrifugation and washed with ATAC resuspension buffer containing RNase inhibitor and 0.1% Tween-20 before permeabilization following the 10x Genomics demonstrated protocol for complex tissues (CG000375, Rev. B). After resuspension in diluted nuclei buffer, nuclei were counted using a manual hemocytometer to achieve a targeted nuclei recovery of 10,000 nuclei per sample.

scRNA- and scATAC-seq library generation

Joint scRNA- and scATAC-seq libraries were prepared using the 10x Genomics Single Cell Multiome ATAC + Gene Expression kit according to manufacturer's instructions. Libraries were sequenced with paired-end 150-bp reads on an Illumina NovaSeq 6000 to a target depth of 250 million read pairs per sample.

HiChIP library generation

H3K27ac HiChIP libraries were prepared as previously reported with minor modifications.[14] Briefly, following isolation of nuclei from frozen retinas as described above, ~8 million nuclei from each sample were washed with nuclei isolation buffer from the diploid chromatin conformation capture (Dip-C) protocol and fixed with 2% paraformaldehyde at room temperature for 10 minutes.[51] Fixed nuclei were then washed twice with cold 1% bovine serum albumin in phosphate-buffered saline before resuspension in 0.5% sodium dodecyl sulfate and resumption of the published HiChIP protocol. Digestion was performed using the MboI restriction enzyme, and sonication was conducted using a Covaris E220 with 5 duty cycles, peak incident power of 140, and 200 cycles per burst for 4 minutes. The ab4729 ChIP validated antibody from Abcam was used to target H3K27ac. HiChIP libraries were sequenced with paired-end 75-bp reads on either an Illumina HiSeq 400 or Illumina NextSeq 550.

QUANTIFICATION AND STATISTICAL ANALYSIS

scRNA- and scATAC-seq data preprocessing and quality control

Demultiplexed scRNA- and scATAC-seq fastq files were inputted into the Cell Ranger ARC pipeline (version 2.0.0) from 10x Genomics to generate barcoded count matrices of gene expression and ATAC data. For each sample, count matrices were loaded in ArchR and selected for barcodes that appeared in both the scRNA-seq and scATAC-seq datasets.[52] Samples in ArchR were quality control filtered for nuclei with 200-50,000 RNA transcripts, <1% mitochondrial reads, <5% ribosomal reads, TSS enrichment >6, and >2,500 ATAC fragments. Quality control filtered nuclei subsequently underwent automated removal of doublets using the filterDoublets function in ArchR, which identifies and removes the nearest neighbors of simulated doublets.[52]

scRNA-seq data analysis

scRNA-seq data from nuclei remaining after quality control filtering and automated removal of doublets were analyzed using Seurat (version 3.1.5).[53] After merging all preprocessed samples into a single Seurat object, gene expression counts were normalized using the NormalizeData function, scaled using the ScaleData function, and batch corrected using Harmony.[54] Graph-based clustering was then performed on the Harmony-corrected data using the top 20 principal components at a resolution of 0.5. Cluster identities were manually annotated based on the expression of genes from published scRNA-seq studies of the human retina.[16–18] Marker genes for each cluster were additionally identified using the FindAllMarkers function with a minimum fraction of 0.5 and a log2 fold change of 1 (Data S1). Clusters expressing canonical marker genes from different cell types were designated as putative doublets and excluded, after which re-clustering was performed using the same parameters. Clusters with no detected marker genes were also excluded, after which the dataset was also re-clustered. Clusters in the final dataset representing subpopulations of the same cell type were grouped together for downstream analyses.

scATAC-seq data analysis

scATAC-seq data were analyzed using ArchR (version 1.0.1) based on barcoded cell type identities from scRNA-seq.[52] For each cell type, pseudo-bulk ATAC replicates were created using the addGroupCoverages function with default parameters, which generated between two to five replicates depending on how many cells of that type were present in each sample. Chromatin accessibility peaks on chromosomes 1-22 and X and outside of blacklist regions were then called using the addReproduciblePeakSet function and MACS2,[55,56] with scATAC peaks for each cell type defined as those present in at least two pseudo-bulk ATAC replicates (Data S2). Marker peaks were identified using the getMarkerFeatures function with a log2 fold change ≥ 1 and false discovery rate ≤ 0.01 as determined by Wilcoxon pairwise comparisons (Data S3). Promoter peaks were defined as scATAC peaks within 2,000 bp upstream or 100 bp downstream of a TSS, and peaks co-accessible to promoter peaks were identified using the getCoAccessibility function with a correlation cutoff of 0.3 and resolution of 1. Predicted target genes for each scATAC peak were generated using the getPeak2GeneLinks function integrating barcode-matched RNA expression data from scRNA-seq with a correlation cutoff of 0.3 and resolution of 1. Nearest genes were determined using the BEDTools closest function based on gene annotations from TxDb.Hsapiens.UCSC.hg38.knownGene.[57]

Bulk ATAC-seq data analysis

Bulk ATAC-seq analysis was performed on published ATAC-seq data from five healthy human retinas.[20] After adapter trimming, fastq files were mapped to the hg38 genome using Bowtie2 and filtered to remove PCR duplicates and retain reads from only chromosomes 1-22 and X.[58] Peak calling was then conducted individually on each sample using MACS2,[55] followed by exclusion of peaks in blacklist regions.[56] Peak calls present in at least two of the five retinas were included in the bulk ATAC-seq peak set.

Sequencing tracks

Sequencing tracks of chromatin accessibility were generated in ArchR using the plotBrowserTrack function and were normalized by the total number of reads in TSS regions.[52] In some cases, bigWig files generated in ArchR using the getGroupBW function were visualized on the WashU Epigenome Browser.[59] All data were aligned and annotated to the hg38 reference genome unless otherwise stated.

Motif enrichment analysis

TF motif enrichment analysis was performed on scATAC peaks using the peakAnnoEnrichment function in ArchR with default parameters based on position frequency matrices from Cis-BP (Data S4).[52,60] Footprinting analysis of TFs was conducted using the getFootprints function in ArchR.[52] To correct for Tn5 insertion bias, the Tn5 insertion signal was subtracted from footprinting signals prior to plotting.

SNP selection and LD expansion

Index SNPs implicated in AMD, glaucoma, DR, myopia, or MacTel and located on chromosomes 1-22 and X were collected from the NHGRI-EBI GWAS Catalog, a curated collection of human GWAS.[25] LD expansion was then performed using LDlinkR to add any SNPs in LD with each index SNP,[61] defined as a LD R^2 value >0.9 in the phase 3 genotypes of the 1000 Genomes Project.[26] LD expanded SNPs were filtered to exclude variants in coding regions based on annotations in dbSNP to obtain the final set of noncoding SNPs (Data S6).[62] A list of all GWAS used in this study is provided in Table S3.

HiChIP data analysis

HiChIP sequencing files were initially processed using the HiC-Pro pipeline (version 2.11.0) to remove duplicate reads, assign reads to MboI restriction fragments, filter for valid interactions, and generate binned interaction matrices.[63] Filtered read pairs from HiC-Pro were subsequently converted into .hic files and inputted into HiCCUPS from the Juicer pipeline to call loops (Data S7).[64] HiChIP interaction maps depicting all valid interactions identified by HiC-Pro were visualized using Juicebox.[65]

eQTL analysis

Retina eQTL data were obtained from the Eye Genotype Expression (EyeGEx) database.[15] Each of the 1,152 SNPs overlapping with a scATAC peak was searched in the database and the nominal p value of any gene associations with that SNP noted. Adjusted p values were calculated by multiplying the nominal p value by the number of SNP-gene pairs tested for that SNP. Interactions with an adjusted p value < 0.05 were considered significant.

Deep learning model training

scATAC-seq reads from the Cell Ranger ARC pipeline were aggregated by cell type to generate cell type-specific fragments files. The fragments files were converted to BigWig tracks of base-resolution Tn5 insertion sites with an +4/-4 shift to account for Tn5 shift. For each cell type, in addition to the peak regions, we selected an equal number of non-peak regions that were matched for GC content in their peaks. We then trained cell type-specific BPNet models to predict the log counts and base-resolution Tn5 insertion profiles as previously reported.[8,12] Briefly, the BPNet model takes as input a 2,114 bp one-hot encoded input sequence and predicts the ATAC-seq profile and log counts in a 1,000 bp window centered at the input sequence. Following BPNet formulation, we used a multinomial negative log likelihood (MNLL) for the profile output of the model and a mean square error (MSE) loss for the log counts output of the model. The relative loss weight used for the counts loss was 0.1 times the mean total counts per region. During each epoch, training examples were jittered by up to 500 bp on either side and a random half of the sequences were reverse complemented. Each batch contained a 10:1 ratio of peaks to non-peak regions. Five models were trained for each cell type corresponding to five disjoint training folds. Model training was performed using Keras/Tensorflow 2.

SNP scoring with BPNet

To score LD expanded SNPs associated with eye disease, we centered the input window at the SNP and obtained the log2 fold change in predicted counts between the reference and alternate alleles for each cell type-specific model. We averaged the log2 fold change over the five model folds for each SNP and cell type. To obtain p values, we performed one-sided Poisson tests of the predicted alternate allele count with the rate parameter set to the predicted reference allele count (counts averaged over five folds). For each SNP, we combined p values across cell types with Fisher's method and performed Benjamini-Hochberg correction. SNPs with an absolute fold-averaged log2 fold change >0.5 and false discovery rate <0.01 were assigned putative "high effect" annotation. To obtain a background set, random noncoding SNPs were chosen by shuffling a list of all SNPs from the 1000 Genomes Project,[26] filtering out coding regions, and selecting the first 10,000 entries. Only random SNPs localized to chromosomes 1-22 and X were then retained, leaving 9,984 background SNPs. Background SNPs had similar GC content as disease-associated LD expanded SNPs (51% versus 52%) and were scored as described above. Base importance tracks were visualized using Logomaker.[66]

Cell Genomics, Volume *2*

Supplemental information

Single-cell multiome of the human retina and deep learning nominate causal variants in complex eye diseases

Sean K. Wang, Surag Nair, Rui Li, Katerina Kraft, Anusri Pampari, Aman Patel, Joyce B. Kang, Christy Luong, Anshul Kundaje, and Howard Y. Chang

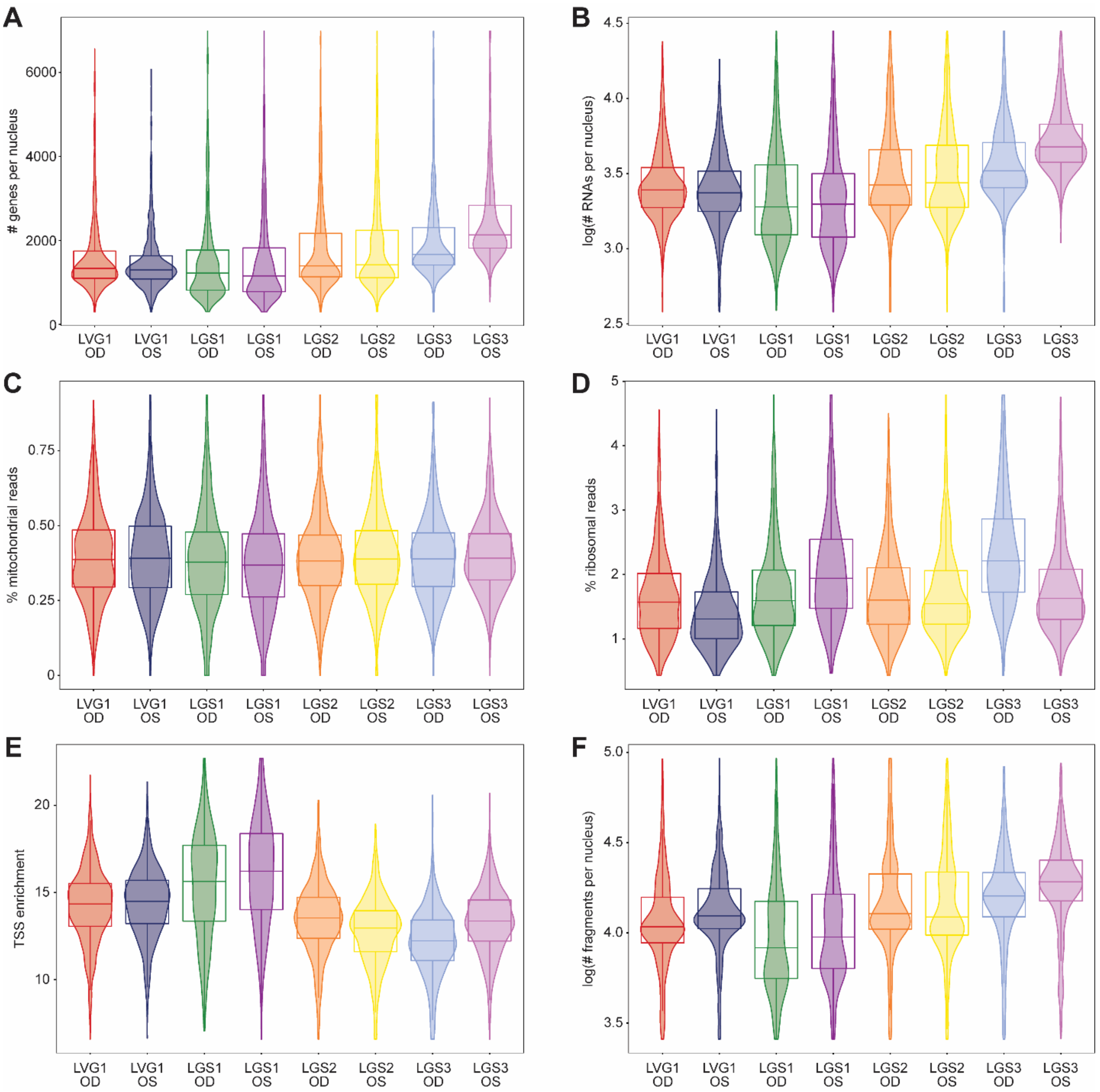

Figure S1. Single-cell RNA- and ATAC-seq quality control metrics, Related to STAR Methods.

(A-F) Violin plots depicting the number of detected genes per nucleus (A), number of detected RNA transcripts per nucleus (B), percentage of mitochondrial reads (C), percentage of ribosomal reads (D), TSS enrichment (E), and number of detected fragments per nucleus (F) by retina in the final dataset. Boxes depict the 25th percentile, median, and 75th percentile of each metric.

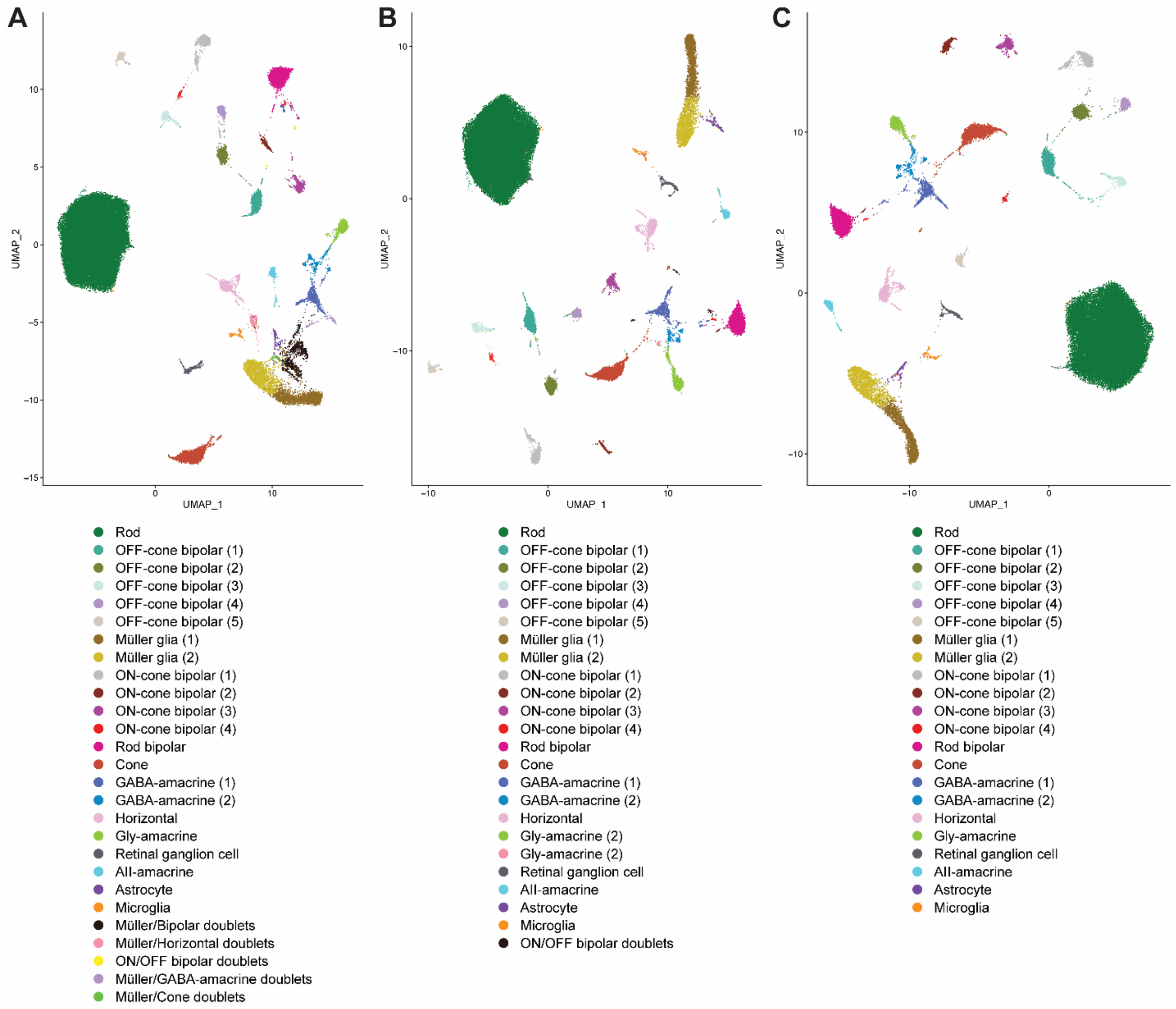

Figure S2. Single-cell RNA-seq cluster assignments, Related to Figure 1.

(A) First iteration uniform manifold approximation and projection (UMAP) plot of the scRNA-seq dataset after quality control filtering, automated removal of doublets, and exclusion of clusters with no detected marker genes. Five clusters comprised of putative doublets were subsequently removed and the dataset re-clustered. **(B)** Second iteration UMAP of the scRNA-seq dataset. One cluster comprised of putative doublets was subsequently removed and the dataset re-clustered. **(C)** Final iteration UMAP of the scRNA-seq dataset. Clusters representing subpopulations of the same cell type were grouped together for downstream analyses.

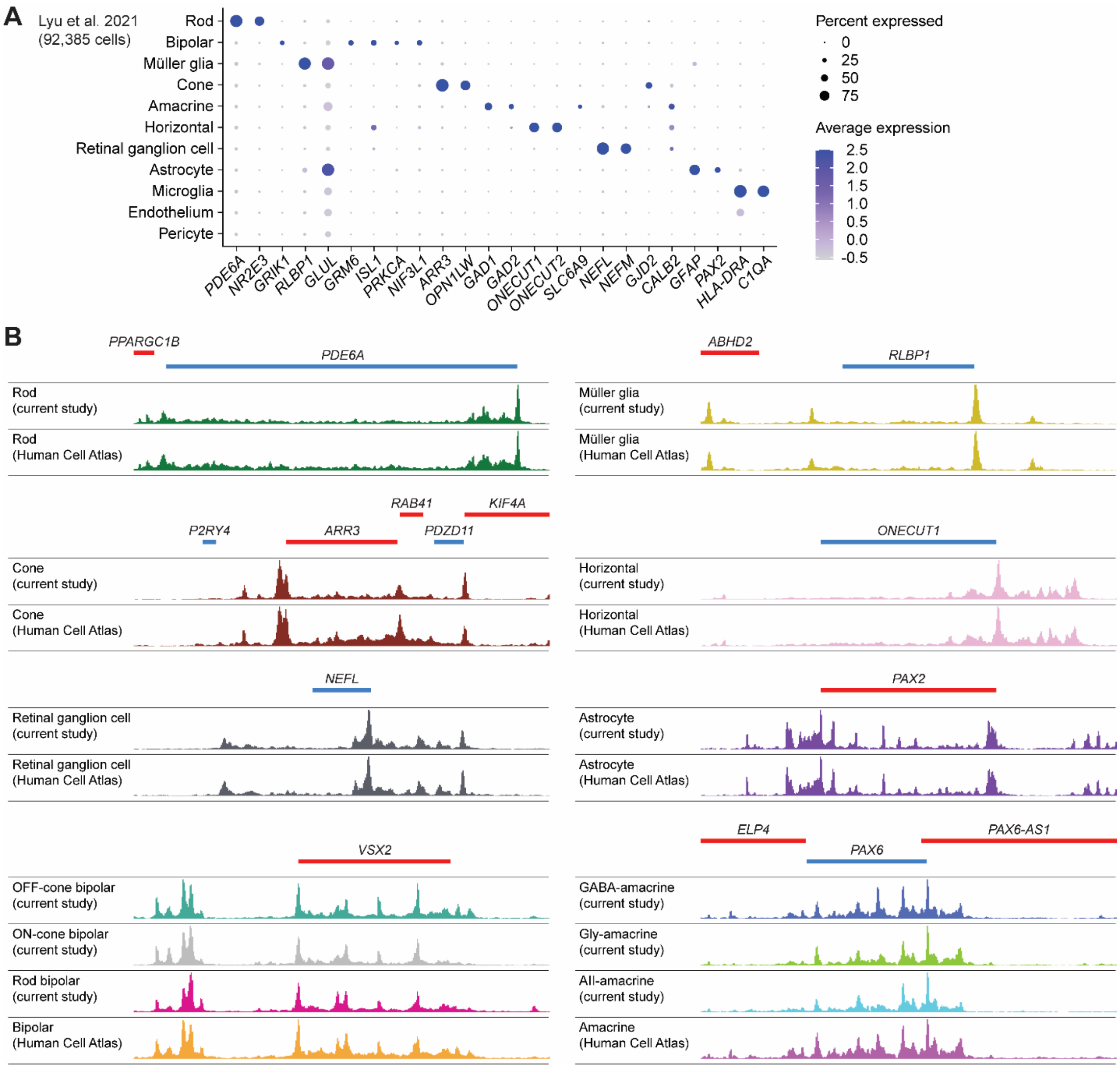

Figure S3. Comparison of single-cell multiome with public datasets, Related to Figures 1 and 2.

(**A**) Dot plot visualizing the normalized RNA expression of the same marker genes from Figure 1D in an adult human retina scRNA-seq dataset from Lyu et al.[1] (**B**) Sequencing tracks of cell type-specific chromatin accessibility near selected marker genes from the current study or an adult human retina scATAC-seq dataset from the Human Cell Atlas (https://retina-atac.cells.ucsc.edu). Tracks from the Human Cell Atlas were aligned to the hg19 genome and matched to hg38 coordinates for visualization.

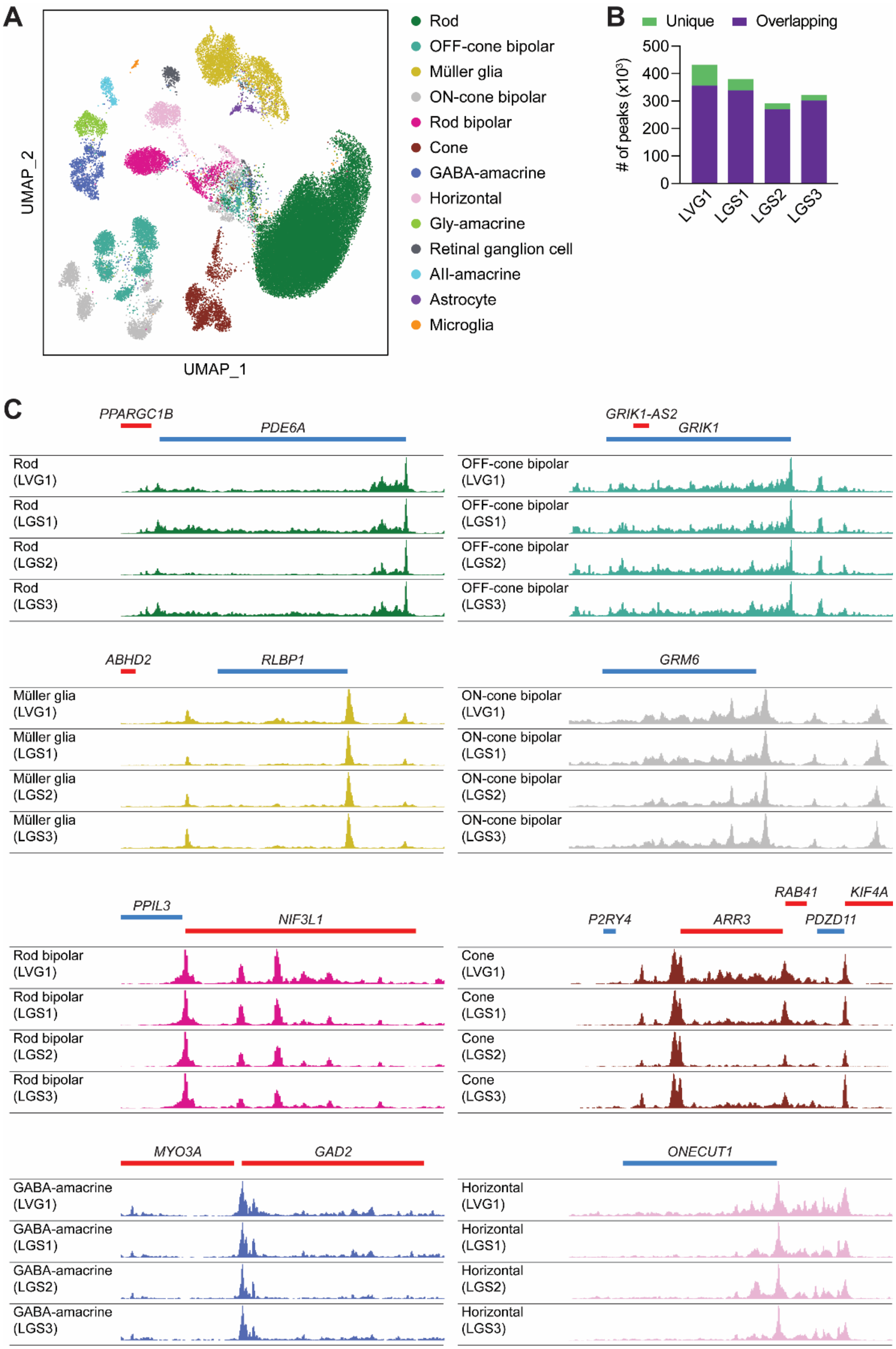

Figure S4. Validation of scATAC-seq data across donors, Related to Figure 2.

(**A**) UMAP plot of the 51,645 human retinal cells based on scATAC-seq profiles. Cell identities were determined by scRNA-seq. (**B**) Overlap of scATAC peaks identified by independent

analysis of each donor. Overlapping was defined as having any overlapping bases with a peak from another donor. (**C**) Sequencing tracks of cell type-specific chromatin accessibility near selected marker genes by donor. Tracks were generated for cell types with >200 cells per donor using non-batch-corrected scATAC data.

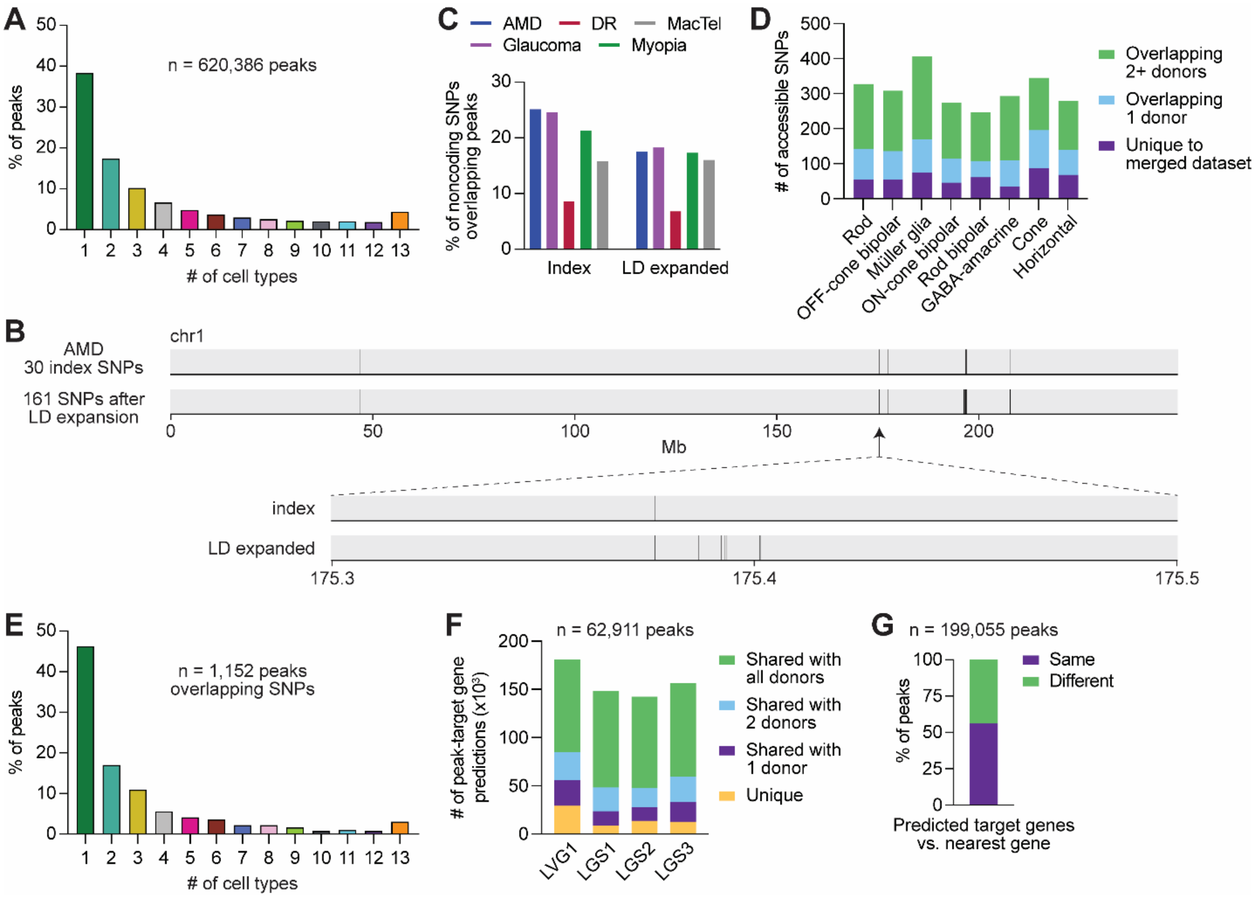

Figure S5. Characterization of chromatin accessibility peaks and linkage disequilibrium expanded SNPs, Related to Figures 2 and 4.

(A) Number of cell types exhibiting each of the 620,386 scATAC peaks. **(B)** Visual depiction of LD expansion for the 30 index SNPs on chromosome 1 associated with AMD. Each vertical black line represents a SNP. **(C)** Percentage of noncoding index and LD expanded SNPs from each disease that overlapped with at least one scATAC peak. **(D)** Number of accessible SNPs from the merged dataset that overlapped with donor-specific scATAC peaks in the indicated cell types. Analysis was restricted to cell types with >200 cells in each of the four donors. (**E**) Number of cell types exhibiting each of the 1,152 scATAC peaks overlapping with a LD expanded SNP. (**F**) Comparison of peak-target gene predictions by donor for the 62,911 scATAC peaks that were accessible in all donors and had at least one predicted target gene. (**G**) Comparison of predicted target genes with the nearest gene for each chromatin accessibility peak with at least one predicted target gene. Same was defined as any of the predicted target genes being the nearest gene.

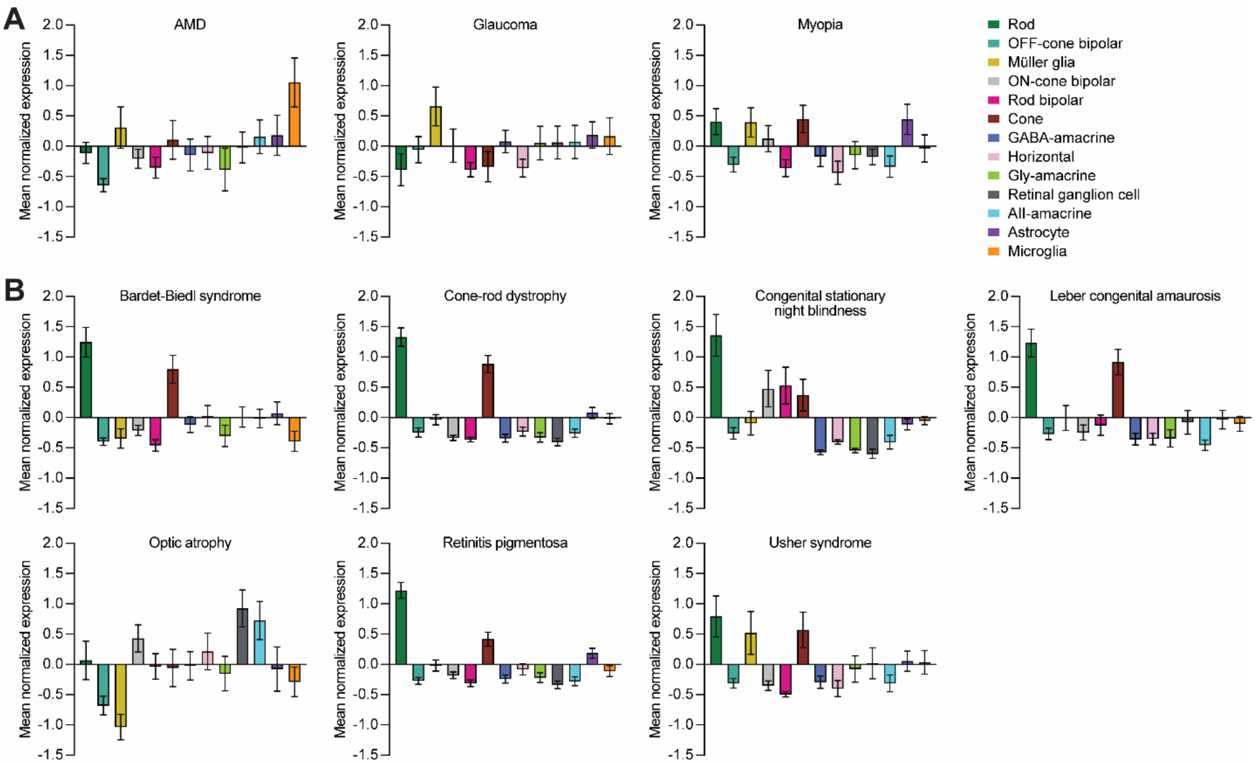

Figure S6. Cell type expression of coding variants in complex and monogenic eye diseases, Related to Figure 4.

(**A**, **B**) Mean normalized RNA expression by cell type of genes containing coding variants implicated in complex eye diseases (A) or monogenic retinal diseases (B). Coding variants were defined as variants resulting in an amino acid change or premature stop codon. Gene expression analysis was restricted to diseases with ten or more implicated genes. Monogenic retinal disease genes were obtained from the Retinal Information Network (https://sph.uth.edu/retnet).[2] Genes containing coding variants for each disease are listed in Data S5. Error bars denote standard error of the mean.

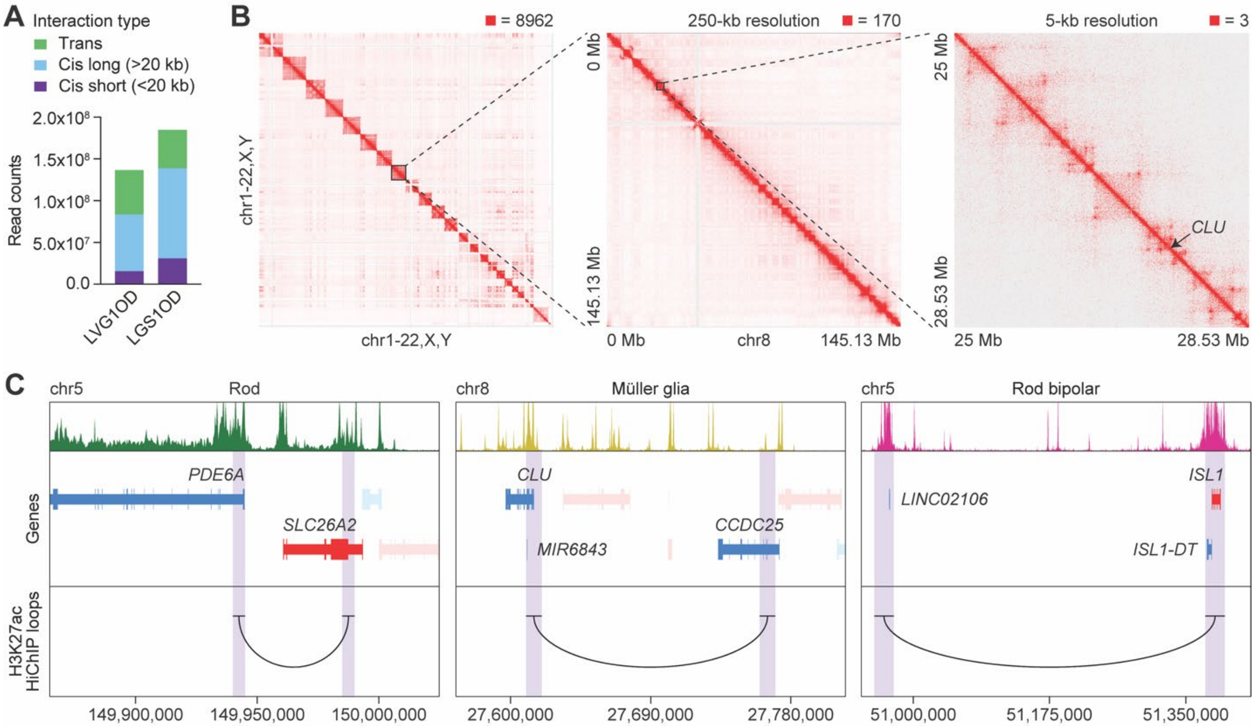

Figure S7. H3K27ac HiChIP predicts enhancer-promoter interactions in the human retina, Related to Figure 5.

(A) HiChIP interaction types by sample. Cis refers to interactions on the same chromosome, while trans refers to interactions spanning separate chromosomes. **(B)** HiChIP interaction maps at whole genome, 250-kb, and 5-kb resolution. Sample shown is LGS1OD. Signal was normalized to the square root of coverage. Numbers above the interaction maps indicate maximum signal in each matrix. **(C)** Sequencing tracks of cell type chromatin accessibility and H3K27ac HiChIP loops overlapping with the TSS of selected marker genes. Genes in the sense and antisense directions are shown in red and blue, respectively. Regions encompassed by the loop anchors are highlighted in purple.

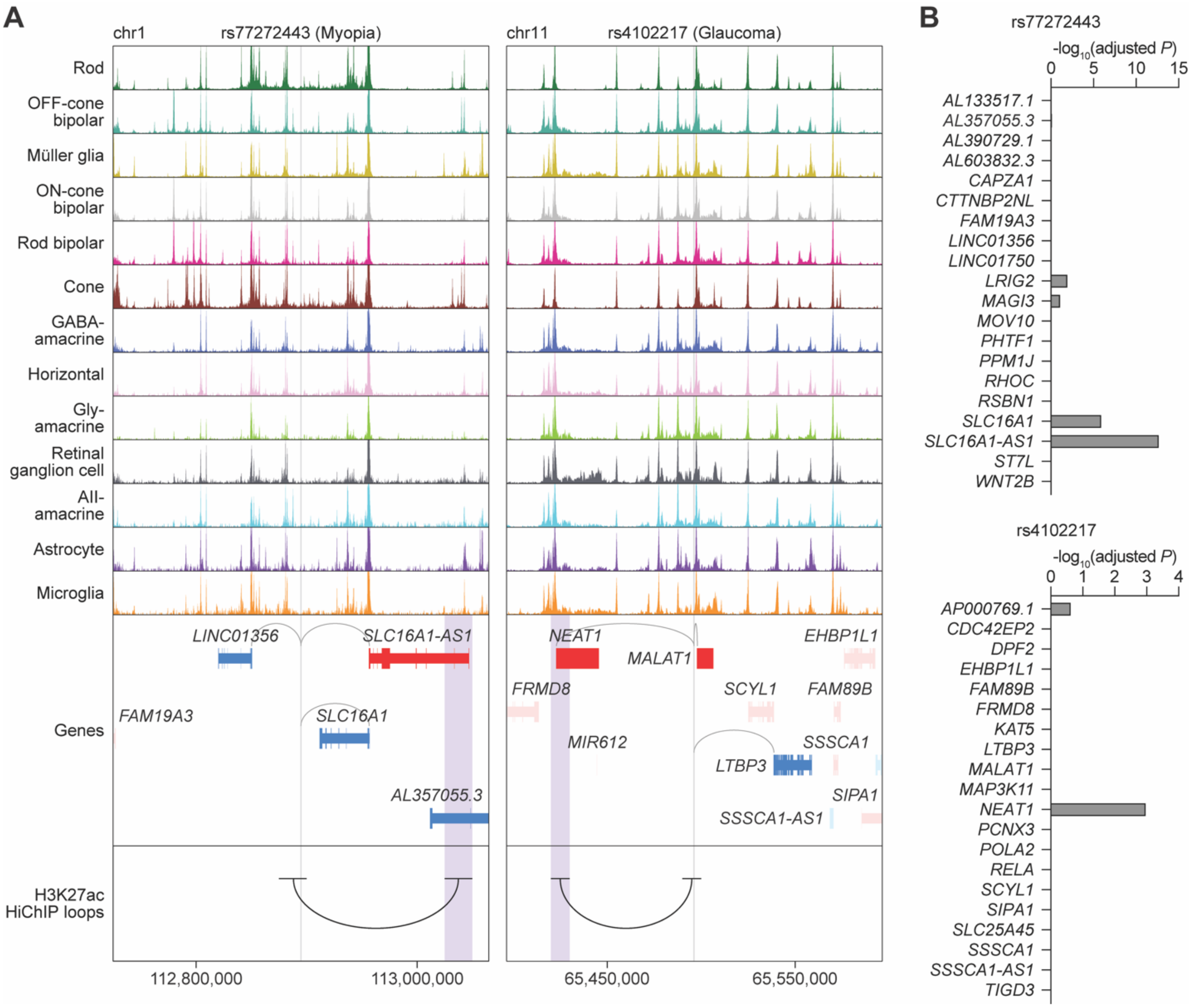

Figure S8. Integration of single-cell multiome with HiChIP and eQTL data refines SNP-target gene predictions, Related to Figure 5.

(A) Sequencing tracks of chromatin accessibility near rs77272443 (chr1:112894884) and rs4102217 (chr11:65496424). Genes in the sense and antisense directions are shown in red and blue, respectively. The location of each SNP is depicted by a vertical gray line. Gray arcs indicate predicted target genes for the scATAC peak containing the SNP of interest. Black arcs overlapping with SNPs indicate H3K27ac HiChIP loops with the regions encompassed by the opposite anchors highlighted in purple. **(B)** Significance of SNP-gene associations for rs77272443 or rs4102217 and their 20 nearest genes as determined by retina eQTL analysis. Adjusted P values for each gene were calculated by multiplying the nominal P value listed in the EyeGEx database by the number of SNP-gene pairs tested for that SNP.

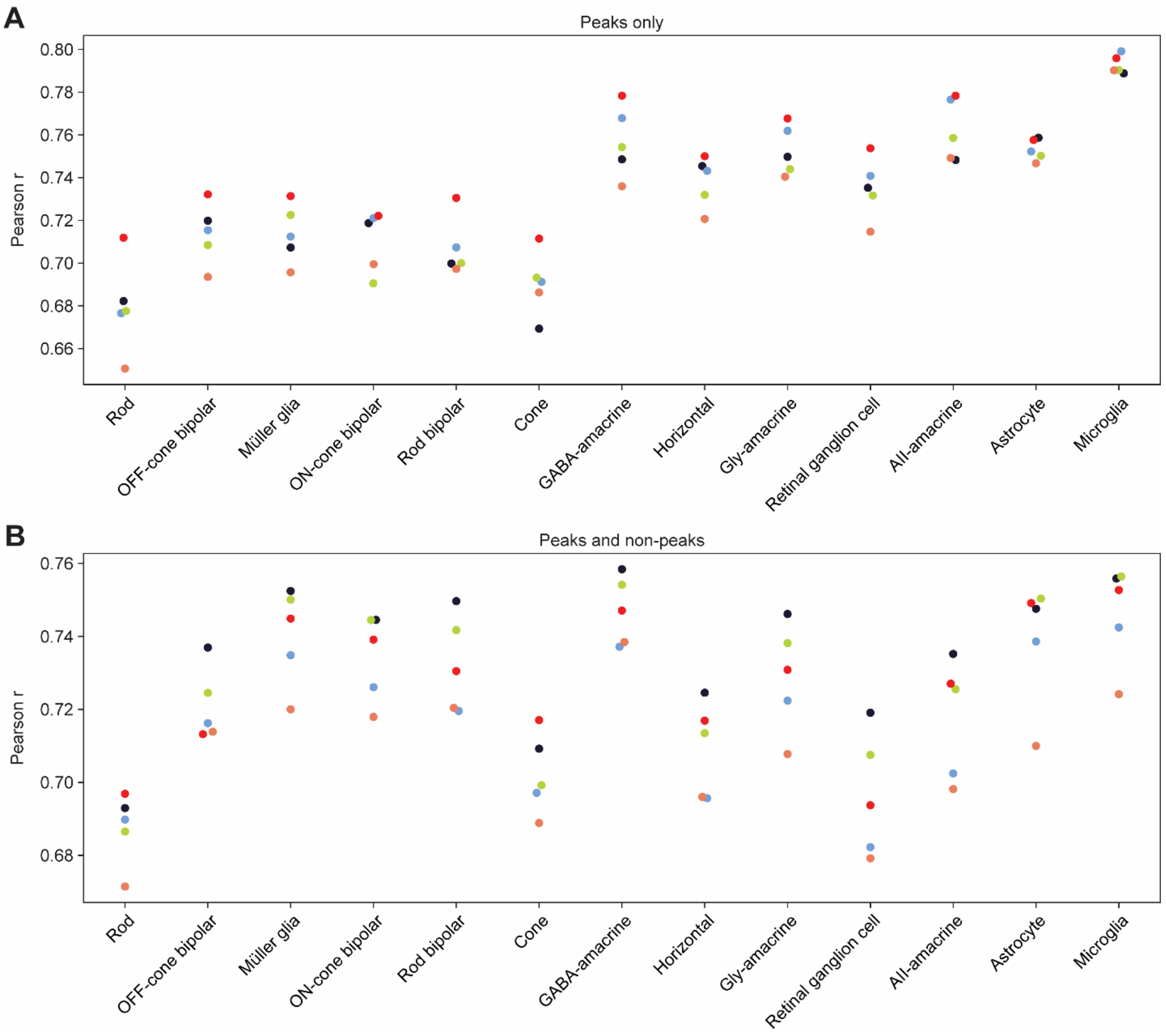

Figure S9. BPNet model performance, Related to Figure 6.

(A, B) Correlation scores between predicted and actual observed log counts in only peak (A) or both peak and non-peak (B) regions on chromosome sequences that were withheld during cell type-specific model training. Each color represents one of five model folds.

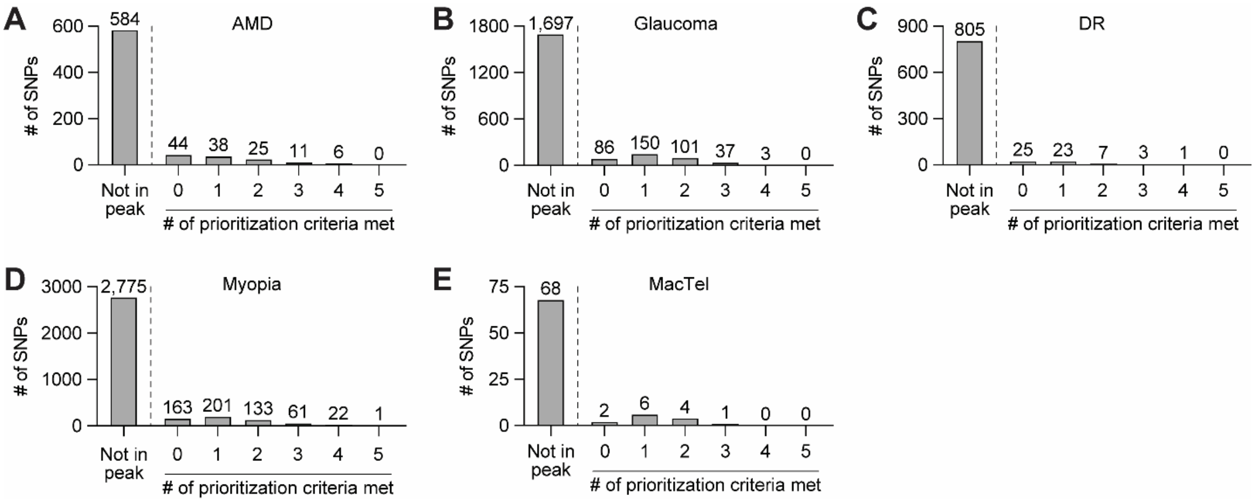

Figure S10. Summary of SNP prioritization, Related to Figures 4-6.

(A-E) Total number of prioritization criteria met by LD expanded SNPs in loci associated with AMD (A), glaucoma (B), DR (C), myopia (D), and MacTel (E). Prioritization criteria were 1) co-accessibility with a promoter peak, 2) accessibility correlated with expression of a nearby gene, 3) linkage to a gene by H3K27ac HiChIP, 4) significant association with a gene by retina eQTL data, and 5) high effect designation by deep learning.

Table S1. Donor information, Related to STAR Methods

ID	Age	Sex	Death-to-preservation interval	Cause of death
LVG1	55	M	9 hours	Cardiac arrest
LGS1	85	F	11 hours	Sepsis
LGS2	87	M	9 hours	Fall
LGS3	74	F	12 hours	Dementia

Table S2. Cell counts per retina, Related to Figure 1

Cell type	LVG1 OD	LVG1 OS	LGS1 OD	LGS1 OS	LGS2 OD	LGS2 OS	LGS3 OD	LGS3 OD
Rod	3151	3061	6132	6777	3409	3190	3299	3040
OFF-cone bipolar	398	373	447	558	557	622	549	432
Müller glia	384	372	496	448	608	559	436	367
ON-cone bipolar	223	245	299	327	449	393	373	308
Rod bipolar	234	190	358	263	350	302	313	314
Cone	197	164	335	386	325	365	240	211
GABA-amacrine	194	135	257	257	169	240	171	120
Horizontal	116	107	114	129	194	124	174	177
Gly-amacrine	83	66	114	138	77	106	80	66
Retinal ganglion cell	98	54	26	20	116	53	100	26
AII-amacrine	42	53	94	72	41	83	43	23
Astrocyte	40	35	4	8	31	19	59	36
Microglia	15	13	33	22	39	51	29	30
Total	5175	4868	8709	9405	6365	6107	5866	5150

Table S3. First author and year of GWAS by disease, Related to Figure 4

AMD	Glaucoma	DR	Myopia	MacTel
Klein 2005[3]	Thorleifsson 2007[4]	Huang 2011[5]	Nakanishi 2009[6]	Scerri 2017[7]
Chen 2010[8]	Meguro 2010[9]	Grassi 2011[10]	Li 2011[11]	Bonelli 2021[12]
Neale 2010[13]	Burdon 2011[14]	Sheu 2013[15]	Li 2011[16]	
Kopplin 2010[17]	Nakano 2012[18]	Awata 2014[19]	Shi 2011[20]	
Yu 2011[21]	Osman 2012[22]	Burdon 2015[23]	Fan 2012[24]	
Arakawa 2011[25]	Wiggs 2012[26]	Graham 2018[27]	Meng 2012[28]	
Cipriani 2012[29]	Takamoto 2012[30]	Meng 2018[31]	Shi 2013[32]	
Sobrin 2012[33]	Vithana 2012[34]	Pollack 2019[35]	Khor 2013[36]	
Holliday 2013[37]	Nakano 2014[38]	Meng 2019[39]	Simpson 2014[40]	
Fritsche 2013[41]	Hoffmann 2014[42]	Liu 2019[43]	Pickrell 2016[44]	
Naj 2013[45]	Chen 2014[46]	Hsieh 2020[47]	Tedja 2018[48]	
Cheng 2015[49]	Gharahkhani 2014[50]	Imamura 2021[51]	Boutin 2020[52]	
Fritsche 2016[53]	Aung 2015[54]		Meguro 2020[55]	
Ruamviboonsuk 2017[56]	Li 2015[57]		Tideman 2021[58]	
Persad 2017[59]	Vishal 2016[60]			
Yan 2018[61]	Khor 2016[62]			
Han 2020[63]	Verma 2016[64]			
Winkler 2020[65]	Bailey 2016[66]			
Guenther 2020[67]	Zagajewska 2018[68]			
	Gharahkhani 2018[69]			
	Choquet 2018[70]			
	MacGregor 2018[71]			
	Bonnemaijer 2018[72]			
	Zhou 2018[73]			
	Shiga 2018[74]			
	Hauser 2019[75]			
	Craig 2020[76]			
	Kim 2020[77]			
	Ishigaki 2020[78]			
	Gharahkhani 2021[79]			
	Sakaue 2021[80]			

SUPPLEMENTAL REFERENCES

1. Lyu, Y., Zauhar, R., Dana, N., Strang, C.E., Hu, J., Wang, K., Liu, S., Pan, N., Gamlin, P., Kimble, J.A., *et al.* (2021). Implication of specific retinal cell-type involvement and gene expression changes in AMD progression using integrative analysis of single-cell and bulk RNA-seq profiling. Sci. Rep. *11*, 15612.

2. Daiger, S.P., Sullivan, L.S., Bowne, S.J. (2013). Genes and mutations causing retinitis pigmentosa. Clin. Genet. *84*, 132–141.

3. Klein, R.J., Zeiss, C., Chew, E.Y., Tsai, J.-Y., Sackler, R.S., Haynes, C., Henning, A.K., SanGiovanni, J.P., Mane, S.M., Mayne, S.T., *et al.* (2005). Complement factor H polymorphism in age-related macular degeneration. Science *308*, 385–9.

4. Thorleifsson, G., Magnusson, K.P., Sulem, P., Walters, G.B., Gudbjartsson, D.F., Stefansson, H., Jonsson, T., Jonasdottir, A., Jonasdottir, A., Stefansdottir, G., *et al.* (2007). Common sequence variants in the LOXL1 gene confer susceptibility to exfoliation glaucoma. Science *317*, 1397–400.

5. Huang, Y.-C., Lin, J.-M., Lin, H.-J., Chen, C.-C., Chen, S.-Y., Tsai, C.-H., Tsai, F.-J. (2011). Genome-wide association study of diabetic retinopathy in a Taiwanese population. Ophthalmology *118*, 642–8.

6. Nakanishi, H., Yamada, R., Gotoh, N., Hayashi, H., Yamashiro, K., Shimada, N., Ohno-Matsui, K., Mochizuki, M., Saito, M., Iida, T., *et al.* (2009). A genome-wide association analysis identified a novel susceptible locus for pathological myopia at 11q24.1. PLoS Genet. *5*, e1000660.

7. Scerri, T.S., Quaglieri, A., Cai, C., Zernant, J., Matsunami, N., Baird, L., Scheppke, L., Bonelli, R., Yannuzzi, L.A., Friedlander, M., *et al.* (2017). Genome-wide analyses identify common variants associated with macular telangiectasia type 2. Nat. Genet. *49*, 559–567.

8. Chen, W., Stambolian, D., Edwards, A.O., Branham, K.E., Othman, M., Jakobsdottir, J., Tosakulwong, N., Pericak-Vance, M.A., Campochiaro, P.A., Klein, M.L., *et al.* (2010). Genetic variants near TIMP3 and high-density lipoprotein-associated loci influence susceptibility to age-related macular degeneration. Proc. Natl. Acad. Sci. U. S. A. *107*, 7401–6.

9. Meguro, A., Inoko, H., Ota, M., Mizuki, N., Bahram, S. (2010). Genome-wide association study of normal tension glaucoma: common variants in SRBD1 and ELOVL5 contribute to disease susceptibility. Ophthalmology *117*, 1331–8.e5.

10. Grassi, M.A., Tikhomirov, A., Ramalingam, S., Below, J.E., Cox, N.J., Nicolae, D.L. (2011). Genome-wide meta-analysis for severe diabetic retinopathy. Hum. Mol. Genet. *20*, 2472–81.

11. Li, Y.-J., Goh, L., Khor, C.-C., Fan, Q., Yu, M., Han, S., Sim, X., Ong, R.T.-H., Wong, T.-Y., Vithana, E.N., *et al.* (2011). Genome-wide association studies reveal genetic variants in CTNND2 for high myopia in Singapore Chinese. Ophthalmology *118*, 368–75.

12. Bonelli, R., Jackson, V.E., Prasad, A., Munro, J.E., Farashi, S., Heeren, T.F.C., Pontikos, N., Scheppke, L., Friedlander, M., MacTel Consortium, *et al.* (2021). Identification of genetic factors influencing metabolic dysregulation and retinal support for MacTel, a retinal disorder. Commun. Biol. *4*, 274.

13. Neale, B.M., Fagerness, J., Reynolds, R., Sobrin, L., Parker, M., Raychaudhuri, S., Tan, P.L., Oh, E.C., Merriam, J.E., Souied, E., *et al.* (2010). Genome-wide association study of advanced age-related macular degeneration identifies a role of the hepatic lipase gene (LIPC). Proc. Natl. Acad. Sci. U. S. A. *107*, 7395–400.

14. Burdon, K.P., Macgregor, S., Hewitt, A.W., Sharma, S., Chidlow, G., Mills, R.A., Danoy, P., Casson, R., Viswanathan, A.C., Liu, J.Z., *et al.* (2011). Genome-wide association study identifies susceptibility loci for open angle glaucoma at TMCO1 and CDKN2B-AS1. Nat. Genet. *43*, 574–8.

15. Sheu, W.H.-H., Kuo, J.Z., Lee, I.-T., Hung, Y.-J., Lee, W.-J., Tsai, H.-Y., Wang, J.-S., Goodarzi, M.O., Klein, R., Klein, B.E.K., *et al.* (2013). Genome-wide association study in a Chinese population with diabetic retinopathy. Hum. Mol. Genet. *22*, 3165–73.

16. Li, Z., Qu, J., Xu, X., Zhou, X., Zou, H., Wang, N., Li, T., Hu, X., Zhao, Q., Chen, P., *et al.* (2011). A genome-wide association study reveals association between common variants in an intergenic region of 4q25 and high-grade myopia in the Chinese Han population. Hum. Mol. Genet. *20*, 2861–8.

17. Kopplin, L.J., Igo, R.P., Wang, Y., Sivakumaran, T.A., Hagstrom, S.A., Peachey, N.S., Francis, P.J., Klein, M.L., SanGiovanni, J.P., Chew, E.Y., *et al.* (2010). Genome-wide association identifies SKIV2L and MYRIP as protective factors for age-related macular degeneration. Genes Immun. *11*, 609–21.

18. Nakano, M., Ikeda, Y., Tokuda, Y., Fuwa, M., Omi, N., Ueno, M., Imai, K., Adachi, H., Kageyama, M., Mori, K., *et al.* (2012). Common variants in CDKN2B-AS1 associated with optic-nerve vulnerability of glaucoma identified by genome-wide association studies in Japanese. PLoS One *7*, e33389.

19. Awata, T., Yamashita, H., Kurihara, S., Morita-Ohkubo, T., Miyashita, Y., Katayama, S., Mori, K., Yoneya, S., Kohda, M., Okazaki, Y., *et al.* (2014). A genome-wide association study for diabetic retinopathy in a Japanese population: potential association with a long intergenic non-coding RNA. PLoS One *9*, e111715.

20. Shi, Y., Qu, J., Zhang, D., Zhao, P., Zhang, Q., Tam, P.O.S., Sun, L., Zuo, X., Zhou, X., Xiao, X., *et al.* (2011). Genetic variants at 13q12.12 are associated with high myopia in the Han Chinese population. Am. J. Hum. Genet. *88*, 805–813.

21. Yu, Y., Bhangale, T.R., Fagerness, J., Ripke, S., Thorleifsson, G., Tan, P.L., Souied, E.H., Richardson, A.J., Merriam, J.E., Buitendijk, G.H.S., *et al.* (2011). Common variants near FRK/COL10A1 and VEGFA are associated with advanced age-related macular degeneration. Hum. Mol. Genet. *20*, 3699–709.

22. Osman, W., Low, S.-K., Takahashi, A., Kubo, M., Nakamura, Y. (2012). A genome-wide association study in the Japanese population confirms 9p21 and 14q23 as susceptibility loci for primary open angle glaucoma. Hum. Mol. Genet. *21*, 2836–42.

23. Burdon, K.P., Fogarty, R.D., Shen, W., Abhary, S., Kaidonis, G., Appukuttan, B., Hewitt, A.W., Sharma, S., Daniell, M., Essex, R.W., *et al.* (2015). Genome-wide association study for sight-threatening diabetic retinopathy reveals association with genetic variation near the GRB2 gene. Diabetologia *58*, 2288–97.

24. Fan, Q., Barathi, V.A., Cheng, C.-Y., Zhou, X., Meguro, A., Nakata, I., Khor, C.-C., Goh, L.-

K., Li, Y.-J., Lim, W., *et al.* (2012). Genetic variants on chromosome 1q41 influence ocular axial length and high myopia. PLoS Genet. *8*, e1002753.

25. Arakawa, S., Takahashi, A., Ashikawa, K., Hosono, N., Aoi, T., Yasuda, M., Oshima, Y., Yoshida, S., Enaida, H., Tsuchihashi, T., *et al.* (2011). Genome-wide association study identifies two susceptibility loci for exudative age-related macular degeneration in the Japanese population. Nat. Genet. *43*, 1001–4.

26. Wiggs, J.L., Yaspan, B.L., Hauser, M.A., Kang, J.H., Allingham, R.R., Olson, L.M., Abdrabou, W., Fan, B.J., Wang, D.Y., Brodeur, W., *et al.* (2012). Common variants at 9p21 and 8q22 are associated with increased susceptibility to optic nerve degeneration in glaucoma. PLoS Genet. *8*, e1002654.

27. Graham, P.S., Kaidonis, G., Abhary, S., Gillies, M.C., Daniell, M., Essex, R.W., Chang, J.H., Lake, S.R., Pal, B., Jenkins, A.J., *et al.* (2018). Genome-wide association studies for diabetic macular edema and proliferative diabetic retinopathy. BMC Med. Genet. *19*, 71.

28. Meng, W., Butterworth, J., Bradley, D.T., Hughes, A.E., Soler, V., Calvas, P., Malecaze, F. (2012). A genome-wide association study provides evidence for association of chromosome 8p23 (MYP10) and 10q21.1 (MYP15) with high myopia in the French Population. Invest. Ophthalmol. Vis. Sci. *53*, 7983–8.

29. Cipriani, V., Leung, H.-T., Plagnol, V., Bunce, C., Khan, J.C., Shahid, H., Moore, A.T., Harding, S.P., Bishop, P.N., Hayward, C., *et al.* (2012). Genome-wide association study of age-related macular degeneration identifies associated variants in the TNXB-FKBPL-NOTCH4 region of chromosome 6p21.3. Hum. Mol. Genet. *21*, 4138–50.

30. Takamoto, M., Kaburaki, T., Mabuchi, A., Araie, M., Amano, S., Aihara, M., Tomidokoro, A., Iwase, A., Mabuchi, F., Kashiwagi, K., *et al.* (2012). Common variants on chromosome 9p21 are associated with normal tension glaucoma. PLoS One *7*, e40107.

31. Meng, W., Shah, K.P., Pollack, S., Toppila, I., Hebert, H.L., McCarthy, M.I., Groop, L., Ahlqvist, E., Lyssenko, V., Agardh, E., *et al.* (2018). A genome-wide association study suggests new evidence for an association of the NADPH Oxidase 4 (NOX4) gene with severe diabetic retinopathy in type 2 diabetes. Acta Ophthalmol. *96*, e811–e819.

32. Shi, Y., Gong, B., Chen, L., Zuo, X., Liu, X., Tam, P.O.S., Zhou, X., Zhao, P., Lu, F., Qu, J., *et al.* (2013). A genome-wide meta-analysis identifies two novel loci associated with high myopia in the Han Chinese population. Hum. Mol. Genet. *22*, 2325–33.

33. Sobrin, L., Ripke, S., Yu, Y., Fagerness, J., Bhangale, T.R., Tan, P.L., Souied, E.H., Buitendijk, G.H.S., Merriam, J.E., Richardson, A.J., *et al.* (2012). Heritability and genome-wide association study to assess genetic differences between advanced age-related macular degeneration subtypes. Ophthalmology *119*, 1874–85.

34. Vithana, E.N., Khor, C.-C., Qiao, C., Nongpiur, M.E., George, R., Chen, L.-J., Do, T., Abu-Amero, K., Huang, C.K., Low, S., *et al.* (2012). Genome-wide association analyses identify three new susceptibility loci for primary angle closure glaucoma. Nat. Genet. *44*, 1142–1146.

35. Pollack, S., Igo, R.P., Jensen, R.A., Christiansen, M., Li, X., Cheng, C.-Y., Ng, M.C.Y., Smith, A. V, Rossin, E.J., Segrè, A. V, *et al.* (2019). Multiethnic Genome-Wide Association Study of Diabetic Retinopathy Using Liability Threshold Modeling of Duration of Diabetes and Glycemic Control. Diabetes *68*, 441–456.

36. Khor, C.C., Miyake, M., Chen, L.J., Shi, Y., Barathi, V.A., Qiao, F., Nakata, I., Yamashiro, K., Zhou, X., Tam, P.O.S., *et al.* (2013). Genome-wide association study identifies ZFHX1B as a susceptibility locus for severe myopia. Hum. Mol. Genet. *22*, 5288–94.

37. Holliday, E.G., Smith, A. V, Cornes, B.K., Buitendijk, G.H.S., Jensen, R.A., Sim, X., Aspelund, T., Aung, T., Baird, P.N., Boerwinkle, E., *et al.* (2013). Insights into the genetic architecture of early stage age-related macular degeneration: a genome-wide association study meta-analysis. PLoS One *8*, e53830.

38. Nakano, M., Ikeda, Y., Tokuda, Y., Fuwa, M., Ueno, M., Imai, K., Sato, R., Omi, N., Adachi, H., Kageyama, M., *et al.* (2014). Novel common variants and susceptible haplotype for exfoliation glaucoma specific to Asian population. Sci. Rep. *4*, 5340.

39. Meng, W., Chan, B.W., Ezeonwumelu, C., Hébert, H.L., Campbell, A., Soler, V., Palmer, C.N. (2019). A genome-wide association study implicates that the TTC39C gene is associated with diabetic maculopathy with decreased visual acuity. Ophthalmic Genet. *40*, 252–258.

40. Simpson, C.L., Wojciechowski, R., Oexle, K., Murgia, F., Portas, L., Li, X., Verhoeven, V.J.M., Vitart, V., Schache, M., Hosseini, S.M., *et al.* (2014). Genome-wide meta-analysis of myopia and hyperopia provides evidence for replication of 11 loci. PLoS One *9*, e107110.

41. Fritsche, L.G., Chen, W., Schu, M., Yaspan, B.L., Yu, Y., Thorleifsson, G., Zack, D.J., Arakawa, S., Cipriani, V., Ripke, S., *et al.* (2013). Seven new loci associated with age-related macular degeneration. Nat. Genet. *45*, 433–9, 439e1-2.

42. Hoffmann, T.J., Tang, H., Thornton, T.A., Caan, B., Haan, M., Millen, A.E., Thomas, F., Risch, N. (2014). Genome-wide association and admixture analysis of glaucoma in the Women's Health Initiative. Hum. Mol. Genet. *23*, 6634–43.

43. Liu, C., Chen, G., Bentley, A.R., Doumatey, A., Zhou, J., Adeyemo, A., Yang, J., Rotimi, C. (2019). Genome-wide association study for proliferative diabetic retinopathy in Africans. NPJ genomic Med. *4*, 20.

44. Pickrell, J.K., Berisa, T., Liu, J.Z., Ségurel, L., Tung, J.Y., Hinds, D.A. (2016). Detection and interpretation of shared genetic influences on 42 human traits. Nat. Genet. *48*, 709–17.

45. Naj, A.C., Scott, W.K., Courtenay, M.D., Cade, W.H., Schwartz, S.G., Kovach, J.L., Agarwal, A., Wang, G., Haines, J.L., Pericak-Vance, M.A. (2013). Genetic factors in nonsmokers with age-related macular degeneration revealed through genome-wide gene-environment interaction analysis. Ann. Hum. Genet. *77*, 215–31.

46. Chen, Y., Lin, Y., Vithana, E.N., Jia, L., Zuo, X., Wong, T.Y., Chen, L.J., Zhu, X., Tam, P.O.S., Gong, B., *et al.* (2014). Common variants near ABCA1 and in PMM2 are associated with primary open-angle glaucoma. Nat. Genet. *46*, 1115–9.

47. Hsieh, A.-R., Huang, Y.-C., Yang, Y.-F., Lin, H.-J., Lin, J.-M., Chang, Y.-W., Wu, C.-M., Liao, W.-L., Tsai, F.-J. (2020). Lack of association of genetic variants for diabetic retinopathy in Taiwanese patients with diabetic nephropathy. BMJ open diabetes Res. care *8*.

48. Tedja, M.S., Wojciechowski, R., Hysi, P.G., Eriksson, N., Furlotte, N.A., Verhoeven, V.J.M., Iglesias, A.I., Meester-Smoor, M.A., Tompson, S.W., Fan, Q., *et al.* (2018). Genome-wide association meta-analysis highlights light-induced signaling as a driver for refractive error. Nat. Genet. *50*, 834–848.

49. Cheng, C.-Y., Yamashiro, K., Chen, L.J., Ahn, J., Huang, L., Huang, L., Cheung, C.M.G., Miyake, M., Cackett, P.D., Yeo, I.Y., *et al.* (2015). New loci and coding variants confer risk for age-related macular degeneration in East Asians. Nat. Commun. *6*, 6063.

50. Gharahkhani, P., Burdon, K.P., Fogarty, R., Sharma, S., Hewitt, A.W., Martin, S., Law, M.H., Cremin, K., Bailey, J.N.C., Loomis, S.J., *et al.* (2014). Common variants near ABCA1, AFAP1 and GMDS confer risk of primary open-angle glaucoma. Nat. Genet. *46*, 1120–1125.

51. Imamura, M., Takahashi, A., Matsunami, M., Horikoshi, M., Iwata, M., Araki, S.-I., Toyoda, M., Susarla, G., Ahn, J., Park, K.H., *et al.* (2021). Genome-wide association studies identify two novel loci conferring susceptibility to diabetic retinopathy in Japanese patients with type 2 diabetes. Hum. Mol. Genet. *30*, 716–726.

52. Boutin, T.S., Charteris, D.G., Chandra, A., Campbell, S., Hayward, C., Campbell, A., UK Biobank Eye & Vision Consortium, Nandakumar, P., Hinds, D., 23andMe Research Team, *et al.* (2020). Insights into the genetic basis of retinal detachment. Hum. Mol. Genet. *29*, 689–702.

53. Fritsche, L.G., Igl, W., Bailey, J.N.C., Grassmann, F., Sengupta, S., Bragg-Gresham, J.L., Burdon, K.P., Hebbring, S.J., Wen, C., Gorski, M., *et al.* (2016). A large genome-wide association study of age-related macular degeneration highlights contributions of rare and common variants. Nat. Genet. *48*, 134–43.

54. Aung, T., Ozaki, M., Mizoguchi, T., Allingham, R.R., Li, Z., Haripriya, A., Nakano, S., Uebe, S., Harder, J.M., Chan, A.S.Y., *et al.* (2015). A common variant mapping to CACNA1A is associated with susceptibility to exfoliation syndrome. Nat. Genet. *47*, 387–92.

55. Meguro, A., Yamane, T., Takeuchi, M., Miyake, M., Fan, Q., Zhao, W., Wang, I.-J., Mizuki, Y., Yamada, N., Nomura, N., *et al.* (2020). Genome-Wide Association Study in Asians Identifies Novel Loci for High Myopia and Highlights a Nervous System Role in Its Pathogenesis. Ophthalmology *127*, 1612–1624.

56. Ruamviboonsuk, P., Tadarati, M., Singhanetr, P., Wattanapokayakit, S., Kunhapan, P., Wanitchanon, T., Wichukchinda, N., Mushiroda, T., Akiyama, M., Momozawa, Y., *et al.* (2017). Genome-wide association study of neovascular age-related macular degeneration in the Thai population. J. Hum. Genet. *62*, 957–962.

57. Li, Z., Allingham, R.R., Nakano, M., Jia, L., Chen, Y., Ikeda, Y., Mani, B., Chen, L.-J., Kee, C., Garway-Heath, D.F., *et al.* (2015). A common variant near TGFBR3 is associated with primary open angle glaucoma. Hum. Mol. Genet. *24*, 3880–92.

58. Tideman, J.W.L., Pärssinen, O., Haarman, A.E.G., Khawaja, A.P., Wedenoja, J., Williams, K.M., Biino, G., Ding, X., Kähönen, M., Lehtimäki, T., *et al.* (2021). Evaluation of Shared Genetic Susceptibility to High and Low Myopia and Hyperopia. JAMA Ophthalmol. *139*, 601–609.

59. Persad, P.J., Heid, I.M., Weeks, D.E., Baird, P.N., de Jong, E.K., Haines, J.L., Pericak-Vance, M.A., Scott, W.K., International Age-Related Macular Degeneration Genomics Consortium (IAMDGC) (2017). Joint Analysis of Nuclear and Mitochondrial Variants in Age-Related Macular Degeneration Identifies Novel Loci TRPM1 and ABHD2/RLBP1. Invest. Ophthalmol. Vis. Sci. *58*, 4027–4038.

60. Vishal, M., Sharma, A., Kaurani, L., Alfano, G., Mookherjee, S., Narta, K., Agrawal, J., Bhattacharya, I., Roychoudhury, S., Ray, J., *et al.* (2016). Genetic association and stress

mediated down-regulation in trabecular meshwork implicates MPP7 as a novel candidate gene in primary open angle glaucoma. BMC Med. Genomics *9*, 15.

61. Yan, Q., Ding, Y., Liu, Y., Sun, T., Fritsche, L.G., Clemons, T., Ratnapriya, R., Klein, M.L., Cook, R.J., Liu, Y., *et al.* (2018). Genome-wide analysis of disease progression in age-related macular degeneration. Hum. Mol. Genet. *27*, 929–940.

62. Khor, C.C., Do, T., Jia, H., Nakano, M., George, R., Abu-Amero, K., Duvesh, R., Chen, L.J., Li, Z., Nongpiur, M.E., *et al.* (2016). Genome-wide association study identifies five new susceptibility loci for primary angle closure glaucoma. Nat. Genet. *48*, 556–62.

63. Han, X., Gharahkhani, P., Mitchell, P., Liew, G., Hewitt, A.W., MacGregor, S. (2020). Genome-wide meta-analysis identifies novel loci associated with age-related macular degeneration. J. Hum. Genet. *65*, 657–665.

64. Verma, S.S., Cooke Bailey, J.N., Lucas, A., Bradford, Y., Linneman, J.G., Hauser, M.A., Pasquale, L.R., Peissig, P.L., Brilliant, M.H., McCarty, C.A., *et al.* (2016). Epistatic Gene-Based Interaction Analyses for Glaucoma in eMERGE and NEIGHBOR Consortium. PLoS Genet. *12*, e1006186.

65. Winkler, T.W., Grassmann, F., Brandl, C., Kiel, C., Günther, F., Strunz, T., Weidner, L., Zimmermann, M.E., Korb, C.A., Poplawski, A., *et al.* (2020). Genome-wide association meta-analysis for early age-related macular degeneration highlights novel loci and insights for advanced disease. BMC Med. Genomics *13*, 120.

66. Bailey, J.N.C., Loomis, S.J., Kang, J.H., Allingham, R.R., Gharahkhani, P., Khor, C.C., Burdon, K.P., Aschard, H., Chasman, D.I., Igo, R.P., *et al.* (2016). Genome-wide association analysis identifies TXNRD2, ATXN2 and FOXC1 as susceptibility loci for primary open-angle glaucoma. Nat. Genet. *48*, 189–94.

67. Guenther, F., Brandl, C., Winkler, T.W., Wanner, V., Stark, K., Kuechenhoff, H., Heid, I.M. (2020). Chances and challenges of machine learning-based disease classification in genetic association studies illustrated on age-related macular degeneration. Genet. Epidemiol. *44*, 759–777.

68. Zagajewska, K., Piątkowska, M., Goryca, K., Bałabas, A., Kluska, A., Paziewska, A., Pośpiech, E., Grabska-Liberek, I., Hennig, E.E. (2018). GWAS links variants in neuronal development and actin remodeling related loci with pseudoexfoliation syndrome without glaucoma. Exp. Eye Res. *168*, 138–148.

69. Gharahkhani, P., Burdon, K.P., Cooke Bailey, J.N., Hewitt, A.W., Law, M.H., Pasquale, L.R., Kang, J.H., Haines, J.L., Souzeau, E., Zhou, T., *et al.* (2018). Analysis combining correlated glaucoma traits identifies five new risk loci for open-angle glaucoma. Sci. Rep. *8*, 3124.

70. Choquet, H., Paylakhi, S., Kneeland, S.C., Thai, K.K., Hoffmann, T.J., Yin, J., Kvale, M.N., Banda, Y., Tolman, N.G., Williams, P.A., *et al.* (2018). A multiethnic genome-wide association study of primary open-angle glaucoma identifies novel risk loci. Nat. Commun. *9*, 2278.

71. MacGregor, S., Ong, J.-S., An, J., Han, X., Zhou, T., Siggs, O.M., Law, M.H., Souzeau, E., Sharma, S., Lynn, D.J., *et al.* (2018). Genome-wide association study of intraocular pressure uncovers new pathways to glaucoma. Nat. Genet. *50*, 1067–1071.

72. Bonnemaijer, P.W.M., Iglesias, A.I., Nadkarni, G.N., Sanyiwa, A.J., Hassan, H.G., Cook, C., GIGA Study Group, Simcoe, M., Taylor, K.D., Schurmann, C., *et al.* (2018). Genome-wide association study of primary open-angle glaucoma in continental and admixed African populations. Hum. Genet. *137*, 847–862.

73. Zhou, W., Nielsen, J.B., Fritsche, L.G., Dey, R., Gabrielsen, M.E., Wolford, B.N., LeFaive, J., VandeHaar, P., Gagliano, S.A., Gifford, A., *et al.* (2018). Efficiently controlling for case-control imbalance and sample relatedness in large-scale genetic association studies. Nat. Genet. *50*, 1335–1341.

74. Shiga, Y., Akiyama, M., Nishiguchi, K.M., Sato, K., Shimozawa, N., Takahashi, A., Momozawa, Y., Hirata, M., Matsuda, K., Yamaji, T., *et al.* (2018). Genome-wide association study identifies seven novel susceptibility loci for primary open-angle glaucoma. Hum. Mol. Genet. *27*, 1486–1496.

75. Hauser, M.A., Allingham, R.R., Aung, T., Van Der Heide, C.J., Taylor, K.D., Rotter, J.I., Wang, S.-H.J., Bonnemaijer, P.W.M., Williams, S.E., Abdullahi, S.M., *et al.* (2019). Association of Genetic Variants With Primary Open-Angle Glaucoma Among Individuals With African Ancestry. JAMA *322*, 1682–1691.

76. Craig, J.E., Han, X., Qassim, A., Hassall, M., Cooke Bailey, J.N., Kinzy, T.G., Khawaja, A.P., An, J., Marshall, H., Gharahkhani, P., *et al.* (2020). Multitrait analysis of glaucoma identifies new risk loci and enables polygenic prediction of disease susceptibility and progression. Nat. Genet. *52*, 160–166.

77. Kim, Y.W., Kim, Y.J., Cheong, H.S., Shiga, Y., Hashimoto, K., Song, Y.J., Kim, S.H., Choi, H.J., Nishiguchi, K.M., Kawai, Y., *et al.* (2020). Exploring the Novel Susceptibility Gene Variants for Primary Open-Angle Glaucoma in East Asian Cohorts: The GLAU-GENDISK Study. Sci. Rep. *10*, 221.

78. Ishigaki, K., Akiyama, M., Kanai, M., Takahashi, A., Kawakami, E., Sugishita, H., Sakaue, S., Matoba, N., Low, S.-K., Okada, Y., *et al.* (2020). Large-scale genome-wide association study in a Japanese population identifies novel susceptibility loci across different diseases. Nat. Genet. *52*, 669–679.

79. Gharahkhani, P., Jorgenson, E., Hysi, P., Khawaja, A.P., Pendergrass, S., Han, X., Ong, J.S., Hewitt, A.W., Segrè, A. V, Rouhana, J.M., *et al.* (2021). Genome-wide meta-analysis identifies 127 open-angle glaucoma loci with consistent effect across ancestries. Nat. Commun. *12*, 1258.

80. Sakaue, S., Kanai, M., Tanigawa, Y., Karjalainen, J., Kurki, M., Koshiba, S., Narita, A., Konuma, T., Yamamoto, K., Akiyama, M., *et al.* (2021). A cross-population atlas of genetic associations for 220 human phenotypes. Nat. Genet. *53*, 1415–1424.
